# Useful Access to Uncommon Thiazolo[3,2-*a*]indoles

**DOI:** 10.1021/acs.joc.3c02338

**Published:** 2024-01-09

**Authors:** Giacomo Mari, Lucia De Crescentini, Gianfranco Favi, Amalija Golobič, Stefania Santeusanio, Fabio Mantellini

**Affiliations:** † Department of Biomolecular Sciences, Section of Chemistry and Pharmaceutical Technologies, University of Urbino “Carlo Bo”, Via I Maggetti 24, 61029 Urbino (PU), Italy; ‡ Faculty of Chemistry and Chemical Technology, University of Ljubljana, Večna pot 113, 1000 Ljubljana, Slovenia

## Abstract

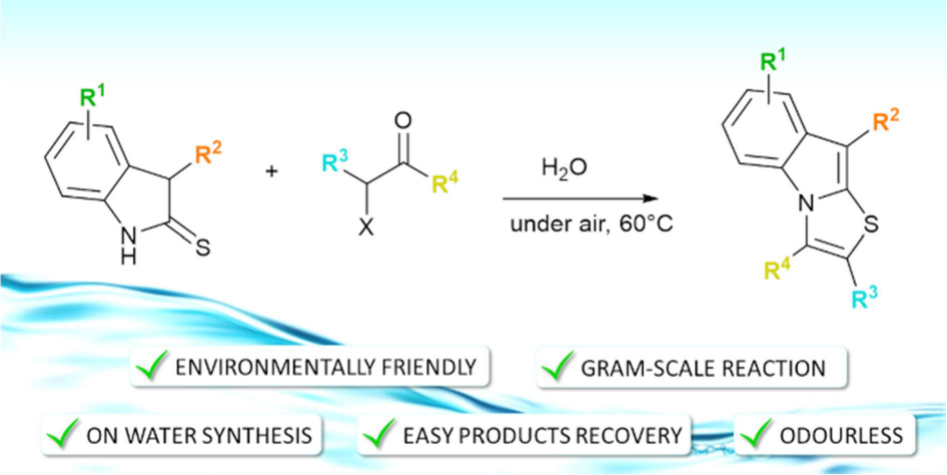

A practical and environmentally benign protocol for the assembly of poly substituted-thiazolo[3,2-*a*]indoles from 3-alkylated indoline-2-thiones and 2-halo-ketones has been developed. This metal-free approach consists in a complete chemo/regioselective formal [3 + 2] annulation that occurs in air, at 60 °C, and in water as the sole reaction medium. The opportunity to vary the substitution pattern up to six different positions, odorless manipulation of sulfurylated compounds, very easy product isolation, and mild reaction conditions are the main synthetic features of this method. The scaled-up experiment and the successive transformations of the products further demonstrate the utility of this chemistry.

## Introduction

The indole core fused with carbo or hetero rings constitutes a recurring framework of derivatives endowed with significant biological and medicinal properties and therefore subject of considerable attention.^1^ In this context, interesting examples are represented by the indole-annulated heterocycles containing sulfur atoms that display relevant properties such as antifungal,^2^ anticancer,^3^ anti-inflamatory,^4^ antihyperplasia,^5^ analgesic,^6^ antibacterial ones,^7^ antipsychotic activities^8^ or that were employed in the construction of small efficient push–pull chromophores.^9^ They are also found in several cruciferous phytoalexins, e.g., cyclobrassinin, spiro-brassinin, or brassicanal B, employed as plant defense chemicals.^10^ In particular, thiazolo[3,2-*a*]indoles are also biologically relevant for their potential activity as 5HT_4_ receptor antagonists.^11^ Although several methods devoted to the preparation of 2,3-thiophene-fused indoles are reported in the literature,^12^ the approaches to their isomer 1,2-fused indoles such as the thiazolo[3,2-*a*]indoles are very limited. Clearly, this occurrence is due to the lower nucleophilic nature of indole nitrogen compared to that expressed by the carbon in position 3.

**Figure 1:**
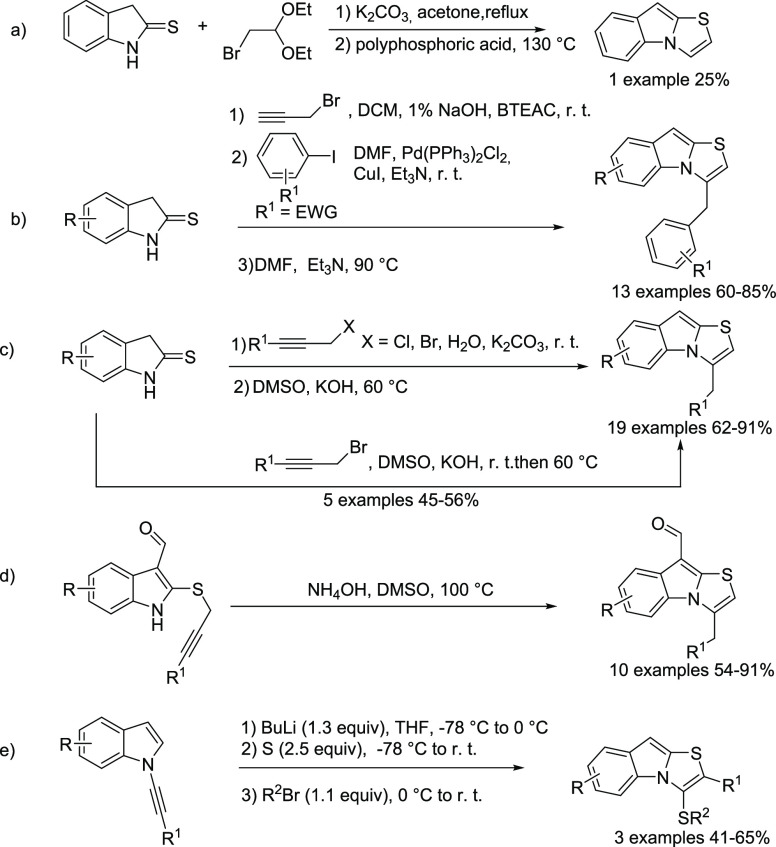
(a–e) Summary of synthetic approaches known to date of thiazolo[3,2-*a*]indoles.

The few synthetic processes currently available can be easily summarized: Gaster and Wyman developed the reaction under basic conditions of the indoline-2-thione with bromoacetaldehyde diethyl acetal followed by treatment with polyphosphoric acid.^11^ In this case, only the unsubstituted thiazolo[3,2-*a*]indole was obtained and in a very low yield (Figure 1a). The synthesis of benzylated thiazoloindoles proposed by Majumdar proceeds by Sonogashira acetylide coupling, followed by a triethylamine-induced regioselective cyclization sequence (Figure 1b).^13^ The group of Jha planned a strategy that involves first the formation of 2-(prop-2-ynylthio)-1*H*-indole intermediates, which undergo base-mediated intramolecular hydroamination (Figure 1c).^14^ One limitation of both these latter two approaches is related to the use of thermally labile 2-(prop-2-ynylthio)-1*H*-indoles that easily undergo photo- and thermal decomposition.^14^

Successively, the same group of Jha demonstrated that 3-formyl substitutions on these intermediates facilitated the synthesis of *N*-fused heterocycles including the thiazolo[3,2-*a*]indole,^15^ obtained by treatment of the 3-formyl-2-(prop-2-ynylthio)-1*H*-indoles with five equivalents of ammonium hydroxide in dimethyl sulfoxide at 100 °C (Figure 1d). Finally, Zeni described the synthesis of 3-(organosulfuryl)thiazole indoles by combining *N*-alkynylindoles, *n*-butyllithium, elemental sulfur, and an electrophile source (Figure 1e).^16^ As described, several limitations are associated with these approaches: complicated and laborious workup or harsh reaction conditions are frequently required; strong acids or strong bases incompatible with different functional groups are often necessary; starting materials that are difficult to prepare and not readily available or very labile reaction intermediates are often involved.

**Figure 2:**
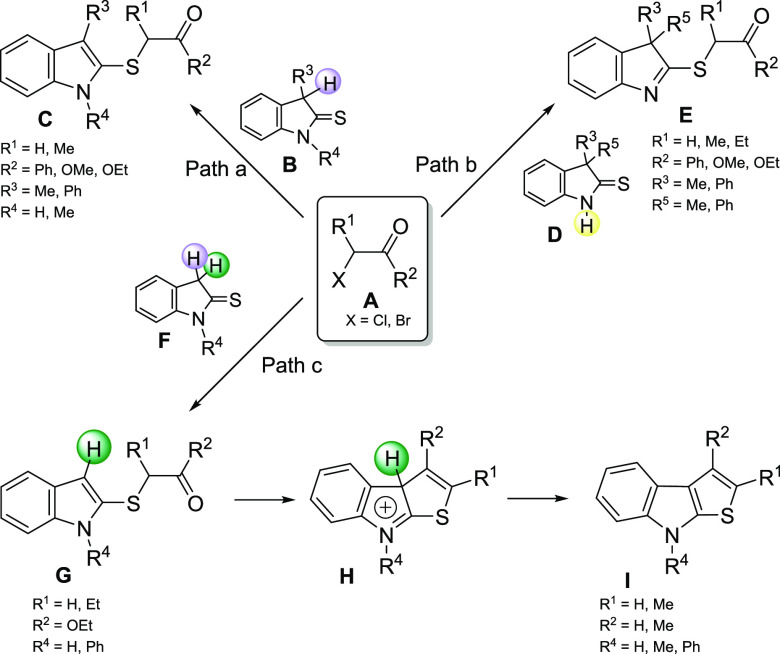
Reactivity of differently substituted indoline-2-thiones**B,D,F** toward 2-halo-carbonyl compounds **A**.

Furthermore, in developing these synthetic strategies, their environmental impact was often not adequately treated. In recent years, environmental consciousness has notably grown, and with it, the awareness of considering sustainability among the various parameters to be optimized for a synthetic process has also increased. The solvents employed for the reaction and in the usual related manipulation operations constitute the component of a process that majorly impacts cost, safety, and sustainability. The use of water as a reaction medium as well as the reduction of the environmental impact of organic synthesis can also benefit chemical processes by simplifying operations, allowing mild reaction conditions, and sometimes delivering unexpected reactivities.^17^ Considering the prominence of thiazolo[3,2-*a*]indoles, and the very limited number of suitable methods available for their preparation, the development of environmentally friendly synthetic approaches to efficiently access these sulfurylated polyheterocycles is of remarkable interest. In this context, a literature survey reveals that Nishio thoroughly investigated the behavior of variously substituted indoline-2-thiones **B,F,D** toward α-halo esters and α-halo ketones **A**, demonstrating that the sulfur atom acts as a nucleophile, providing the corresponding alkylthio derivatives **C,E,G** (Figure 2).^18^ For the indoline-2-thiones **B,F** bearing at least one hydrogen in position 3 (Figure 2, paths a and c), the concomitant aromatization derived from the thione-thioenol tautomerism drives the regioselectivity of the reaction. On the other hand, it was also demonstrated that employing 3,3-disubstituted indoline-2-thiones **D**, the sulfur atom can be activated by the nitrogen atom through the thioamide-imidothiol tautomerism (Figure 2, path b).^18,19^ More recently, Boeini has developed a very simple access to 2,3-thiophene-fused indoles **I** by means of the reaction between 3-unsubstituted indoline-2-thiones **F** and α-halo carbonyl derivatives **A** (Figure 2, path c).^20^ In these conditions, the 2-alkylthio indole intermediates **G** (obtained thanks to the loss of the hydrogen highlighted in violet on C3) spontaneously cyclize to the 3a*H*-thieno[2,3-*b*]indol-8-ium intermediate **H**, and the loss of the second proton (highlighted in green) permits the rearomatization of the thiophene nucleus, producing the final thieno[2,3-*b*]indoles **I** (Figure 2, path c).

**Figure 3:**
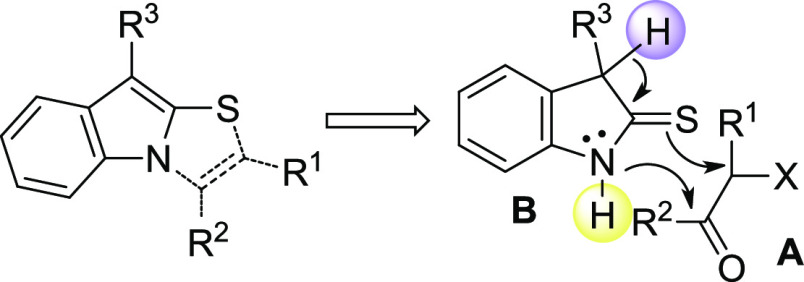
Necessary structural equirements to obtain 1,2-fused indole cores from indoline-2-thiones.

This set of data clearly indicate that the substitution on the C3 of indoline-2-thiones is crucial to determine the regioselectivity of the cyclization process. With the aim of synthesizing the 1,2-fused indole cores such as the thiazolo[3,2-*a*]indoles, a quick retrosynthetic analysis reveals that the partner of the 2-halo-carbonyl compounds **A** should be C3-monosubstituted indoline-2-thiones **B**: a single hydrogen should be able to activate the nucleophilicity of the sulfur and, at the same time, to avoid the rearomatization process consequent to the successive nucleophilic attack of the indolic-C3, thus promoting the *N*-cyclization process (Figure 3).

Herein, we will verify the feasibility of this approach.

## Results and Discussion

Our investigation began by conducting the reaction in dichloromethane, at room temperature between 3-methylindoline-2-thione **1a**(21) and ethyl 2-chloroacetoacetate **2a**, chosen as a model system (Table 1, entry 1). Although the reaction after 48 h was not complete, with our great satisfaction, we noticed that the desired thiazolo[3,2-*a*]indole **3a** was obtained in a 23% yield. Employing chloroform at room temperature, a similar behavior was recorded, while raising the temperature until reflux, the reaction ended in 8 h, but its profile was more complicated due to the formation of several other unidentified byproducts (Table 1, entries 2 and 3). Further attempts to improve the yield by adding bases such as potassium carbonate, triethylamine (TEA), and pyridine to neutralize the hydrochloric acid released during the reaction (Table 1, entries 4–6) proved to be unfruitful. Testing several solvents, such as acetonitrile (ACN), methanol, ethanol, and water, we realized that the latter provides the best outcome (Table 1, entries 7–10). Increasing the amount of ethyl 2-chloroacetoacetate **2a**, no substantial variations were observed (Table 1, entries 11 and 12), while, by heating the reaction, the yields increase, and it was registered that to obtain the complete conversion of the starting reactants, it is necessary to reach 60 °C (Table 1, entries 13 and 14). Under these conditions, the desired thiazolo[3,2-*a*]indole **3a** was obtained quantitatively in 5.5 h. It is noteworthy that the reaction occurs under air, and pure **3a** was recovered purely by simple extraction with ethyl acetate from the reaction crude, without having to resort to any further purification, thus minimizing the solvent, energy, and workup requirements.

**Table 1:**
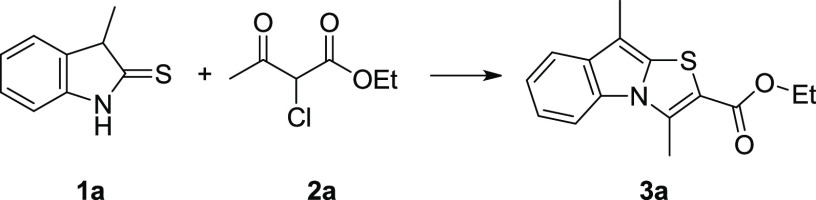
Optimization Studies for the Reaction between 3-Methylindoline-2-thione **1a** and Ethyl 2-Chloroacetoacetate **2a** To Achieve Thiazolo[3,2-*a*]indole **3a**

entry^a^	solvent	equiv of 2a	bases	temp. (°C)	time (h)	yield of **3a**^b^
1^c^	DCM	1.0		r.t.	48.0	23
2^c^	CHCl_3_	1.0		r.t.	48.0	18
3	CHCl_3_	1.0		reflux	8.0	16
4^c^^,^^d^	DCM	1.0	K_2_CO_3_	r.t.	48.0	20
5^c^^,^^e^	DCM	1.0	TEA	r.t.	48.0	19
6^c^^,^^e^	DCM	1.0	pyridine	r.t.	48.0	16
7^c^	ACN	1.0		r.t.	48.0	23
8^c^	MeOH	1.0		r.t.	48.0	36
9^c^	EtOH	1.0		r.t.	48.0	35
10^c^	H_2_O	1.0		r.t.	48.0	48
11^c^	H_2_O	1.5		r.t.	48.0	52
12^c^	H_2_O	3.0		r.t.	48.0	50
13^c^	H_2_O	1.0		40	48.0	73
**14**	**H**_**2**_**O**	**1.0**		**60**	**5.5**	**97**

a The reactions were conducted on a 0.7 mmol scale referred to **1a** in 1 mL of solvent.

b Isolated yields of thiazolo[3,2-a]indole **3a** calculated on 3-methylindoline-2-thione **1a**.

c Part of 3-methylindoline-2-thione **1a** was recovered unreacted.

d 4 equiv of potassium carbonate were added.

e 1.5 equiv of base were added.

The analogous brominated ethyl acetoacetate **2a’** (please see Scheme S2, Supporting Information), under the discovered optimized conditions, furnished the thiazolo[3,2-*a*]indole **3a** with a comparable yield (Table 2).

**Table 2:**
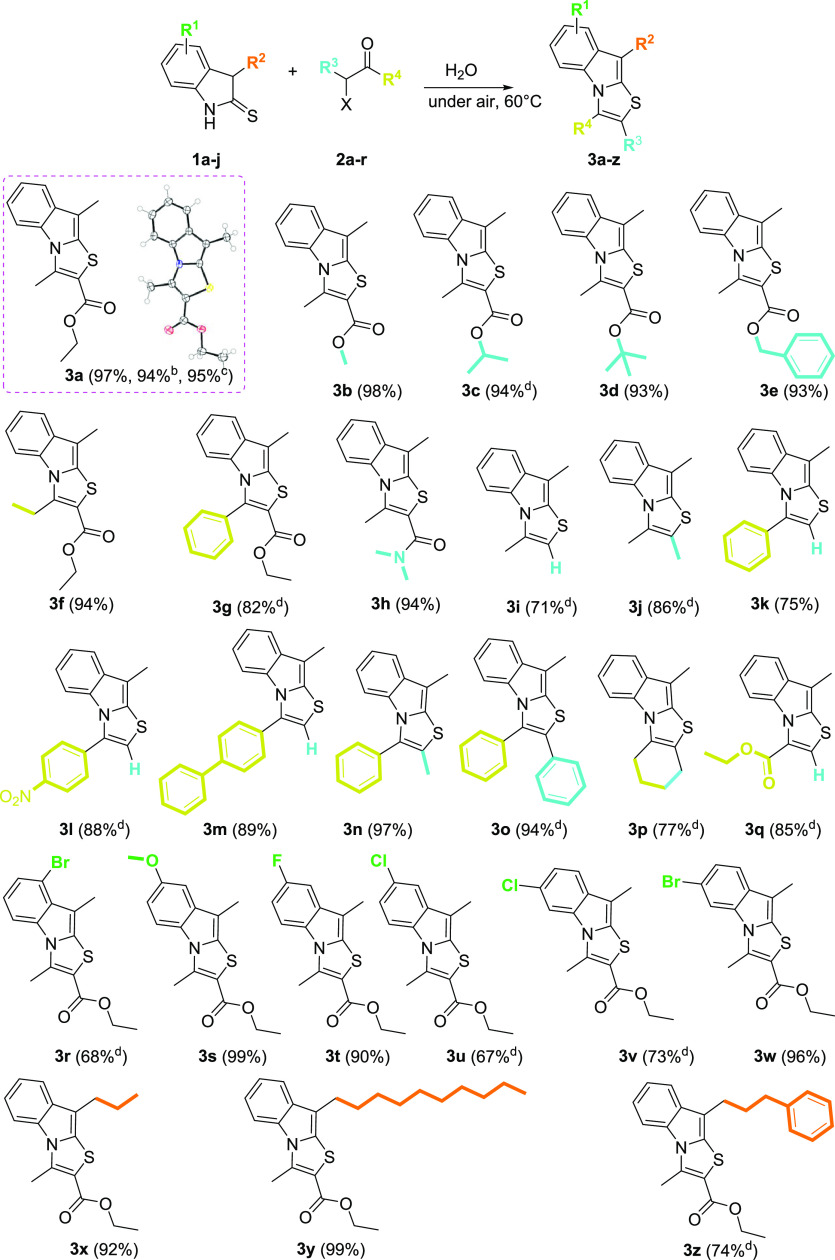
Substrate Scope for the Synthesis of Thiazolo[3,2-*a*]indoles **3**^a^

a Reaction conditions: **1** (0.7 mmol, 1 equiv), **2** (0.7 mmol, 1 equiv), in H_2_O (1.0 mL) at 60 °C, 5–14 h. The products **3** were obtained purely by extraction with ethyl acetate from the crude reaction (3 × 5 mL), unless otherwise stated.

b Ethyl 2-bromoacetoacetate **2a′** was employed.

c 4.00 mmol scale reaction (1.035 g).

d Isolated yields after purification on column chromatography of the extracted organic fraction.

To verify the robustness of the developed method, we next focused our attention on the substrate scope, examining initially the halogenated carbonyl partners **2**. As described in Table 2, the reaction is applicable to both α-halogenated-β-dicarbonyl and α-halogenated ketones, producing a wide substitution pattern in positions 2 and 3 of the resulting thiazolo[3,2-*a*]indoles **3a**–**p**. From alkyl chloro-ketoesters **2a**–**g** (Scheme S2) or chloro-ketoamide **2h**, several 2-carboxylated derivatives substituted in position 3 with alkyl (**3a–f,h**) or aryl moieties (**3g**) are easily obtained. On the other hand, the reaction with different α-halogenated ketones **2i**–**p** can produce heterocyclic systems whose 2 position is unsubstituted, alkylated, or arylated, while alkyl, aryl, and diphenyl groups can be introduced in position 3. Interestingly, the 2-chloro cyclohexanone **2p** was successfully employed, thus generating the intriguing fused tetracyclic 6,7,8,9-tetrahydrobenzo[4,5]thiazolo[3,2-*a*]indole **3p** in good yields, while the ethyl 3-bromo-2-oxopropanoate **2q** provided the corresponding ethyl 9-methylthiazolo[3,2-*a*]indole-3-carboxylate **3q**. In the latter case, it is noteworthy as it is likewise possible to smoothly insert the carboxylic function also in position 3 of the tricyclic structure. Successively, the behavior of 3-methylindoline-2-thiones **1b**–**g** differently substituted on the aromatic carbocyclic ring was investigated. In these cases, the commercial substituted indolin-2-ones **4b**–**g** (Table S3, Supporting Information) were methylated on C3 following the procedure reported by Kokatla^22^ and then sulfurylated according to the method described by Nivard.^21^

In this way, the corresponding 6-, 7-, 8-substituted thiazolo[3,2-*a*]indoles **1r**–**w** were achieved in good yields. Finally, according to the method reported by Yuan,^23^ starting from the indol-2-one **4a**, the 3-alkylindolin-2-ones **5h**–**j** were synthesized (please see Table S4, Supporting Information) and then converted into the corresponding 3-alkylindoline-2-thiones **1h**–**j**.^21^ Their reaction with ethyl 2-chloroacetoacetate **2a** produced the thiazolo[3,2-*a*]indoles **3x**–**z** substituted in position 9 with alkyl groups of different lengths. It is noteworthy that the scalability of the method was confirmed by repeating the synthesis of **3a** on a 4.00 mmol scale (95% yield, Table 2).

**Scheme 1:**
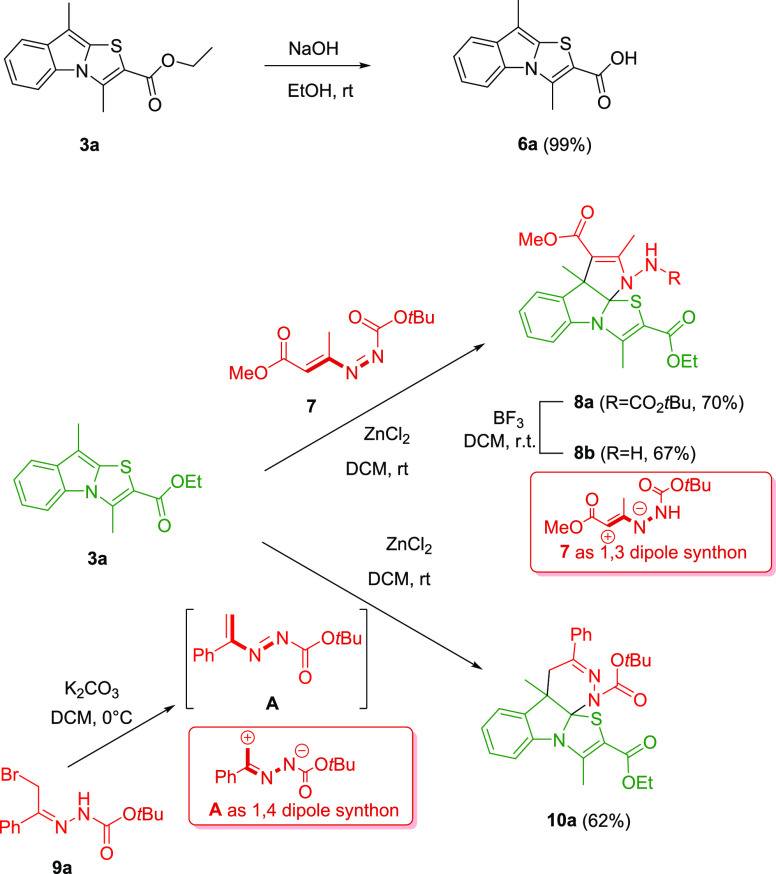
Examples of Thiazolo[3,2-*a*]indoles **3a** Derivatization

The single-crystal X-ray diffraction study of compound **3a** (Table 2)^24^ unequivocally confirms the suggested structure, thus validating the proposed retrosynthetic approach (Figure 3). To demonstrate the synthetic usefulness of the thiazolo[3,2-*a*]indoles **3**, some successive derivatizations have been carried out (Scheme 1). The ester group in position 2 of compound **3a** can be easily hydrolyzed by treatment with NaOH in ethanol at room temperature.^25^ Furthermore, considering the relevance and the properties of the functionalized polycyclic fused indoline frameworks,^26^ based on the previous literature,^27,28^ we planned to perform substrate-dependent divergent annulation reactions that involve excellent Michael acceptors such as the 1,2-diaza-1,3-dienes (DDs) **7,A**(29) as synthetic partners of the thiazolo[3,2-*a*]indoles **3a**. The zinc dichloride can efficiently catalyze both [3 + 2] and [4 + 2] dearomative annulations, in which DDs participate as C2N1 or C2N2 units (1,3 or 1,4 dipole synthons) providing the corresponding thiazolo-pyrroloindoline **8a** and thiazolo-pyridazinoindoline **10a**, respectively (Scheme 1).

The different pathway is attributable to the substitution of the azoene system (highlighted in bold in Scheme 1): in DD **7**, the alkoxycarbonyl group on the terminal carbon, making the geminal proton easily removable, favors the [3 + 2] cyclization,^27^ while the in situ generated DD **A**, lacking electron-withdrawing groups, acts as a 1,4-dipole synthon in a [4 + 2] annulation.^28^ It is noteworthy as in both cases, appealing derivatives **8a** and **10a**, characterized from a singular rigid tetracyclic skeleton containing an uncommon *N*,*N*,*S*-bridgehead quaternary carbon, are easily obtained. Furthermore, derivative **8a** was also treated with BF_3_ to deprotect the exocyclic amine function, generating the corresponding compound **8b**. The regioselectivity of the annulation process was confirmed by HMQC and HMBC analysis of the tetracyclic derivatives **8a,b** and **10a**.

## Conclusions

In conclusion, herein the first efficient and eco-friendly access to the thiazolo[3,2-*a*]indoles through a regioselective formal [3 + 2] cycloaddition of 3-substituted indoline-2-thiones and α-halogenated ketones is described. High yields, metal-free, easy product recovery, mild conditions, and use of an aqueous medium are the main features of this approach. The careful choice of the starting materials allows the simultaneous variation of up to four substituents located in six different positions in the final heterocyclic structure, permitting the easy creation of broad libraries of compounds. Furthermore, the indoline-2-thiones proved to be safe and effective sulfenylating reagents capable of obviating the classical poor reactivity of the C2-indole in the assembly of sulfurylated frameworks. Finally, the fruitful divergent assembly of rigid polycyclic skeletons characterized by uncommon *N*,*N*,*S*-bridgehead quaternary carbon represents an interesting unprecedent example of dearomative annulations.

## Experimental Section

### General Experimental Details

All the commercially available reagents and solvents were used without further purification. Chromatographic purification of compounds was carried out on silica gel (60–200 μm). TLC analysis was performed on preloaded (0.25 mm) glass-supported silica gel plates (Kieselgel 60); compounds were visualized by exposure to UV light and by dipping the plates in 1% Ce(SO_4_)·4H_2_O, 2.5% (NH_4_)_6_Mo_7_O_24_·4H_2_O in 10% sulfuric acid followed by heating on a hot plate. All ^1^H NMR and ^13^C NMR spectra were recorded at 400 and 100 MHz, respectively, using [D6]DMSO, CDCl_3_, or (CD_3_)_2_CO as a solvent. Chemical shifts (δ scale) are reported in parts per million (ppm) relative to the central peak of the solvent. The following abbreviations are used to describe peak patterns where appropriate: s = singlet, d = doublet, dd = doublet of doublet, dt = doublet of triplet, t = triplet, q = quartet, sept = septet, m = multiplet, and br = broad signal. All coupling constants (*J* value) are given in Hertz [Hz]. Structural assignments were made with additional information from gCOSY, gHSQC, and gHMBC experiments. High- and low-resolution mass spectroscopy was performed on a Micromass Q-ToF Micro mass spectrometer (Micromass, Manchester, UK) using an ESI source. Melting points were determined in open capillary tubes and are uncorrected.

### General Procedure for the Synthesis of Thiazol[3,2-*a*]indoles **3a–z**

To a solution of substituted 3-alkyl-indoline-2-thiones **1a**–**j** (0.7 mmol, 1.0 equiv) in water (1.0 mL), substituted α-halogenated carbonyl compounds **2a**–**r** (0.7 mmol, 1.0 equiv) were added, and the reaction mixtures were heated at 60 °C until the disappearance of the reagents (monitored by TLC, elution mixture cyclohexane: ethyl acetate, 95:5; 5.0–14.0 h). Then, the reaction mixtures were cooled at room temperature, and the crudes were saturated with sodium chloride and then extracted with ethyl acetate (3 × 5.0 mL). The solvent was then evaporated under reduced pressure, furnishing directly the desired thiazol[3,2-*a*]indoles **3**. Only derivatives **3c,g,i,j,l,o–r,u,v,z** were further purified by means of a chromatographic column (eluent mixture cyclohexane: ethyl acetate, 97:3).

### General Procedure for the Hydrolysis of Ethyl 3,9-Dimethylthiazolo[3,2-*a*]indole-2-carboxylate **3a** to 3,9-Dimethylthiazolo[3,2-*a*]indole-2-carboxylic Acid **6a**

To a solution of thiazol[3,2-*a*]indole **3a** (136.5 mg, 0.5 mmol, 1.0 equiv) in ethanol (2.0 mL), sodium hydroxide (200.0 mg, 5.0 mmol, 10 equiv) was added, and the reaction was stirred at room temperature. After the disappearance of **3a** (12.0 h, TLC check), the ethanol was removed under reduced pressure, and to the crude, ethyl acetate (10 mL) was added. The crude mixture was washed with an aqueous solution of sulfuric acid (0,1% v:v, 10 mL) until an acidic pH is reached. Then, the organic fraction was anhydrified with sodium sulfate, and the solvent was removed under reduced pressure, obtaining directly the pure 3,9-dimethylthiazolo[3,2-*a*]indole-2-carboxylic acid **6a**.

### General Procedure for the Formal [3 + 2] Cycloaddition Reactions of Thiazol[3,2-*a*]indoles **3a** with 1,2-Diaza-1,3-diene **7**

A mixture of thiazol[3,2-*a*]indole **3a** (82.0 mg, 0.3 mmol, 1.0 equiv), 1,2-diaza-1,3-diene **7** as the *E*/*Z* isomeric mixture (68.5 mg, 0.3 mmol, 1.0 equiv), and zinc dichloride (4.6 mg, 0.03 mmol, 0.1 equiv) was stirred in dry dichloromethane (2 mL) at room temperature. After the disappearance of the starting materials (3.0 h, TLC check), the crude mixture was purified by column chromatography on silica gel to afford 5-ethyl 1-methyl 3-((*tert*-butoxycarbonyl)amino)-2,6,11*b*-trimethyl-3,11*b*-dihydropyrrolo[2,3-*b*]thiazolo[3,2-*a*]indole-1,5-dicarboxylate **8a**.

### General Procedure for the Formal [4 + 2] Cycloaddition Reactions of Thiazol[3,2-*a*]indoles **3a** with In Situ Generated 1,2-Diaza-1,3-diene **A**

A mixture of thiazol[3,2-*a*]indole **3a** (82.0 mg, 0.3 mmol, 1.0 equiv), *tert*-butyl 2-(2-bromo-1-phenyletylidene)hydrazine-1-carboxylate **9a** (94.0 mg, 0.3 mmol, 1.0 equiv), potassium carbonate (165.8 mg, 1.2 mmol, 4.0 equiv), and zinc dichloride (4.6 mg, 0.03 mmol, 0.1 equiv) was stirred in dry dichloromethane (2 mL) at room temperature. After the disappearance of the starting materials (5.0 h, TLC check), the crude mixture was purified by column chromatography on silica gel to afford 4-(*tert*-butyl) 6-ethyl 7,12*b*-dimethyl-2-phenyl-1,12*b*-dihydro-4*H*-pyridazino[3,4-*b*]thiazolo[3,2-*a*]indole-4,6-dicarboxylate **10a**.

### General Procedure for the N-Deprotection of 5-Ethyl 1-Methyl 3-((*tert*-Butoxycarbonyl)amino)-2,6,11*b*-trimethyl-3,11*b*-dihydropyrrolo[2,3-*b*]thiazolo[3,2-*a*]indole-1,5-dicarboxylate **8a**

To a solution of **8a** (100.3 mg, 0.2 mmol, 1.0 equiv) in dichloromethane (5.0 mL), boron trifluoride diethyl etherate (29.6 μL, 0.24 mmol, 1.2 equiv) was added, and the reaction was stirred at room temperature. After the disappearance of **8a** (4.0 h, TLC check), the crude was washed with a saturated aqueous solution of sodium bicarbonate (2 × 4 mL). Then, the organic portion was anhydrified with sodium sulfate, the solvent was removed under reduced pressure, and the crude mixture was purified by column chromatography on silica gel to afford the pure 5-ethyl 1-methyl 3-amino-2,6,11*b*-trimethyl-3,11*b*-dihydropyrrolo[2,3-*b*]thiazolo[3,2-*a*]indole-1,5-dicarboxylate **8b**.

### Spectral Data

#### Ethyl 3,9-Dimethylthiazolo[3,2-*a*]indole-2-carboxylate **3a**

**3a** was isolated by ethyl acetate extraction from crude in 97% yield (186 mg). Yellowish solid; mp: 122–124 °C. The crystals analyzed via X-ray diffraction were obtained by dissolving **3a** in ethyl acetate and allowing it to evaporate at room temperature. ^1^H NMR (400 MHz, DMSO_*d*6_, 25 °C): δ = 7.95 (d, 1H, *J* = 7.6 Hz, Ar), 7.57 (d, 1H, *J* = 7.6 Hz, Ar), 7.28 (dt, 1H, *J* = 7.2 Hz, *J* = 0.8 Hz, Ar), 7.18 (dt, 1H, *J* = 7.2 Hz, *J* = 1.2 Hz, Ar), 4.28 (q, 2H, *J* = 7.2 Hz, O*CH*_*2*_CH_3_), 3.05 (s, 3H, CH_3_), 2.25 (s, 3H, CH_3_), 1.31 (t, 3H, *J* = 7.2 Hz, OCH_2_*CH*_*3*_); ^13^C{^1^H} NMR (100 MHz, DMSO_*d*6_, 25 °C): δ = 162.0, 142.0, 133.3, 132.0, 130.3, 122.2, 119.9, 117.6, 112.6, 108.6, 100.0, 61.0, 14.1, 13.6, 8.8; HRMS (ESI/Q-TOF) *m*/*z* [M + H]^+^ calcd for C_15_H_16_NO_2_S: 274.0896; found: 274.0911.

#### Methyl 3,9-Dimethylthiazolo[3,2-*a*]indole-2-carboxylate **3b**

**3b** was isolated by ethyl acetate extraction from crude in 98% yield (179 mg). Yellowish solid; mp: 132–134 °C; ^1^H NMR (400 MHz, DMSO_*d*6_, 25 °C): δ = 8.00 (d, 1H, *J* = 8.0 Hz, Ar), 7.60 (d, 1H, *J* = 8.0 Hz, Ar), 7.30 (dt, 1H, *J* = 7.2 Hz, *J* = 1.2 Hz, Ar), 7.20 (dt, 1H, *J* = 7.2 Hz, *J* = 1.2 Hz, Ar), 3.83 (s, 3H, OCH_3_), 3.11 (s, 3H, CH_3_), 2.29 (s, 3H, CH_3_); ^13^C{^1^H} NMR (100 MHz, DMSO_*d*6_, 25 °C): δ = 162.5, 142.3, 133.4, 132.0, 130.4, 122.2, 120.0, 117.7, 112.7, 108.3, 100.1, 52.2, 13.7, 8.9; HRMS (ESI/Q-TOF) *m*/*z* [M + H]^+^ calcd for C_14_H_14_NO_2_S: 260.0740; found: 260.0751.

#### Isopropyl 3,9-Dimethylthiazolo[3,2-*a*]indole-2-carboxylate **3c**

**3c** was isolated by column chromatography on silica gel (acetate/cyclohexane) in 94% yield (190 mg). Yellowish solid; mp: 115–117 °C; ^1^H NMR (400 MHz, CDCl_3_, 25 °C): δ = 7.84 (d, 1H, *J* = 8.1 Hz, Ar), 7.56 (d, 1H, *J* = 7.9 Hz, Ar), 7.31 (t, 1H, *J* = 7.6 Hz, Ar), 7.17 (t, 1H, *J* = 7.7 Hz, Ar), 5.24 (hept, 1H, *J* = 6.3 Hz, *CH*(CH_3_)_2_), 3.08 (s, 3H, CH_3_), 2.33 (s, 3H, CH_3_), 1.40 (d, 6H, *J* = 6.3 Hz, CH(*CH*_*3*_)_*2*_); ^13^C{^1^H} NMR (100 MHz, CDCl_3_, 25 °C): δ = 162.4, 141.2, 134.0, 133.3, 130.7, 122.0, 119.6, 117.7, 111.8, 110.4, 100.4, 68.8, 22.0, 13.9, 9.1; HRMS (ESI/Q-TOF) *m*/*z* [M + H]^+^ calcd for C_16_H_18_NO_2_S: 288.1053; found: 288.1058.

#### *tert*-Butyl 3,9-Dimethylthiazolo[3,2-*a*]indole-2-carboxylate **3d**

**3d** was isolated by ethyl acetate extraction from crude in 93% yield (194 mg). Yellowish solid; mp: 145–147 °C; ^1^H NMR (400 MHz, CDCl_3_) δ = 7.86 (dd, 1H, *J* = 8.3, *J* = 0.7 Hz, 1H), 7.57 (d, 1H, *J* = 8.0 Hz, Ar), 7.30 (ddd, 1H, *J* = 8.0 Hz, *J* = 7.2 Hz, *J* = 0.9 Hz, Ar), 7.17 (ddd, 1H, *J* = 8.3 Hz, *J* = 7.1 Hz, *J* = 1.2 Hz, Ar), 3.08 (s, 3H, CH_3_), 2.34 (s, 3H, CH_3_), 1.62 (s, 9H, C(CH_3_)_3_) ^13^C{^1^H} NMR (100 MHz, CDCl_3_) δ = 162.2, 140.6, 133.9, 133.3, 130.8, 121.9, 199.4, 117.7, 111.8, 111.5, 100.1, 82.2, 28.3, 13.8, 9.1; HRMS (ESI/Q-TOF) *m*/*z* [M + H]^+^ calcd for C_17_H_20_NO_2_S: 302.1209; found: 302.1203.

#### Benzyl 3,9-Dimethylthiazolo[3,2-*a*]indole-2-carboxylate **3e**

**3e** was isolated by ethyl acetate extraction from crude in 93% yield (218 mg). Yellowish solid; mp: 90–92 °C; ^1^H NMR (400 MHz, CDCl_3_) δ = 7.87 (d, 1H, *J* = 8.3 Hz, Ar), 7.57 (d, 1H, *J* = 8.0 Hz, Ar), 7.48–7.36 (m, 5H, Ar), 7.31 (ddd, 1H, *J* = 8.0 Hz, J = 7.2 Hz, *J* = 0.9 Hz, Ar), 7.19 (ddd, 1H, *J* = 8.3 Hz, *J* = 7.2 Hz, *J* = 1.2 Hz, Ar), 5.35 (s, 2H, CH_2_Ar), 3.12 (s, 3H, CH_3_), 2.33 (s, 3H, CH_3_); ^13^C{^1^H} NMR (100 MHz, CDCl_3_) δ = 162.6, 142.0, 135.8, 134.1, 133.2, 130.8, 128.3, 128.0, 122.2, 119.7, 117.8, 111.9, 109.5, 100.6, 66.6, 14.1, 9.1; HRMS (ESI/Q-TOF) *m*/*z* [M + H]^+^ calcd for C_20_H_18_NO_2_S: 336.1053; found: 336.1050.

#### Ethyl 3-Ethyl-9-methylthiazolo[3,2-*a*]indole-2-carboxylate **3f**

**3f** was isolated by ethyl acetate extraction from crude in 94% yield (188 mg). Yellowish solid; mp: 105–107 °C; ^1^H NMR (400 MHz, CDCl_3_) δ = 7.79 (d, 1H, *J* = 8.3 Hz, Ar), 7.58 (d, 1H, *J* = 8.0 Hz, Ar), 7.32 (t, 1H, *J* = 7.6 Hz, Ar), 7.21 (ddd, 1H, *J* = 8.3 Hz, *J* = 7.1 Hz, *J* = 1.2 Hz, Ar), 4.37 (q, 2H, *J* = 7.1 Hz, O*CH*_*2*_CH_3_), 3.62 (q, 2H, *J* = 7.5 Hz, *CH*_*2*_CH_3_), 2.35 (s, 3H, CH_2_*CH*_*3*_), 1.47 (t, 3H, *J* = 7.5 Hz, OCH_2_*CH*_*3*_), 1.41 (t, 3H, *J* = 7.1 Hz, CH_2_*CH*_*3*_); ^13^C{^1^H} NMR (100 MHz, CDCl_3_) δ = 162.6, 147.1, 134.1, 133.6, 130.2, 122.0, 119.8, 117.8, 112.0, 109.4, 100.6, 61.1, 20.3, 14.3, 12.0, 9.2; HRMS (ESI/Q-TOF) *m*/*z* [M + H]^+^ calcd for C_16_H_18_NO_2_S: 288.1053; found: 288.1071.

#### Ethyl 9-Methyl-3-phenylthiazolo[3,2-*a*]indole-2-carboxylate **3g**

**3g** was isolated by column chromatography on silica gel (acetate/cyclohexane) in 82% yield (190 mg). Yellowish solid; mp: 95–97 °C; ^1^H NMR (400 MHz, CDCl_3_) δ = 7.66–7.58 (m, 3H, Ar), 7.57–7.51 (m, 3H, Ar), 7.21 (ddd, 1H, *J* = 8.0 Hz, *J* = 7.2 Hz, *J* = 0.9 Hz, Ar), 6.85 (ddd, 1H, *J* = 8.3 Hz, *J* = 7.1 Hz, *J* = 1.2 Hz, Ar), 6.46 (d, 1H, *J* = 8.4 Hz, Ar), 4.18 (q, 2H, *J* = 7.1 Hz, O*CH*_*2*_CH_3_), 2.39 (s, 3H, CH_3_), 1.16 (t, *J* = 7.1 Hz, 3H, OCH_2_*CH*_*3*_); ^13^C{^1^H} NMR (100 MHz, CDCl_3_) δ = 161.9, 141.6, 133.8, 133.2, 130.5, 130.1, 129.9, 129.5, 128.8, 122.1, 119.5, 117.6, 112.6, 111.9, 100.9, 61.0, 13.9, 9.2; HRMS (ESI/Q-TOF) *m*/*z* [M + H]^+^ calcd for C_20_H_18_NO_2_S: 336.1053; found: 336.1044.

#### *N*,*N*,3,9-Tetramethylthiazolo[3,2-*a*]indole-2-carboxamide **3h**

**3h** was isolated by ethyl acetate extraction from crude in 94% yield (179 mg). Yellowish solid; mp: 132–134 °C; ^1^H NMR (400 MHz, CDCl_3_, 25 °C): δ = 7.81 (d, 1H, *J* = 8.0 Hz, Ar), 7.58 (d, 1H, *J* = 8.0 Hz, Ar), 7.27 (dt, 1H, *J* = 7.2 Hz, *J* = 0.8 Hz, Ar), 7.16 (dt, 1H, *J* = 7.2 Hz, *J* = 1.2 Hz, Ar), 3.14 (s, 6H, N(CH_3_)_2_), 2.78 (s, 3H, CH_3_), 2.36 (s, 3H, CH_3_); ^13^C{^1^H} NMR (100 MHz, CDCl_3_, 25 °C): δ = 164.2, 133.2, 132.9, 132.7, 130.4, 121.1, 119.2, 117.7, 112.3, 111.1, 99.8, 37.6, 14.2, 9.2; HRMS (ESI/Q-TOF) *m*/*z* [M + H]^+^ calcd for C_15_H_17_N_2_OS: 273.1056; found: 273.1059.

### 3,9-Dimethylthiazolo[3,2-*a*]indole **3i**

**3i** was isolated by column chromatography on silica gel (acetate/cyclohexane) in 71% yield (100 mg). Yellowish solid; mp: 80–82 °C; ^1^H NMR (400 MHz, CDCl_3_, 25 °C): δ = 7.82 (d, 1H, *J* = 8.0 Hz, Ar), 7.59 (d, 1H, *J* = 8.0 Hz, Ar), 7.26 (dt, 1H, *J* = 8.0 Hz, *J* = 0.8 Hz, Ar), 7.15 (dt, 1H, *J* = 6.8 Hz, *J* = 1.2 Hz, Ar), 6.16 (brs, 1H, Ar), 2.72 (d, 3H, *J* = 1.2 Hz, CH_3_), 2.38 (s, 3H, CH_3_); ^13^C{^1^H} NMR (100 MHz, CDCl_3_, 25 °C): δ = 132.8, 131.7, 130.3, 120.6, 118.9, 117.7, 110.8, 103.5, 100.0, 97.9, 15.0, 9.5; HRMS (ESI/Q-TOF) *m*/*z* [M + H]^+^ calcd for C_12_H_12_NS: 202.0685; found: 202.0670.

### 2,3,9-Trimethylthiazolo[3,2-*a*]indole **3j**

**3j** was isolated by column chromatography on silica gel (acetate/cyclohexane) in 86% yield (131 mg). Yellowish solid; mp: 98–100 °C; ^1^H NMR (400 MHz, CDCl_3_) δ = 7.81 (dd, 1H, *J* = 8.3, *J* = 0.7 Hz, Ar), 7.56 (d, 1H, *J* = 7.5 Hz, Ar), 7.21 (t, 1H, *J* = 7.5 Hz, Ar), 7.11 (t, 1H, *J* = 7.7 Hz, Ar), 2.63 (s, 3H, CH_3_), 2.36 (s, 3H, CH_3_), 2.33 (s, 3H, CH_3_); ^13^C{^1^H} NMR (100 MHz, CDCl_3_) δ = 133.6, 132.0, 130.32, 126.1, 119.9, 118.4, 117.5, 113.9, 110.6, 98.5, 12.9, 12.6, 9.4; HRMS (ESI/Q-TOF) *m*/*z* [M + H]^+^ calcd for C_13_H_14_NS: 216.0841; found: 216.0847.

### 9-Methyl-3-phenylthiazolo[3,2-*a*]indole **3k**

**3k** was isolated by ethyl acetate extraction from crude in 75% yield (138 mg). Yellow oil; ^1^H NMR (400 MHz, CDCl_3_, 25 °C): δ = 7.63–7.65 (m, 2H, Ar), 7.53–7.59 (m, 4H, Ar), 7.19 (dt, 1H, *J* = 8.0 Hz, *J* = 0.8 Hz, Ar), 7.02 (d, 1H, *J* = 8.4 Hz, Ar), 6.91 (dt, 1H, *J* = 8.0 Hz, *J* = 0.8 Hz, Ar), 6.35 (s, 1H, Ar), 2.42 (s, 3H, CH_3_); ^13^C{^1^H} NMR (100 MHz, CDCl_3_, 25 °C): δ = 135.6, 135.6, 132.8, 130.8, 129.9, 129.6, 129.2, 128.7, 120.4, 118.4, 117.5, 111.6, 106.1, 9.3; HRMS (ESI/Q-TOF) *m*/*z* [M + H]^+^ calcd for C_17_H_14_NS: 264.0841; found: 264.0848.

### 9-Methyl-3-(4-nitrophenyl)thiazolo[3,2-*a*]indole **3l**

**3l** was isolated by column chromatography on silica gel (acetate/cyclohexane) in 88% yield (189 mg). Pale red solid; mp: 158–160 °C; ^1^H NMR (400 MHz, CDCl_3_, 25 °C): δ = 8.41 (d, 2H, *J* = 8.8 Hz, Ar), 7.84 (d, 2H, *J* = 8.8 Hz, Ar), 7.62 (d, 1H, *J* = 8.4 Hz, Ar), 7.24 (dt, 1H, *J* = 8.0 Hz, *J* = 1.2 Hz, Ar), 6.96–7.05 (m, 2H, Ar), 6.51 (s, 1H, Ar), 2.43 (s, 3H, CH_3_); ^13^C{^1^H} NMR (100 MHz, CDCl_3_, 25 °C): δ = 148.3, 137.0, 135.3, 133.4, 132.9, 129.6, 124.1, 120.9, 119.0, 118.0, 111.4, 109.2, 100.7, 9.2; HRMS (ESI/Q-TOF) *m*/*z* [M + H]^+^ calcd for C_17_H_13_N_2_O_2_S: 309.0692; found: 309.0675.

### 3-([1,1′-Biphenyl]-4-yl)-9-methylthiazolo[3,2-*a*]indole **3m**

**3m** was isolated by ethyl acetate extraction from crude in 89% yield (211 mg). Yellowish solid; mp: 138–140 °C; ^1^H NMR (400 MHz, CDCl_3_, 25 °C): δ = 7.78 (d, 2H, *J* = 8.1 Hz, Ar), 7.69–7.75 (m, 4H, Ar), 7.60 (d, 1H, *J* = 8.0 Hz, Ar), 7.52 (t, 2H, *J* = 7.6 Hz, Ar), 7.43 (t, 1H, *J* = 7.2 Hz, Ar), 7.21 (t, 1H, *J* = 7.6 Hz, Ar), 7.16 (d, 1H, *J* = 8.4 Hz, Ar), 6.95 (t, 1H, *J* = 8.0 Hz, Ar), 6.45 (s, 1H, Ar), 2.42 (s, 3H, CH_3_); ^13^C{^1^H} NMR (100 MHz, CDCl_3_, 25 °C): δ = 142.4, 140.2, 135.5, 133.0, 130.1, 129.7, 129.6, 129.5, 129.0, 127.8, 127.4, 127.2, 127.1, 120.7, 118.7, 117.7, 111.8, 106.7, 9.5; HRMS (ESI/Q-TOF) *m*/*z* [M + H]^+^ calcd for C_23_H_18_NS: 340.1154; found: 340.1162.

### 2,9-Dimethyl-3-phenylthiazolo[3,2-*a*]indole **3n**

**3n** was isolated by ethyl acetate extraction from crude in 97% yield (189 mg). Yellowish solid; mp: 98–100 °C; ^1^H NMR (400 MHz, CDCl_3_, 25 °C): δ = 7.51–7.59 (m, 6H, Ar), 7.14 (dt, 1H, *J* = 6.8 Hz, J = 0.8 Hz, Ar), 6.84 (dt, 1H, *J* = 7.2 Hz, *J* = 1.2 Hz, Ar), 6.67 (d, 1H, *J* = 7.6 Hz, Ar), 2.42 (s, 3H, CH_3_), 2.25 (s, 3H, CH_3_); ^13^C{^1^H} NMR (100 MHz, CDCl_3_, 25 °C): δ = 133.9, 132.0, 130.5, 130.2, 130.1, 130.0, 129.4, 128.9, 120.0, 118.4, 117.8, 117.4, 111.1, 98.5, 13.3, 9.4; HRMS (ESI/Q-TOF) *m*/*z* [M + H]^+^ calcd for C_18_H_16_NS: 278.0998; found: 278.1012.

### 9-Methyl-2,3-diphenylthiazolo[3,2-*a*]indole **3o**

**3o** was isolated by column chromatography on silica gel (acetate/cyclohexane) in 94% yield (221 mg). Yellowish solid; mp: 150–152 °C; ^1^H NMR (400 MHz, CDCl_3_, 25 °C): δ = 7.55–7.62 (m, 6H, Ar), 7.15–7.27 (m, 6H, Ar), 6.85 (t, 1H, *J* = 7.2 Hz, Ar), 6.55 (d, 1H, *J* = 8.4 Hz, Ar), 2.46 (s, 3H, CH_3_); ^13^C{^1^H} NMR (100 MHz, CDCl_3_, 25 °C): δ = 132.9, 132.6, 132.4, 130.8, 130.4, 130.0, 129.8, 129.3, 128.4, 128.2, 127.3, 121.5, 120.6, 118.6, 117.4, 111.4, 99.6, 9.3; HRMS (ESI/Q-TOF) *m*/*z* [M + H]^+^ calcd for C_23_H_18_NS: 340.1154; found: 340.1167.

### 11-Methyl-6,7,8,9-tetrahydrobenzo[4,5]thiazolo[3,2-*a*]indole **3p**

**3p** was isolated by column chromatography on silica gel (acetate/cyclohexane) in 77% yield (131 mg). Yellowish solid; mp: 75–77 °C; ^1^H NMR (400 MHz, CDCl_3_) δ = 7.71 (d, 1H, *J* = 8.2 Hz, Ar), 7.57 (dd, 1H, *J* = 8.0 Hz, *J* = 0.7 Hz, Ar), 7.17–7.23 (m, 1H, Ar),7.09 (ddd, 1H, *J* = 8.2 Hz, *J* = 7.1 Hz, *J* = 1.2 Hz, Ar), 3.06–3.10 (m, 2H, Cycloalk.), 2.66–2.70 (m, 2H, Cycloalk.), 2.38 (s, 3H, CH_3_), 1.97–2.05 (m, 2H, Cycloalk.), 1.97–1.89 (m, 2H, Cycloalk.); ^13^C{^1^H} NMR (100 MHz, CDCl_3_) δ = 133.9, 131.9, 130.0, 128.8, 119.8, 118.5, 117.5, 117.0, 110.7, 98.8, 24.8, 24.2, 22.9, 22.1, 9.4; HRMS (ESI/Q-TOF) *m*/*z* [M + H]^+^ calcd for C_15_H_16_NS: 242.0998; found: 242.0981.

### Ethyl 9-Methylthiazolo[3,2-*a*]indole-3-carboxylate **3q**

**3q** was isolated by column chromatography on silica gel (acetate/cyclohexane) in 85% yield (154 mg). Yellowish oil; ^1^H NMR (400 MHz, CDCl_3_, 25 °C): δ = 8.64 (dd, 1H, *J* = 8.8 Hz, *J* = 0.8 Hz, Ar), 7.56–7.60 (m, 1H, Ar), 7.55 (s, 1H, Ar), 7.27–7.31(m, 1H, Ar), 720–7.24 (m, 1H, Ar), 4.47 (q, 2H, *J* = 7.2 Hz, O*CH*_*2*_CH_3_), 2.40 (s, 3H, CH_3_), 1.46 (t, 3H, *J* = 7.2 Hz, OCH_2_*CH*_*3*_); ^13^C{^1^H} NMR (100 MHz, CDCl_3_, 25 °C): δ = 158.6, 134.3, 132.5, 130.9, 128.2, 121.4, 121.0, 119.6, 117.2, 114.8, 101.1, 61.7, 14.3, 9.2; HRMS (ESI/Q-TOF) *m*/*z* [M + H]^+^ calcd for C_14_H_14_NO_2_S: 260.0740; found: 260.0755.

### Ethyl 8-Bromo-3,9-dimethylthiazolo[3,2-*a*]indole-2-carboxylate **3r**

**3r** was isolated by column chromatography on silica gel (acetate/cyclohexane) in 68% yield (165 mg). Yellowish solid; mp: 70–72 °C; ^1^H NMR (400 MHz, DMSO_*d*6_, 25 °C): δ = 8.03 (d, 1H, *J* = 8.4 Hz, Ar), 7.47 (d, 1H, *J* = 7.6 Hz, Ar), 7.07 (d, 1H, *J* = 8.0 Hz, Ar), 4.31 (q, 2H, *J* = 7.2 Hz, O*CH*_*2*_CH_3_), 3.09 (s, 3H, CH_3_), 2.51 (s, 3H, CH_3_), 1.32 (t, 3H, *J* = 7.2 Hz, OCH_2_*CH*_*3*_); ^13^C{^1^H} NMR (100 MHz, CDCl_3_, 25 °C): δ = 162.6, 141.0, 135.6, 131.9, 131.2, 130.3, 126.6, 120.2, 113.1, 111.1, 101.5, 61.3, 14.3, 14.2, 12.4; HRMS (ESI/Q-TOF) *m*/*z* [M + H]^+^ calcd for C_15_H_15_BrNO_2_S: 352.0001; found: 352.0017.

### Ethyl 7-Methoxy-3,9-dimethylthiazolo[3,2-*a*]indole-2-carboxylate **3s**

**3s** was isolated by ethyl acetate extraction from crude in 99% yield (211 mg). White solid; mp: 96–98 °C; ^1^H NMR (400 MHz, CDCl_3_, 25 °C): δ = 7.74 (d, 1H, *J* = 9.2 Hz, Ar), 6.98 (d, 1H, *J* = 2.4 Hz, Ar), 6.80 (dd, 1H, *J* = 8.8 Hz, *J* = 2.4 Hz, Ar), 4.36 (q, 2H, *J* = 7.2 Hz, O*CH*_*2*_CH_3_), 3.91 (s, 3H, OCH_3_), 3.08 (s, 3H, CH_3_), 2.30 (s, 3H, CH_3_), 1.40 (t, 3H, *J* = 7.2 Hz, OCH_2_*CH*_*3*_); ^13^C{^1^H} NMR (100 MHz, CDCl_3_, 25 °C): δ = 163.0, 155.7, 141.2, 135.0, 134.1, 125.89, 112.6, 109.2, 109.0, 100.3, 99.8, 61.0, 55.7, 14.4, 13.8, 9.2; HRMS (ESI/Q-TOF) *m*/*z* [M + H]^+^ calcd for C_16_H_18_NO_3_S: 304.1002; found: 304.0985.

### Ethyl 7-Fluoro-3,9-dimethylthiazolo[3,2-*a*]indole-2-carboxylate **3t**

**3t** was isolated by ethyl acetate extraction from crude in 90% yield (181 mg). Yellowish solid; mp: 125–127 °C; ^1^H NMR (400 MHz, CDCl_3_, 25 °C): δ = 7.77 (dd, 1H, *J* = 9.2 Hz, *J* = 4.0 Hz, Ar), 7.19 (dd, 1H, *J* = 9.2 Hz, *J* = 2.4 Hz, Ar), 6.90 (dt, 1H, *J* = 8.8 Hz, *J* = 2.4 Hz, Ar), 4.36 (q, 2H, *J* = 7.2 Hz, O*CH*_*2*_CH3), 3.08 (s, 3H, CH_3_), 2.29 (s, 3H, CH_3_), 1.40 (t, 3H, *J* = 7.2 Hz, OCH_2_*CH*_*3*_); ^13^C{^1^H} NMR (100 MHz, CDCl_3_, 25 °C): δ = 162.8, 159.1 (*J*_C–F_ = 237.6 Hz), 141.1, 135.2, 134.9 (*J*_C–F_ = 9.9 Hz), 127.3, 112.6 (*J*_C–F_ = 9.8 Hz), 110.0, 107.6 (*J*_C–F_ = 26.0 Hz), 103.0 (*J*_C–F_ = 23.6 Hz), 100.5 (*J*_C–F_ = 4.3 Hz), 61.2, 13.9, 9.2; HRMS (ESI/Q-TOF) *m*/*z* [M + H]^+^ calcd for C_15_H_15_FNO_2_S: 292.0802; found: 292.0821.

### Ethyl 7-Chloro-3,9-dimethylthiazolo[3,2-*a*]indole-2-carboxylate **3u**

**3u** was isolated by column chromatography on silica gel (acetate/cyclohexane) in 67% yield (146 mg). Yellowish solid; mp: 135–137 °C; ^1^H NMR (400 MHz, CDCl_3_, 25 °C): δ = 7.76 (dd, 1H, *J* = 8.8 Hz, *J* = 0.4 Hz, Ar), 7.52 (dd, 1H, *J* = 2.0 Hz, J = 0.4 Hz, Ar), 7.12 (dd, 1H, *J* = 8.8 Hz, *J* = 2.0 Hz, Ar), 4.37 (q, 2H, *J* = 7.2 Hz, O*CH*_*2*_CH_3_), 3.09 (s, 3H, CH_3_), 2.30 (s, 3H, CH_3_), 1.40 (t, 3H, *J* = 7.2 Hz, OCH_2_*CH*_*3*_); ^13^C{^1^H} NMR (100 MHz, CDCl_3_, 25 °C): δ = 162.7, 141.0, 135.0, 134.9, 129.0, 128.0, 119.7, 117.3, 112.7, 110.7, 100.2, 61.3, 14.3, 13.9, 9.1; HRMS (ESI/Q-TOF) *m*/*z* [M + H]^+^ calcd for C_15_H_15_ClNO_2_S: 308.0507; found: 308.0530.

### Ethyl 6-Chloro-3,9-dimethylthiazolo[3,2-*a*]indole-2-carboxylate **3v**

**3v** was isolated by column chromatography on silica gel (acetate/cyclohexane) in 73% yield (153 mg). Yellowish solid; mp: 83–85 °C; ^1^H NMR (400 MHz, DMSO_*d*6_, 25 °C): δ = 8.04 (d, 1H, *J* = 1.6 Hz, Ar), 7.63 (d, 1H, *J* = 8.4 Hz, Ar), 7.33 (dd, 1H, *J* = 8.4 Hz, *J* = 1.6 Hz, Ar), 4.32 (q, 2H, *J* = 7.2 Hz, O*CH*_*2*_CH_3_), 3.12 (s, 3H, CH_3_), 2.30 (s, 3H, CH_3_), 1.32 (t, 3H, *J* = 7.2 Hz, OCH_2_*CH*_*3*_); ^13^C{^1^H} NMR (100 MHz, CDCl_3_, 25 °C): δ = 162.6, 140.9, 133.9, 132.7, 131.1, 125.2, 118.8, 114.8, 112,9, 100.9, 61.3, 14.3, 13.9, 9.1; HRMS (ESI/Q-TOF) *m*/*z* [M + H]^+^ calcd for C_15_H_15_ClNO_2_S: 308.0507; found: 308.0514.

### Ethyl 6-Bromo-3,9-dimethylthiazolo[3,2-*a*]indole-2-carboxylate **3w**

**3w** was isolated by ethyl acetate extraction from crude in 96% yield (237 mg). Yellow oil; ^1^H NMR (400 MHz, DMSO_*d*6_, 25 °C): δ = 8.29 (d, 1H, *J* = 1.2 Hz, Ar), 7.72 (d, 1H, *J* = 8.8 Hz, Ar), 7.59 (dd, 1H, *J* = 8.4 Hz, *J* = 1.6 Hz, Ar), 4.46 (q, 2H, *J* = 7.2 Hz, O*CH*_*2*_CH_3_), 3.25 (s, 3H, CH_3_), 2.43 (s, 3H, CH_3_), 1.46 (t, 3H, *J* = 7.2 Hz, OCH_2_*CH*_*3*_); ^13^C{^1^H} NMR (100 MHz, CDCl_3_, 25 °C): δ = 162.6, 140.9, 133.9, 132.7, 131.1, 125.2, 118.8, 114.8, 112.9, 110.9, 100.6, 61.3, 14.3, 13.9, 9.1; HRMS (ESI/Q-TOF) *m*/*z* [M + H]^+^ calcd for C_15_H_15_BrNO_2_S: 352.0001; found: 352.0010.

### Ethyl 3-Methyl-9-propylthiazolo[3,2-*a*]indole-2-carboxylate **3x**

**3x** was isolated by ethyl acetate extraction from crude in 92% yield (190 mg). Yellowish oil; ^1^H NMR (400 MHz, CDCl_3_, 25 °C): δ = 7.89 (d, 1H, *J* = 8.0 Hz, Ar), 7.60 (d, 1H, *J* = 8.0 Hz, Ar), 7.30 (dt, 1H, *J* = 8.0 Hz, *J* = 0.8 Hz, Ar), 7.18 (dt, 1H, *J* = 7.2 Hz, *J* = 1.2 Hz, Ar), 4.36 (q, 2H, *J* = 7.2 Hz, O*CH*_*2*_CH_3_), 3.14 (s, 3H, CH_3_), 2.78 (dt, 2H, *J* = 7.6 Hz, *CH*_*2*_CH_2_CH_3_), 1.80 (sex, 2H, *J* = 7.2 Hz, CH_2_*CH*_*2*_CH_3_), 1.41 (t, 3H, *J* = 7.2 Hz, OCH_2_*CH*_*3*_), 1.04 (t, 3H, *J* = 7.6 Hz, CH_2_CH_2_*CH*_*3*_); ^13^C{^1^H} NMR (100 MHz, CDCl_3_, 25 °C): δ = 162.9, 141.4, 133.7, 133.0, 130.8, 122.0, 119.6, 118.0, 112.0, 109.9, 105.5, 61.1, 26.7, 22.1, 14.4, 14.2, 14.0; HRMS (ESI/Q-TOF) *m*/*z* [M + H]^+^ calcd for C_17_H_20_NO_2_S: 302.1209; found: 302.1201.

### Ethyl 9-Decyl-3-methylthiazolo[3,2-*a*]indole-2-carboxylate **3y**

**3y** was isolated by ethyl acetate extraction from crude in 99% yield (273 mg). Yellowish solid; mp: 55–57 °C; ^1^H NMR (400 MHz, CDCl_3_, 25 °C): δ = 7.89 (d, 1H, *J* = 8.4 Hz, *J* = 0.8 Hz, Ar), 7.60 (dd, 1H, *J* = 8.0 Hz, *J* = 0.8 Hz, Ar), 7.30 (dt, 1H, *J* = 8.0 Hz, *J* = 0.8 Hz, Ar), 7.18 (dt, 1H, *J* = 7.2 Hz, *J* = 0.8 Hz, Ar), 4.37 (q, 2H, *J* = 7.2 Hz, O*CH*_*2*_CH_3_), 3.14 (s, 3H, CH_3_), 2.79 (t, 2H, *J* = 7.6 Hz, decyl), 1.76 (quint., 2H, *J* = 6.8 Hz, decyl), 1.41 (t, 3H, *J* = 7.2 Hz, OCH_2_*CH*_*3*_), 1.23–1.34 (m, 12H, decyl), 0.86–0.92 (m, 5H, decyl); ^13^C{^1^H} NMR (100 MHz, CDCl_3_, 25 °C): δ = 162.9, 141.4, 133.6, 132.9, 130.8, 122.0, 119.6, 118.0, 112.0, 109.9, 105.8, 61.1, 31.9, 29.6, 29.5, 29.3, 28.7, 24.6, 22.7, 14.4, 14.1, 14.0; HRMS (ESI/Q-TOF) *m*/*z* [M + H]^+^ calcd for C_24_H_34_NO_2_S: 400.2305; found: 400.2298.

### Ethyl 3-Methyl-9-(3-phenylpropyl)thiazolo[3,2-*a*]indole-2-carboxylate **3z**

**3z** was isolated by column chromatography on silica gel (acetate/cyclohexane) in 74% yield (192 mg). Yellow oil; ^1^H NMR (400 MHz, CDCl_3_, 25 °C): δ = 7.90 (dd, 1H, *J* = 8.0 Hz, *J* = 0.8 Hz, Ar), 7.57 (dt, 1H, *J* = 7.6 Hz, *J* = 0.8 Hz, Ar), 7.28–7.32 (m, 3H, Ar), 7.17–7.21 (m, 4H, Ar), 4.37 (q, 2H, *J* = 7.2 Hz, O*CH*_*2*_CH_3_), 3.15 (s, 3H, CH_3_), 2.86 (t, 2H, *J* = 7.2 Hz, *CH*_*2*_CH_2_CH_2_Ph), 2.71 (t, 2H, *J* = 7.6 Hz, CH_2_CH_2_*CH*_*2*_Ph), 2.12 (quint, 2H, *J* = 7.2 Hz, CH_2_*CH*_*2*_CH_2_Ph), 1.41 (t, 3H, *J* = 7.2 Hz, OCH_2_*CH*_*3*_); ^13^C{^1^H} NMR (100 MHz, CDCl_3_, 25 °C): δ = 162.9, 142.1, 141.4, 133.6, 133.1, 130.9, 128.4, 128.3, 125.7, 122.1, 119.7, 117.9, 112.0, 110.0, 105.1, 61.1, 35.7, 30.2, 24.1, 14.4, 14.1, 14.0; HRMS (ESI/Q-TOF) *m*/*z* [M + H]^+^ calcd for C_23_H_24_NO_2_S: 378.1522; found: 378.1504.

### 3,9-Dimethylthiazolo[3,2-*a*]indole-2-carboxylic Acid **6a**

**6a** was isolated by column chromatography on siica gel (acetate/cyclohexane) in 99% yield (118 mg). Yellowish solid; mp: 215–217 °C; ^1^H NMR (400 MHz, (CD_3_)_2_CO, 25 °C): δ = 11.49 (brs, 1H, OH), 8.00 (d, 1H, *J* = 8.4 Hz, Ar), 7.59 (d, 1H, *J* = 7.6 Hz, Ar), 7.29 (dt, 1H, *J* = 7.2 Hz, *J* = 0.8 Hz, Ar), 7.19 (dt, 1H, *J* = 7.2 Hz, *J* = 1.2 Hz, Ar), 3.16 (s, 3H, CH_3_), 2.32 (s, 3H, CH_3_); ^13^C{^1^H} NMR (100 MHz, (CD_3_)_2_CO, 25 °C): δ = 162.9, 141.8, 133.8, 130.9,132.8, 122.0, 119.7, 117.5, 112.2, 109.8, 99.9, 13.1, 8.1; HRMS (ESI/Q-TOF) *m*/*z* [M + H]^+^ calcd for C_13_H_12_NO_2_S: 246.0583; found: 246.0567.

### 5-Ethyl 1-Methyl 3-((*tert*-Butoxycarbonyl)amino)-2,6,11*b*-trimethyl-3,11*b*-dihydropyrrolo[2,3-*b*]thiazolo[3,2-*a*]indole-1,5-dicarboxylate **8a**

**8a** was isolated by column chromatography on silica gel (acetate/cyclohexane) in 70% yield (101 mg). Yellowish solid; mp: 93–95 °C. Notably, compound **8a** in NMR analysis shows two sets of peaks. This fact is probably ascribable to the presence of a second axis along the N–N bond that determines the existence of syn/anti-rotamers of carbamates.^30^^1^H NMR (400 MHz, DMSO_*d*6_, 25 °C): δ = 8.96, 9.10, 9.40, 9.46 (4brs, 1H, NH), 7.60–7.65 (m, 1H, Ar), 7.28–7.37 (m, 1H, Ar), 7.19–7.24 (m, 1H, Ar), 7.03–7.08 (m, 1H, Ar), 4.05–4.18 (m, 2H, O*CH*_*2*_CH_3_), 3.71 (s, 3H, OCH_3_), 2.59, 2.65 (2brs, 3H, CH_3_), 1.98, 2.02 (2brs, 3H, CH_3_), 1.60, 1.62 (2brs, 3H, CH_3_), 1.37, 1.39, 1.42 (3brs, 9H, C(CH_3_)_3_), 1.17–1.24 (m, 3H, OCH_2_*CH*_*3*_); ^13^C{^1^H} NMR (100 MHz, DMSO_*d*6_, 25 °C): δ = 165.9, 163.0, 158.6, 157.8, 155.7, 154.7, 143.9, 143.6, 141.2, 141.0, 140.1, 128.1, 128.0, 125.0, 124.9, 124.1, 123.6, 114.0, 113.9, 113.4, 113.2, 111.9, 101.8, 101.3, 101.3, 99.7, 98.2, 80.5, 80.0, 79.7, 59.8, 59.8, 55.8, 55.1, 50.4, 50.3, 27.9, 27.8, 27.6, 23.8, 23.8, 14.7, 14.3, 14.2, 12.7, 11.8; HRMS (ESI/Q-TOF) *m*/*z* [M + H]^+^ calcd for C_25_H_32_N_3_O_6_S: 502.2006; found: 502.1987.

### 5-Ethyl 1-Methyl 3-Amino-2,6,11*b*-trimethyl-3,11*b*-dihydropyrrolo[2,3-*b*]thiazolo[3,2-*a*]indole-1,5-dicarboxylate **8b**

**8b** was isolated by column chromatography on silica gel (acetate/cyclohexane) in 67% yield (54 mg). Yellowish oil. °C; ^1^H NMR (400 MHz, DMSO_*d*6_, 25 °C): δ = 7.59 (dd, 1H, *J* = 7.6 Hz, *J* = 0.8 Hz, Ar), 7.33 (d, 1H, *J* = 7.6 Hz, Ar), 7.22 (dt, 1H, *J* = 8.0 Hz, *J* = 1.2 Hz, Ar), 7.06 (dt, 1H, *J* = 7.6 Hz, *J* = 0.8 Hz, Ar), 4.50 (s, 2H, NH_2_), 4.11–4.16 (m, 2H, O*CH*_*2*_CH_3_), 3.67 (s, 3H, OCH_3_), 2.67 (s, 3H, CH_3_), 2.13 (s, 3H, CH_3_), 1.58 (s, 3H, CH_3_), 1.22 (t, 3H, *J* = 7.2 Hz, OCH_2_*CH*_*3*_); ^13^C{^1^H} NMR (100 MHz, DMSO_*d*6_, 25 °C): δ = 166.2, 163.1, 160.1, 144.9, 141.2, 140.4, 127.9, 125.1, 124.3, 114.1, 112.8, 99.1, 98.1, 59.9, 55.4, 50.0, 24.2, 14.5, 14.3, 12.6; HRMS (ESI/Q-TOF) *m*/*z* [M + H]^+^ calcd for C_20_H_24_N_3_O_4_S: 402.1482; found: 402.1495.

### 4-(*tert*-Butyl) 6-Ethyl 7,12*b*-Dimethyl-2-phenyl-1,12*b*-dihydro-4*H*-pyridazino[3,4-*b*]thiazolo[3,2-*a*]indole-4,6-dicarboxylate **10a**

**10a** was isolated by column chromatography on silica gel (acetate/cyclohexane) in 62% yield (92 mg). Yellowish solid; mp: 104–106 °C; ^1^H NMR (400 MHz, DMSO_*d*6_, 25 °C): δ = 7.77–7.80 (m, 2H, Ar), 7.37–7.48 (m, 3H, Ar), 7.33 (d, 1H, *J* = 8.0 Hz, Ar), 7.26 (d, 1H, *J* = 7.6 Hz, Ar), 7.21 (t, 1H, *J* = 8.0 Hz, Ar), 7.02 (t, 1H, *J* = 7.2 Hz, Ar), 4.13–4.18 (m, 2H, O*CH*_*2*_CH_3_), 3.70 (d, 1H, *J* = 18.0 Hz, CH_2_), 2.74 (d, 1H, *J* = 18.0 Hz, CH_2_), 2.54 (s, 3H, CH_3_), 1.43 (s, 9H, C(CH_3_)_3_), 1.41 (s, 3H, CH_3_), 1.22 (t, 3H, *J* = 7.2 Hz, OCH_2_*CH*_*3*_); ^13^C{^1^H} NMR (100 MHz, DMSO_*d*6_, 25 °C): δ = 163.0, 151.6, 145.0, 144.4, 140.8, 138.3, 136.3, 129.2, 128.4, 128.4, 125.4, 124.8, 122.4, 116.1, 101.3, 98.3, 81.4, 59.8, 49.9, 28.1, 27.7, 14.4, 13.5; HRMS (ESI/Q-TOF) *m*/*z* [M + H]^+^ calcd for C_28_H_32_N_3_O_4_S: 506.2108; found: 506.2122.

## Data Availability

The data underlying this study are available in the published article and its Supporting Information.

## Supporting Information Available

General experimental details, description of the starting materials, general procedures, spectral data of compounds **3a–z,6a,8a,b,10a**, NMR spectra of compounds **3a–z,6a,8a,b,10a**, X-ray structure analysis of compounds **3a** (CCDC-2295941), and the Green metrics: First pass Metric Toolkit (PDF).
