# Supplementary material for: Useful Access to Uncommon Thiazolo[3,2-a]indoles
Source: J Org Chem. 2024 Jan 9;89(2):1184–92. doi: 10.1021/acs.joc.3c02338 (PMC10804410; doi:10.1021/acs.joc.3c02338)
Supplement: Supplementary file 1 — jo3c02338_si_001.pdf [file jo3c02338_si_001.pdf]

Useful access to uncommon thiazol[3,2-*a*]indoles

Giacomo Mari,*[a] Lucia De Crescentini,[a] Gianfranco Favi,[a] Amalija Golobič,[b] Stefania Santeusanio[a] and Fabio Mantellini*[a]

[a] Dr. G. Mari, Dr. L. De Crescentini, Prof. G. Favi, Dr. S. Santeusanio, Prof. F. Mantellini
Department of Biomolecular Sciences, Section of Chemistry and Pharmaceutical Technologies,
University of Urbino "Carlo Bo",
Via I Maggetti 24, 6109, Urbino (PU), Italy.

[b] Prof. A. Golobič,
Faculty of Chemistry and Chemical Technology,
University of Ljubljana,
Večna pot 113,
1000 Ljubljana,
Slovenia

e-mails: giacomo.mari@uniurb.it; fabio.mantellini@uniurb.it

Table of Contents

1 General experimental details. 2

2 Starting Materials

2.1 Table S1: Substituted 3-alkyl-indoline-2-thiones 1a–j employed. 3

2.2 Table S2: Substituted α-halogenated carbonyl compounds 2a–r employed. 3

2.3 Table S3: Substituted indoline-2-ones 4a–g. 4

2.4 Table S4: Substituted 3-alkyl-indoline-2-ones 5a–j 4

3 General procedures

3.1 General procedure for the synthesis of thiazol[3,2-*a*]indoles 3a–z. 5

3.2 General procedure for the hydrolysis of ethyl 3,9-dimethylthiazolo[3,2-*a*]indole-2-carboxylate 3a to 3,9-dimethylthiazolo[3,2-*a*]indole-2-carboxylic acid 6a. 5

3.3 General procedure for the formal [3+2] cycloaddition reactions of thiazol[3,2-*a*]indoles 3a with 1,2-diaza-1,3-diene 7. 5

3.4 General procedure for the formal [4+2] cycloaddition reactions of thiazol[3,2-*a*]indoles 3a with in situ generated 1,2-diaza-1,3-diene A. 5

3.5 General procedure for the N-deprotection of 5-Ethyl 1-methyl 3-((tert-butoxycarbonyl)amino)-2,6,11b-trimethyl-3,11b-dihydropyrrolo[2,3-*b*]thiazolo[3,2-*a*]indole-1,5-dicarboxylate 8a. 6

4 Spectral data

4.1 Spectral data of compounds 3a–z. 7

4.2 Spectral data of compounds 6a, 8a,b, 10a. 13

5 NMR spectra

5.1 NMR spectra of compounds 3a–z. 15

5.2 NMR spectra of compounds 6a, 8a,b. 46

6 X-ray structure analysis of compounds 3a (CCDC-2295941) 53

6.1 Figure S1. ORTEP diagram of molecule of compound 3a with ellipsoids at 50 % probability level (CCDC-2295941). 54

6.2. Table S5. Crystal data and structure refinement for 3a. 55

7 Green metrics: First pass Metric Toolkit. 56

8 References 57

1 General experimental details.

All the commercially available reagents and solvents were used without further purification. Chromatographic purification of compounds was carried out on silica gel (60–200 μm). TLC analysis was performed on pre-loaded (0.25 mm) glass supported silica gel plates (Kieselgel 60); compounds were visualized by exposure to UV light and by dipping the plates in 1% $Ce(SO_4)\cdot 4H_2O$, 2.5% $(NH_4)_6Mo_7O_{24}\cdot 4H_2O$ in 10% sulphuric acid followed by heating on a hot plate. All ^{1}H NMR and ^{13}C NMR spectra were recorded at 400 and 100 MHz, respectively, using [D_6]DMSO, $CDCl_3$ or $(CD_3)_2CO$ as solvent. Chemical shift (δ scale) are reported in parts per million (ppm) relative to the central peak of the solvent and are sorted in ascending order within each group. The following abbreviations are used to describe peak patterns where appropriate: s = singlet, d = doublet, dd = doublet of doublet, dt = doublet of triplet, t = triplet, q = quartet, sept = septet, m = multiplet and br = broad signal. All coupling constants (J value) are given in Hertz [Hz]. Structural assignments were made with additional information from gCOSY, gHSQC, and gHMBC experiments. High-and low-resolution mass spectroscopy was performed on a Micromass Q-ToF Micro mass spectrometer (Micromass, Manchester, UK) using an ESI source. Melting points were determined in open capillary tubes and are uncorrected.

2 Starting materials

2.1 Table S1: Substituted 3-alkyl-indoline-2-thiones 1a–j employed.[1]

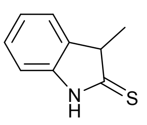

1a

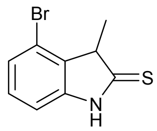

1b

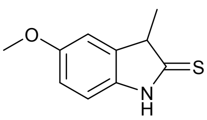

1c

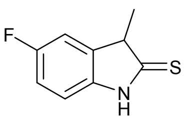

1d

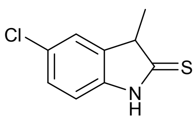

1e

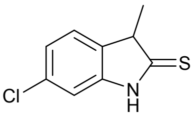

1f

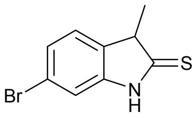

1g

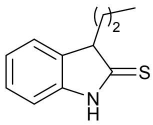

1h

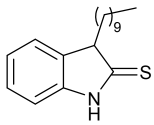

1i

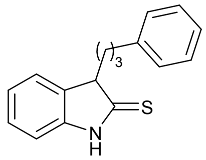

1j

2.2 Table S2: Substituted α-halogenated carbonyl compounds 2a–r employed.

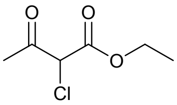

2a

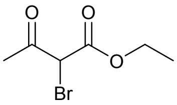

2a'

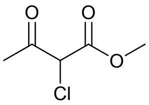

2b

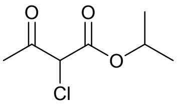

2c

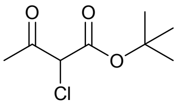

2d

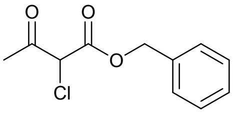

2e

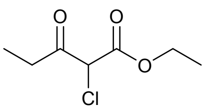

2f

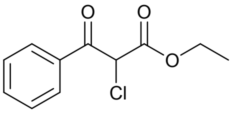

2g

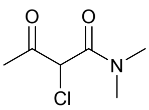

2h

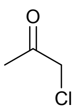

2i

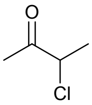

2j

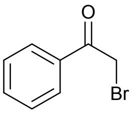

2k

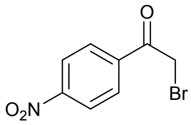

2l

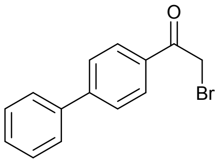

2m

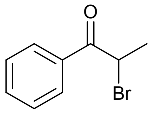

2n

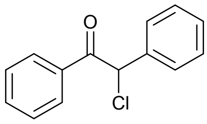

2o

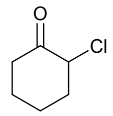

2p

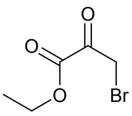

2q

2.3 Table S3: Substituted indoline-2-ones 4a–g employed as precursors of the substituted 3-alkyl-indoline-2-ones 5a–j.

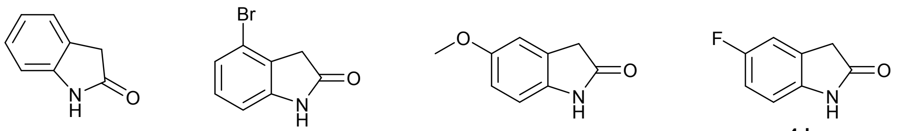

4a **4b** **4c** **4d**

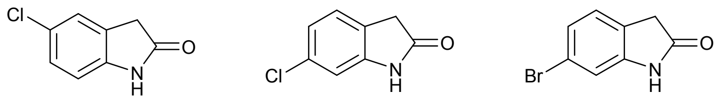

4e **4f** **4g**

2.4 Table S4: Substituted 3-alkyl-indoline-2-ones 5a–j employed as precursors of the substituted 3-alkyl-indoline-2-thiones 1a–j.

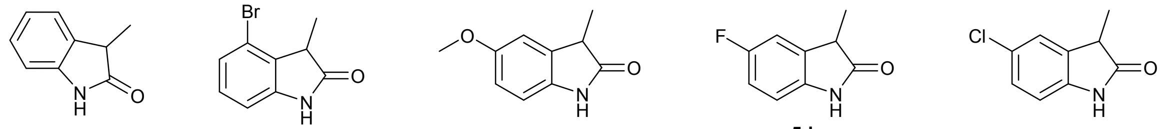

5a **5b** **5c** **5d** **5e**

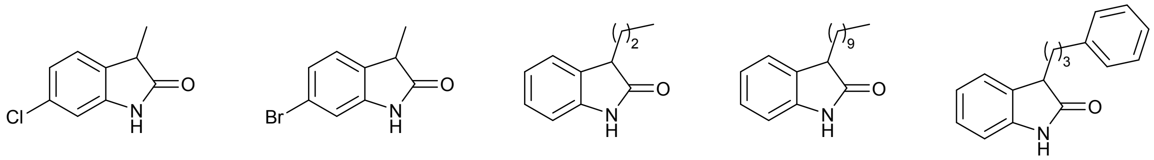

5f **5g** **5h** **5i** **5j**

3 General procedures

3.1 General procedure for the synthesis of thiazol[3,2-*a*]indoles 3a–z.

To a solution of substituted 3-alkyl-indoline-2-thiones **1a–j** (0.7 mmol, 1.0 equiv.) in water (1.0 ml), substituted α-halogenated carbonyl compounds **2a–r** (0.7 mmol, 1.0 equiv.) were added and the reaction mixtures were heated at 60°C until the disappearance of the reagents (monitored by TLC, elution mixture cyclohexane: ethyl acetate, 95 : 5; 5.0–14.0 h). Then the reaction mixtures were cooled at room temperature, the crudes were saturated with sodium chloride, and then extracted with ethyl-acetate (3 x 5.0 mL). The solvent was then evaporated under reduced pressure furnishing directly the desired thiazol[3,2-*a*]indoles **3**. Only derivatives **3c,g,i,j,l,o-r,u,v,z** were further purified by means of a chromatographic column (eluent mixture cyclohexane: ethyl acetate, 97 : 3).

3.2 General procedure for the hydrolysis of ethyl 3,9-dimethylthiazolo[3,2-*a*]indole-2-carboxylate 3a to 3,9-dimethylthiazolo[3,2-*a*]indole-2-carboxylic acid 6a.

To a solution of thiazol[3,2-*a*]indole **3a** (136.5 mg, 0.5 mmol, 1.0 equiv.) in ethanol (2.0 mL), sodium hydroxide (200.0 mg, 5.0 mmol, 10 equiv.) was added and the reaction was stirred at room temperature. After the disappearance of **3a** (12.0 h, TLC check), the ethanol was removed under reduced pression, and to the crude ethyl acetate (10 mL) was added. The crude mixture was washed with an aqueous solution of sulfuric acid (0,1% v:v, 10 mL) until an acidic pH is reached. Then, the organic fraction was anhydrified with sodium sulphate, the solvent removed under reduced pressure, obtaining directly the pure 3,9-dimethylthiazolo[3,2-*a*]indole-2-carboxylic acid **6a**.

3.3 General procedure for the formal [3+2] cycloaddition reactions of thiazol[3,2-*a*]indoles 3a with 1,2-diaza-1,3-diene 7.

A mixture of thiazol[3,2-*a*]indole **3a** (82.0 mg, 0.3 mmol, 1.0 equiv.), 1,2-diaza-1,3-diene **7** as *E*/*Z* isomeric mixture (68.5 mg, 0.3 mmol, 1.0 equiv.) and zinc dichloride (4.6 mg, 0.03 mmol, 0.1 equiv.) was stirred in dry dichloromethane (2 mL) at room temperature. After the disappearance of the starting materials (3.0 h, TLC check), the crude mixture was purified by column chromatography on silica gel to afford 5-ethyl 1-methyl 3-

((*tert*-butoxycarbonyl)amino)-2,6,11*b*-trimethyl-3,11*b*-dihydropyrrolo[2,3-*b*]thiazolo[3,2-*a*]indole-1,5-dicarboxylate **8a**.

3.4 General procedure for the formal [4+2] cycloaddition reactions of thiazol[3,2-*a*]indoles 3a with in situ generated 1,2-diaza-1,3-diene A.

A mixture of thiazol[3,2-*a*]indole **3a** (82.0 mg, 0.3 mmol, 1.0 equiv.), *tert*-butyl 2-(2-bromo-1-phenyletylidene)hydrazine-1-carboxylate **9a** (94.0 mg, 0.3 mmol, 1.0 equiv.), potassium carbonate (165.8 mg, 1.2 mmol, 4.0 equiv.), and zinc dichloride (4.6 mg, 0.03 mmol, 0.1 equiv.) was stirred in dry dichloromethane (2 mL) at room temperature. After the disappearance of the starting materials (5.0 h, TLC check), the crude mixture was purified by column chromatography on silica gel to afford 4-(*tert*-butyl) 6-ethyl 7,12*b*-dimethyl-2-phenyl-1,12*b*-dihydro-4*H*-pyridazino[3,4-*b*]thiazolo[3,2-*a*]indole-4,6-dicarboxylate **10a**.

3.5 General procedure for the N-deprotection of 5-Ethyl 1-methyl 3-((tert-butoxycarbonyl)amino)-2,6,11b-trimethyl-3,11b-dihydropyrrolo[2,3-*b*]thiazolo[3,2-*a*]indole-1,5-dicarboxylate 8a.

To a solution of **8a** (100.3 mg, 0.2 mmol, 1.0 equiv.) in dichloromethane (5.0 mL), boron trifluoride diethyl etherate (29.6 μl, 0.24 mmol, 1.2 equiv.) was added and the reaction was stirred at room temperature. After the disappearance of **8a** (4.0 h, TLC check), the crude was washed with a saturated aqueous solution of sodium bicarbonate (2 x 4mL).Then, the organic portion was anhydrified with sodium sulfate, the solvent removed under reduced pressure, the crude mixture was purified by column chromatography on silica gel to afford the pure 5-ethyl 1-methyl 3-amino-2,6,11*b*-trimethyl-3,11*b*-dihydropyrrolo[2,3-*b*]thiazolo[3,2-*a*]indole-1,5-dicarboxylate **8b**.

4 Spectral data

4.1 Spectral data of compounds 3a–z.

Ethyl 3,9-dimethylthiazolo[3,2-*a*]indole-2-carboxylate 3a.

3a was isolated by ethyl acetate extraction from crude in 97% yield (186 mg). Yellowish solid; mp: 122–124 °C; ^{1}H NMR (400 MHz, DMSO$_{d6}$, 25 °C): δ = 7.95 (d, 1H, *J* = 7.6 Hz, Ar), 7.57 (d, 1H, *J* = 7.6 Hz, Ar), 7.28 (dt, 1H, *J* = 7.2 Hz, *J* = 0.8 Hz, Ar), 7.18 (dt, 1H, *J* = 7.2 Hz, *J* = 1.2 Hz, Ar), 4.28 (q, 2H, *J* = 7.2 Hz, O*CH$_2$*CH$_3$), 3.05 (s, 3H, CH$_3$), 2.25 (s, 3H, CH$_3$), 1.31 (t, 3H, *J* = 7.2 Hz, OCH$_2$*CH$_3$*); ^{13}C{^{1}H} NMR (100 MHz, DMSO$_{d6}$, 25 °C): δ = 162.0, 142.0, 133.3, 132.0, 130.3, 122.2, 119.9, 117.6, 112.6, 108.6, 100.0, 61.0, 14.1, 13.6, 8.8; HRMS (ESI/Q-TOF) m/z [M + H]$^+$ calcd for $C_{15}H_{16}NO_2S$: 274.0896; found: 274.0911.

Methyl 3,9-dimethylthiazolo[3,2-*a*]indole-2-carboxylate 3b.

3b was isolated by ethyl acetate extraction from crude in 98% yield (179 mg). Yellowish solid; mp: 132–134 °C; ^{1}H NMR (400 MHz, DMSO$_{d6}$, 25 °C): δ = 8.00 (d, 1H, *J* = 8.0 Hz, Ar), 7.60 (d, 1H, *J* = 8.0 Hz, Ar), 7.30 (dt, 1H, *J* = 7.2 Hz, *J* = 1.2 Hz, Ar), 7.20 (dt, 1H, *J* = 7.2 Hz, *J* = 1.2 Hz, Ar), 3.83 (s, 3H, OCH$_3$), 3.11 (s, 3H, CH$_3$), 2.29 (s, 3H, CH$_3$); ^{13}C{^{1}H} NMR (100 MHz, DMSO$_{d6}$, 25 °C): δ = 162.5, 142.3, 133.4, 132.0, 130.4, 122.2, 120.0, 117.7, 112.7, 108.3, 100.1, 52.2, 13.7, 8.9; HRMS (ESI/Q-TOF) m/z [M + H]$^+$ calcd for $C_{14}H_{14}NO_2S$: 260.0740; found: 260.0751.

Isopropyl 3,9-dimethylthiazolo [3,2-*a*]indole-2-carboxylate 3c.

3c was isolated by column chromatography on silica gel (acetate/cyclohexane) in 94% yield (190 mg). Yellowish solid; mp: 115–117 °C; ^{1}H NMR (400 MHz, CDCl$_3$, 25 °C): δ = 7.84 (d, 1H, *J* = 8.1 Hz, Ar), 7.56 (d, 1H, *J* = 7.9 Hz, Ar), 7.31 (t, 1H, *J* = 7.6 Hz, Ar), 7.17 (t, 1H, *J* = 7.7 Hz, Ar), 5.24 (hept, 1H, *J* = 6.3 Hz, *CH*(CH$_3$)$_2$), 3.08 (s, 3H, CH$_3$), 2.33 (s, 3H, CH$_3$), 1.40 (d, 6H, *J* = 6.3 Hz, CH(*CH$_3$)$_2$*); ^{13}C{^{1}H} NMR (100 MHz, CDCl$_3$, 25 °C): δ = 162.4, 141.2, 134.0, 133.3, 130.7, 122.0, 119.6, 117.7, 111.8, 110.4, 100.4, 68.8, 22.0, 13.9, 9.1; HRMS (ESI/Q-TOF) m/z [M + H]$^+$ calcd for $C_{16}H_{18}NO_2S$: 288.1053; found: 288.1058.

***Tert*-butyl 3,9-dimethylthiazolo[3,2-*a*]indole-2-carboxylate 3d.**

3d was isolated by ethyl acetate extraction from crude in 93% yield (194 mg). Yellowish solid; mp: 145–147 °C; ^{1}H NMR (400 MHz, CDCl$_3$) δ = 7.86 (dd, 1H, *J* = 8.3, *J* = 0.7 Hz, 1H), 7.57 (d, 1H, *J* = 8.0 Hz, Ar), 7.30 (ddd, 1H, *J* = 8.0 Hz, *J* = 7.2 Hz, *J* = 0.9 Hz, Ar), 7.17 (ddd, 1H, *J* = 8.3 Hz, *J* = 7.1 Hz, *J* = 1.2 Hz, Ar), 3.08 (s, 3H, CH$_3$), 2.34 (s, 3H, CH$_3$), 1.62 (s, 9H, C(CH$_3$)$_3$) ^{13}C{^{1}H} NMR (100 MHz, CDCl$_3$) δ =.162.2, 140.6, 133.9, 133.3, 130.8, 121.9, 199.4, 117.7, 111.8, 111.5, 100.1, 82.2, 28.3, 13.8, 9.1; HRMS (ESI/Q-TOF) m/z [M + H]$^+$ calcd for $C_{17}H_{20}NO_2S$: 302.1209; found: 302.1203.

Benzyl 3,9-dimethylthiazolo[3,2-*a*]indole-2-carboxylate 3e.

3e was isolated by ethyl acetate extraction from crude in 93% yield (218 mg). Yellowish solid; mp: 90–92 °C; ^{1}H NMR (400 MHz, CDCl$_3$) δ = 7.87 (d, 1H, *J* = 8.3 Hz, Ar), 7.57 (d, 1H, *J* = 8.0 Hz, Ar), 7.48–7.36 (m, 5H, Ar), 7.31 (ddd, 1H, *J* = 8.0 Hz, J = 7.2 Hz, *J* = 0.9 Hz, Ar), 7.19 (ddd, 1H, *J* = 8.3 Hz, *J* = 7.2 Hz, *J* = 1.2 Hz, Ar), 5.35 (s, 2H, CH$_2$Ar), 3.12 (s, 3H, CH$_3$), 2.33 (s, 3H, CH$_3$); ^{13}C{^{1}H} NMR (100 MHz, CDCl$_3$) δ = 162.6, 142.0, 135.8, 134.1, 133.2, 130.8, 128.3, 128.0, 122.2, 119.7, 117.8, 111.9, 109.5, 100.6, 66.6, 14.1, 9.1; HRMS (ESI/Q-TOF) m/z [M + H]$^+$ calcd for $C_{20}H_{18}NO_2S$: 336.1053; found: 336.1050.

Ethyl 3-ethyl-9-methylthiazolo[3,2-*a*]indole-2-carboxylate 3f.

3f was isolated by ethyl acetate extraction from crude in 94% yield (188 mg). Yellowish solid; mp: 105–107 °C; ^{1}H NMR (400 MHz, $CDCl_3$) δ = 7.79 (d, 1H, *J* = 8.3 Hz, Ar), 7.58 (d, 1H, *J* = 8.0 Hz, Ar), 7.32 (t, 1H, *J* = 7.6 Hz, Ar), 7.21 (ddd, 1H, *J* = 8.3 Hz, *J* = 7.1 Hz, *J* = 1.2 Hz, Ar), 4.37 (q, 2H, *J* = 7.1 Hz, O$CH_2$$CH_3$), 3.62 (q, 2H, *J* = 7.5 Hz, $CH_2$$CH_3$), 2.35 (s, 3H, $CH_2$$CH_3$), 1.47 (t, 3H, *J* = 7.5 Hz, O$CH_2$$CH_3$), 1.41 (t, 3H, *J* = 7.1 Hz, $CH_2$$CH_3$); ^{13}C{^{1}H} NMR (100 MHz, $CDCl_3$) δ = 162.6, 147.1, 134.1, 133.6, 130.2, 122.0, 119.8, 117.8, 112.0, 109.4, 100.6, 61.1, 20.3, 14.3, 12.0, 9.2; HRMS (ESI/Q-TOF) m/z [M + H]$^+$ calcd for $C_{16}H_{18}NO_2S$: 288.1053; found: 288.1071.

Ethyl 9-methyl-3-phenylthiazolo[3,2-*a*]indole-2-carboxylate 3g.

3g was isolated by column chromatography on silica gel (acetate/cyclohexane) in 82% yield (190 mg). Yellowish solid; mp: 95–97 °C; ^{1}H NMR (400 MHz, $CDCl_3$) δ = 7.66–7.58 (m, 3H, Ar), 7.57–7.51 (m, 3H, Ar), 7.21 (ddd, 1H, *J* = 8.0 Hz, *J* = 7.2 Hz, *J* = 0.9 Hz, Ar), 6.85 (ddd, 1H, *J* = 8.3 Hz, *J* = 7.1 Hz, *J* = 1.2 Hz, Ar), 6.46 (d, 1H, *J* = 8.4 Hz, Ar), 4.18 (q, 2H, *J* = 7.1 Hz, O$CH_2$$CH_3$), 2.39 (s, 3H, CH_3), 1.16 (t, *J* = 7.1 Hz, 3H, O$CH_2$$CH_3$); ^{13}C{^{1}H} NMR (100 MHz, $CDCl_3$) δ = 161.9, 141.6, 133.8, 133.2, 130.5, 130.1, 129.9, 129.5, 128.8, 122.1, 119.5, 117.6, 112.6, 111.9, 100.9, 61.0, 13.9, 9.2; HRMS (ESI/Q-TOF) m/z [M + H]$^+$ calcd for $C_{20}H_{18}NO_2S$: 336.1053; found: 336.1044.

***N*,*N*,3,9-tetramethylthiazolo[3,2-*a*]indole-2-carboxamide 3h.**

3h was isolated by ethyl acetate extraction from crude in 94% yield (179 mg). Yellowish solid; mp: 132–134 °C; ^{1}H NMR (400 MHz, $CDCl_3$, 25 °C): δ = 7.81 (d, 1H, *J* = 8.0 Hz, Ar), 7.58 (d, 1H, *J* = 8.0 Hz, Ar), 7.27 (dt, 1H, *J* = 7.2 Hz, *J* = 0.8 Hz, Ar), 7.16 (dt, 1H, *J* = 7.2 Hz, *J* = 1.2 Hz, Ar), 3.14 (s, 6H, $N(CH_3)_2$), 2.78 (s, 3H, CH_3), 2.36 (s, 3H, CH_3); ^{13}C{^{1}H} NMR (100 MHz, $CDCl_3$, 25 °C): δ = 164.2, 133.2, 132.9, 132.7, 130.4, 121.1, 119.2, 117.7, 112.3, 111.1, 99.8, 37.6, 14.2, 9.2; HRMS (ESI/Q-TOF) m/z [M + H]$^+$ calcd for $C_{15}H_{17}N_2OS$: 273.1056; found: 273.1059.

3,9-Dimethylthiazolo[3,2-*a*]indole 3i.

3i was isolated by column chromatography on silica gel (acetate/cyclohexane) in 71% yield (100 mg). Yellowish solid; mp: 80–82 °C; ^{1}H NMR (400 MHz, $CDCl_3$, 25 °C): δ = 7.82 (d, 1H, *J* = 8.0 Hz, Ar), 7.59 (d, 1H, *J* = 8.0 Hz, Ar), 7.26 (dt, 1H, *J* = 8.0 Hz, *J* = 0.8 Hz, Ar), 7.15 (dt, 1H, *J* = 6.8 Hz, *J* = 1.2 Hz, Ar), 6.16 (brs, 1H, Ar), 2.72 (d, 3H, *J* = 1.2 Hz, CH_3), 2.38 (s, 3H, CH_3); ^{13}C{^{1}H} NMR (100 MHz, $CDCl_3$, 25 °C): δ = 132.8, 131.7, 130.3, 120.6, 118.9, 117.7, 110.8, 103.5, 100.0, 97.9, 15.0, 9.5; HRMS (ESI/Q-TOF) m/z [M + H]$^+$ calcd for $C_{12}H_{12}NS$: 202.0685; found: 202.0670.

2,3,9-Trimethylthiazolo[3,2-*a*]indole 3j.

3j was isolated by column chromatography on silica gel (acetate/cyclohexane) in 86% yield (131 mg). Yellowish solid; mp: 98–100 °C; ^{1}H NMR (400 MHz, $CDCl_3$) δ = 7.81 (dd, 1H, *J* = 8.3, *J* = 0.7 Hz, Ar), 7.56 (d, 1H, *J* = 7.5 Hz, Ar), 7.21 (t, 1H, *J* = 7.5 Hz, Ar), 7.11 (t, 1H, *J* = 7.7 Hz, Ar), 2.63 (s, 3H, CH_3), 2.36 (s, 3H, CH_3), 2.33 (s, 3H, CH_3); ^{13}C{^{1}H} NMR (100 MHz, $CDCl_3$) δ = 133.6, 132.0, 130.32, 126.1, 119.9, 118.4, 117.5, 113.9, 110.6, 98.5, 12.9, 12.6, 9.4; HRMS (ESI/Q-TOF) m/z [M + H]$^+$ calcd for $C_{13}H_{14}NS$: 216.0841; found: 216.0847.

9-Methyl-3-phenylthiazolo[3,2-*a*]indole 3k.

3k was isolated by ethyl acetate extraction from crude in 75% yield (138 mg). Yellow oil; ^{1}H NMR (400 MHz, $CDCl_3$, 25 °C): δ = 7.63–7.65 (m, 2H, Ar), 7.53–7.59 (m, 4H, Ar), 7.19 (dt, 1H, *J* = 8.0 Hz, *J* = 0.8 Hz, Ar), 7.02 (d, 1H, *J* = 8.4 Hz, Ar), 6.91 (dt, 1H, *J* = 8.0 Hz, *J* = 0.8 Hz, Ar), 6.35 (s, 1H, Ar), 2.42 (s, 3H, CH_3); $^{13}C\{^1H\}$ NMR (100 MHz, $CDCl_3$, 25 °C): δ = 135.6, 135.6, 132.8, 130.8, 129.9, 129.6, 129.2, 128.7, 120.4, 118.4, 117.5, 111.6, 106.1, 9.3; HRMS (ESI/Q-TOF) m/z $[M + H]^+$ calcd for $C_{17}H_{14}NS$: 264.0841; found: 264.0848.

9-Methyl-3-(4-nitrophenyl)thiazolo[3,2-*a*]indole 3l.

3l was isolated by column chromatography on silica gel (acetate/cyclohexane) in 88% yield (189 mg). Pale red solid; mp: 158–160 °C; ^{1}H NMR (400 MHz, $CDCl_3$, 25 °C): δ = 8.41 (d, 2H, *J* = 8.8 Hz, Ar), 7.84 (d, 2H, *J* = 8.8 Hz, Ar), 7.62 (d, 1H, *J* = 8.4 Hz, Ar), 7.24 (dt, 1H, *J* = 8.0 Hz, *J* = 1.2 Hz, Ar), 6.96–7.05 (m, 2H, Ar), 6.51 (s, 1H, Ar), 2.43 (s, 3H, CH_3); $^{13}C\{^1H\}$ NMR (100 MHz, $CDCl_3$, 25 °C): δ = 148.3, 137.0, 135.3, 133.4, 132.9, 129.6, 124.1, 120.9, 119.0, 118.0, 111.4, 109.2, 100.7, 9.2; HRMS (ESI/Q-TOF) m/z $[M + H]^+$ calcd for $C_{17}H_{13}N_2O_2S$: 309.0692; found: 309.0675.

3-([1,1'-biphenyl]-4-yl)-9-methylthiazolo[3,2-*a*]indole 3m.

3m was isolated by ethyl acetate extraction from crude in 89% yield (211 mg). Yellowish solid; mp: 138–140 °C; ^{1}H NMR (400 MHz, $CDCl_3$, 25 °C): δ = 7.78 (d, 2H, *J* = 8.1 Hz, Ar), 7.69-7.75 (m, 4H, Ar), 7.60 (d, 1H, *J* = 8.0 Hz, Ar), 7.52 (t, 2H, *J* = 7.6 Hz, Ar), 7.43 (t, 1H, *J* = 7.2 Hz, Ar), 7.21 (t, 1H, *J* = 7.6 Hz, Ar), 7.16 (d, 1H, *J* = 8.4 Hz, Ar), 6.95 (t, 1H, *J* = 8.0 Hz, Ar), 6.45 (s, 1H, Ar), 2.42 (s, 3H, CH_3); $^{13}C\{^1H\}$ NMR (100 MHz, $CDCl_3$, 25 °C): δ = 142.4, 140.2, 135.5, 133.0, 130.1, 129.7, 129.6, 129.5, 129.0, 127.8, 127.4, 127.2, 127.1, 120.7, 118.7, 117.7, 111.8, 106.7, 9.5; HRMS (ESI/Q-TOF) m/z $[M + H]^+$ calcd for $C_{23}H_{18}NS$: 340.1154; found: 340.1162.

2,9-Dimethyl-3-phenylthiazolo[3,2-*a*]indole 3n.

3n was isolated by ethyl acetate extraction from crude in 97% yield (189 mg). Yellowish solid; mp: 98–100°C; ^{1}H NMR (400 MHz, $CDCl_3$, 25 °C): δ = 7.51–7.59 (m, 6H, Ar), 7.14 (dt, 1H, *J* = 6.8 Hz, J = 0.8 Hz, Ar), 6.84 (dt, 1H, *J* = 7.2 Hz, *J* = 1.2 Hz, Ar), 6.67 (d, 1H, *J* = 7.6 Hz, Ar), 2.42 (s, 3H, CH_3), 2.25 (s, 3H, CH_3); $^{13}C\{^1H\}$ NMR (100 MHz, $CDCl_3$, 25 °C): δ = 133.9, 132.0, 130.5, 130.2, 130.1, 130.0, 129.4, 128.9, 120.0, 118.4, 117.8, 117.4, 111.1, 98.5, 13.3, 9.4; HRMS (ESI/Q-TOF) m/z $[M + H]^+$ calcd for $C_{18}H_{16}NS$: 278.0998; found: 278.1012.

9-Methyl-2,3-diphenylthiazolo[3,2-*a*]indole 3o.

3o was isolated by column chromatography on silica gel (acetate/cyclohexane) in 94% yield (221 mg). Yellowish solid; mp: 150–152 °C; ^{1}H NMR (400 MHz, $CDCl_3$, 25 °C): δ = 7.55–7.62 (m, 6H, Ar), 7.15-7.27 (m, 6H, Ar), 6.85 (t, 1H, *J* = 7.2 Hz, Ar), 6.55 (d, 1H, *J* = 8.4 Hz, Ar), 2.46 (s, 3H, CH_3); $^{13}C\{^1H\}$ NMR (100 MHz, $CDCl_3$, 25 °C): δ = 132.9, 132.6, 132.4, 130.8, 130.4, 130.0, 129.8, 129.3, 128.4, 128.2, 127.3, 121.5, 120.6, 118.6, 117.4, 111.4, 99.6, 9.3; HRMS (ESI/Q-TOF) m/z $[M + H]^+$ calcd for $C_{23}H_{18}NS$: 340.1154; found: 340.1167.

11-Methyl-6,7,8,9-tetrahydrobenzo[4,5]thiazolo[3,2-*a*]indole 3p.

3p was isolated by column chromatography on silica gel (acetate/cyclohexane) in 77% yield (131 mg). Yellowish solid; mp: 75–77 °C; ^{1}H NMR (400 MHz, $CDCl_3$) δ = 7.71 (d, 1H, *J* = 8.2 Hz, Ar), 7.57 (dd, 1H, *J* = 8.0 Hz, *J* = 0.7 Hz, Ar), 7.17–7.23 (m, 1H, Ar),7.09 (ddd, 1H, *J* = 8.2 Hz, *J* = 7.1 Hz, *J* = 1.2 Hz, Ar), 3.06–3.10 (m, 2H, Cycloalk.), 2.66–2.70 (m, 2H, Cycloalk.), 2.38 (s, 3H, CH_3), 1.97–2.05 (m, 2H, Cycloalk.), 1.97–1.89 (m, 2H, Cycloalk.); ^{13}C{^{1}H} NMR (100 MHz, $CDCl_3$) δ = 133.9, 131.9, 130.0, 128.8, 119.8, 118.5, 117.5, 117.0, 110.7, 98.8, 24.8, 24.2, 22.9, 22.1, 9.4; HRMS (ESI/Q-TOF) m/z $[M + H]^+$ calcd for $C_{15}H_{16}NS$: 242.0998; found: 242.0981.

Ethyl 9-methylthiazolo[3,2-*a*]indole-3-carboxylate 3q.

3q was isolated by column chromatography on silica gel (acetate/cyclohexane) in 85% yield (154 mg). Yellowish oil; ^{1}H NMR (400 MHz, $CDCl_3$, 25 °C): δ = 8.64 (dd, 1H, *J* = 8.8 Hz, *J* = 0.8 Hz, Ar), 7.56–7.60 (m, 1H, Ar), 7.55 (s, 1H, Ar), 7.27–7.31(m, 1H, Ar), 720–7.24 (m, 1H, Ar), 4.47 (q, 2H, *J* = 7.2 Hz, OCH_2CH$_3$), 2.40 (s, 3H, CH_3), 1.46 (t, 3H, *J* = 7.2 Hz, OCH$_2$$CH_3$); ^{13}C{^{1}H} NMR (100 MHz, $CDCl_3$, 25 °C): δ = 158.6, 134.3, 132.5, 130.9, 128.2, 121.4, 121.0, 119.6, 117.2, 114.8, 101.1, 61.7, 14.3, 9.2; HRMS (ESI/Q-TOF) m/z $[M + H]^+$ calcd for $C_{14}H_{14}NO_2S$: 260.0740; found: 260.0755.

Ethyl 8-bromo-3,9-dimethylthiazolo[3,2-*a*]indole-2-carboxylate 3r.

3r was isolated by column chromatography on silica gel (acetate/cyclohexane) in 68% yield (165 mg). Yellowish solid; mp: 70–72 °C; ^{1}H NMR (400 MHz, $DMSO_{d6}$, 25 °C): δ = 8.03 (d, 1H, *J* = 8.4 Hz, Ar), 7.47 (d, 1H, *J* = 7.6 Hz, Ar), 7.07 (d, 1H, *J* = 8.0 Hz, Ar), 4.31 (q, 2H, *J* = 7.2 Hz, OCH_2CH$_3$), 3.09 (s, 3H, CH_3), 2.51 (s, 3H, CH_3), 1.32 (t, 3H, *J* = 7.2 Hz, OCH$_2$$CH_3$); ^{13}C{^{1}H} NMR (100 MHz, $CDCl_3$, 25 °C): δ = 162.6, 141.0, 135.6, 131.9, 131.2, 130.3, 126.6, 120.2, 113.1, 111.1, 101.5, 61.3, 14.3, 14.2, 12.4; HRMS (ESI/Q-TOF) m/z $[M + H]^+$ calcd for $C_{15}H_{15}BrNO_2S$: 352.0001; found: 352.0017.

Ethyl 7-methoxy-3,9-dimethylthiazolo[3,2-*a*]indole-2-carboxylate 3s.

3s was isolated by ethyl acetate extraction from crude in in 99% yield (211 mg). White solid; mp: 96–98 °C; ^{1}H NMR (400 MHz, $CDCl_3$, 25 °C): δ = 7.74 (d, 1H, *J* = 9.2 Hz, Ar), 6.98 (d, 1H, *J* = 2.4 Hz, Ar), 6.80 (dd, 1H, *J* = 8.8 Hz, *J* = 2.4 Hz, Ar), 4.36 (q, 2H, *J* = 7.2 Hz, OCH_2CH$_3$), 3.91 (s, 3H, OCH_3), 3.08 (s, 3H, CH_3), 2.30 (s, 3H, CH_3), 1.40 (t, 3H, *J* = 7.2 Hz, OCH$_2$$CH_3$); ^{13}C{^{1}H} NMR (100 MHz, $CDCl_3$, 25 °C): δ = 163.0, 155.7, 141.2, 135.0, 134.1, 125.89, 112.6, 109.2, 109.0, 100.3, 99.8, 61.0, 55.7, 14.4, 13.8, 9.2; HRMS (ESI/Q-TOF) m/z $[M + H]^+$ calcd for $C_{16}H_{18}NO_3S$: 304.1002; found: 304.0985.

Ethyl 7-Fluoro-3,9-dimethylthiazolo[3,2-*a*]indole-2-carboxylate 3t.

3t was isolated by ethyl acetate extraction from crude in in 90% yield (181 mg). Yellowish solid; mp: 125–127 °C; ^{1}H NMR (400 MHz, $CDCl_3$, 25 °C): δ = 7.77 (dd, 1H, *J* = 9.2 Hz, *J* = 4.0 Hz, Ar), 7.19 (dd, 1H, *J* = 9.2 Hz, *J* = 2.4 Hz, Ar), 6.90 (dt, 1H, *J* = 8.8 Hz, *J* = 2.4 Hz, Ar), 4.36 (q, 2H, *J* = 7.2 Hz, OCH_2CH3), 3.08 (s, 3H, CH_3), 2.29 (s, 3H, CH_3), 1.40 (t, 3H, *J* = 7.2 Hz, OCH$_2$$CH_3$); ^{13}C{^{1}H} NMR (100 MHz, $CDCl_3$, 25 °C): δ = 162.8, 159.1 ($J_{C\text{-}F}$ = 237.6 Hz), 141.1, 135.2, 134.9 ($J_{C\text{-}F}$ = 9.9 Hz), 127.3, 112.6 ($J_{C\text{-}F}$ = 9.8 Hz), 110.0, 107.6 ($J_{C\text{-}F}$ = 26.0 Hz), 103.0 ($J_{C\text{-}F}$ = 23.6 Hz), 100.5 ($J_{C\text{-}F}$ = 4.3 Hz), 61.2, 13.9, 9.2; HRMS (ESI/Q-TOF) m/z $[M + H]^+$ calcd for $C_{15}H_{15}FNO_2S$: 292.0802; found: 292.0821.

Ethyl 7-Chloro-3,9-dimethylthiazolo[3,2-*a*]indole-2-carboxylate 3u.

3u was isolated by column chromatography on silica gel (acetate/cyclohexane) in 67% yield (146 mg). Yellowish solid; mp: 135–137 °C; ^{1}H NMR (400 MHz, $CDCl_3$, 25 °C): δ = 7.76 (dd, 1H, *J* = 8.8 Hz, *J* = 0.4 Hz, Ar), 7.52 (dd, 1H, *J* = 2.0 Hz, J = 0.4 Hz, Ar), 7.12 (dd, 1H, *J* = 8.8 Hz, *J* = 2.0 Hz, Ar), 4.37 (q, 2H, *J* = 7.2 Hz, O$CH_2$$CH_3$), 3.09 (s, 3H, CH_3), 2.30 (s, 3H, CH_3), 1.40 (t, 3H, *J* = 7.2 Hz, $OCH_2$$CH_3$); ^{13}C{^{1}H} NMR (100 MHz, $CDCl_3$, 25 °C): δ = 162.7, 141.0, 135.0, 134.9, 129.0, 128.0, 119.7, 117.3, 112.7, 110.7, 100.2, 61.3, 14.3, 13.9, 9.1; HRMS (ESI/Q-TOF) m/z $[M + H]^+$ calcd for $C_{15}H_{15}ClNO_2S$: 308.0507; found: 308.0530.

Ethyl 6-chloro-3,9-dimethylthiazolo[3,2-*a*]indole-2-carboxylate 3v.

3v was isolated by column chromatography on silica gel (acetate/cyclohexane) in 73% yield (153 mg). Yellowish solid; mp: 83–85 °C; ^{1}H NMR (400 MHz, $DMSO_{d6}$, 25 °C): δ = 8.04 (d, 1H, *J* = 1.6 Hz, Ar), 7.63 (d, 1H, *J* = 8.4 Hz, Ar), 7.33 (dd, 1H, *J* = 8.4 Hz, *J* = 1.6 Hz, Ar), 4.32 (q, 2H, *J* = 7.2 Hz, O$CH_2$$CH_3$), 3.12 (s, 3H, CH_3), 2.30 (s, 3H, CH_3), 1.32 (t, 3H, *J* = 7.2 Hz, $OCH_2$$CH_3$); ^{13}C{^{1}H} NMR (100 MHz, $CDCl_3$, 25 °C): δ = 162.6, 140.9, 133.9, 132.7, 131.1, 125.2, 118.8, 114.8, 112,9, 100.9, 61.3, 14.3, 13.9, 9.1; HRMS (ESI/Q-TOF) m/z $[M + H]^+$ calcd for $C_{15}H_{15}ClNO_2S$: 308.0507; found: 308.0514.

Ethyl 6-bromo-3,9-dimethylthiazolo[3,2-*a*]indole-2-carboxylate 3w.

3w was isolated by ethyl acetate extraction from crude in in 96% yield (237 mg). Yellow oil; ^{1}H NMR (400 MHz, $DMSO_{d6}$, 25 °C): δ = 8.29 (d, 1H, *J* = 1.2 Hz, Ar), 7.72 (d, 1H, *J* = 8.8 Hz, Ar), 7.59 (dd, 1H, *J* = 8.4 Hz, *J* = 1.6 Hz, Ar), 4.46 (q, 2H, *J* = 7.2 Hz, O$CH_2$$CH_3$), 3.25 (s, 3H, CH_3), 2.43 (s, 3H, CH_3), 1.46 (t, 3H, *J* = 7.2 Hz, $OCH_2$$CH_3$); ^{13}C{^{1}H} NMR (100 MHz, $CDCl_3$, 25 °C): δ = 162.6, 140.9, 133.9, 132.7, 131.1, 125.2, 118.8, 114.8, 112.9, 110.9, 100.6, 61.3, 14.3, 13.9, 9.1; HRMS (ESI/Q-TOF) m/z $[M + H]^+$ calcd for $C_{15}H_{15}BrNO_2S$: 352.0001; found: 352.0010.

Ethyl 3-methyl-9-propylthiazolo[3,2-*a*]indole-2-carboxylate 3x.

3x was isolated by ethyl acetate extraction from crude in in 92% yield (190 mg). Yellowish oil; ^{1}H NMR (400 MHz, $CDCl_3$, 25 °C): δ = 7.89 (d, 1H, *J* = 8.0 Hz, Ar), 7.60 (d, 1H, *J* = 8.0 Hz, Ar), 7.30 (dt, 1H, *J* = 8.0 Hz, *J* = 0.8 Hz, Ar), 7.18 (dt, 1H, *J* = 7.2 Hz, *J* = 1.2 Hz, Ar), 4.36 (q, 2H, *J* = 7.2 Hz, O$CH_2$$CH_3$), 3.14 (s, 3H, CH_3), 2.78 (dt, 2H, *J* = 7.6 Hz, $CH_2$$CH_2CH_3$), 1.80 (sex, 2H, *J* = 7.2 Hz, $CH_2$$CH_2$$CH_3$), 1.41 (t, 3H, *J* = 7.2 Hz, $OCH_2$$CH_3$), 1.04 (t, 3H, *J* = 7.6 Hz, $CH_2CH_2$$CH_3$); ^{13}C{^{1}H} NMR (100 MHz, $CDCl_3$, 25 °C): δ = 162.9, 141.4, 133.7, 133.0, 130.8, 122.0, 119.6, 118.0, 112.0, 109.9, 105.5, 61.1, 26.7, 22.1, 14.4, 14.2, 14.0; HRMS (ESI/Q-TOF) m/z $[M + H]^+$ calcd for $C_{17}H_{20}NO_2S$: 302.1209; found: 302.1201.

Ethyl 9-decyl-3-methylthiazolo[3,2-a]indole-2-carboxylate 3y

3y was isolated by ethyl acetate extraction from crude in in 99% yield (273 mg). Yellowish solid; mp: 55–57 °C; ^{1}H NMR (400 MHz, $CDCl_3$, 25 °C): δ = 7.89 (d, 1H, *J* = 8.4 Hz, *J* = 0.8 Hz, Ar), 7.60 (dd, 1H, *J* = 8.0 Hz, *J* = 0.8 Hz, Ar), 7.30 (dt, 1H, *J* = 8.0 Hz, *J* = 0.8 Hz, Ar), 7.18 (dt, 1H, *J* = 7.2 Hz, *J* = 0.8 Hz, Ar), 4.37 (q, 2H, *J* = 7.2 Hz, O$CH_2$$CH_3$), 3.14 (s, 3H, CH_3), 2.79 (t, 2H, *J* = 7.6 Hz, decyl), 1.76 (quint., 2H, *J* = 6.8 Hz, decyl), 1.41 (t, 3H, *J* = 7.2 Hz, $OCH_2$$CH_3$), 1.23–1.34 (m, 12H, decyl), 0.86–0.92 (m, 5H, decyl); ^{13}C{^{1}H} NMR (100 MHz, $CDCl_3$, 25 °C): δ = 162.9, 141.4, 133.6, 132.9, 130.8, 122.0, 119.6, 118.0, 112.0,

109.9, 105.8, 61.1, 31.9, 29.6, 29.5, 29.3, 28.7, 24.6, 22.7, 14.4, 14.1, 14.0; HRMS (ESI/Q-TOF) m/z $[M + H]^+$ calcd for $C_{24}H_{34}NO_2S$: 400.2305; found: 400.2298.

Ethyl 3-methyl-9-(3-phenylpropyl)thiazolo[3,2-a]indole-2-carboxylate 3z

3z was isolated by column chromatography on silica gel (acetate/cyclohexane) in 74% yield (192 mg). Yellow oil; ^1H NMR (400 MHz, $CDCl_3$, 25 °C): δ = 7.90 (dd, 1H, *J* = 8.0 Hz, *J* = 0.8 Hz, Ar), 7.57 (dt, 1H, *J* = 7.6 Hz, *J* = 0.8 Hz, Ar), 7.28–7.32 (m, 3H, Ar), 7.17–7.21 (m, 4H, Ar), 4.37 (q, 2H, *J* = 7.2 Hz, OCH_2CH_3), 3.15 (s, 3H, CH_3), 2.86 (t, 2H, *J* = 7.2 Hz, $CH_2CH_2CH_2Ph$), 2.71 (t, 2H, *J* = 7.6 Hz, $CH_2CH_2CH_2Ph$), 2.12 (quint, 2H, *J* = 7.2 Hz, $CH_2CH_2CH_2Ph$), 1.41 (t, 3H, *J* = 7.2 Hz, OCH_2CH_3); $^{13}C\{^1H\}$ NMR (100 MHz, $CDCl_3$, 25 °C): δ = 162.9, 142.1, 141.4, 133.6, 133.1, 130.9, 128.4, 128.3, 125.7, 122.1, 119.7, 117.9, 112.0, 110.0, 105.1, 61.1, 35.7, 30.2, 24.1, 14.4, 14.1, 14.0; HRMS (ESI/Q-TOF) m/z $[M + H]^+$ calcd for $C_{23}H_{24}NO_2S$: 378.1522; found: 378.1504.

4.2 Spectral data of compounds 6a, 8a,b, 10a.

3,9-Dimethylthiazolo[3,2-*a*]indole-2-carboxylic acid 6a.

6a was isolated by column chromatography on siica gel (acetate/cyclohexane) in 99% yield (118 mg). Yellowish solid; mp: 215–217 °C; ^{1}H NMR (400 MHz, $(CD_3)_2CO$, 25 °C): δ = 11.49 (brs, 1H, OH), 8.00 (d, 1H, *J* = 8.4 Hz, Ar), 7.59 (d, 1H, *J* = 7.6 Hz, Ar), 7.29 (dt, 1H, *J* = 7.2 Hz, *J* = 0.8 Hz, Ar), 7.19 (dt, 1H, *J* = 7.2 Hz, *J* = 1.2 Hz, Ar), 3.16 (s, 3H, CH_3), 2.32 (s, 3H, CH_3); $^{13}C\{^1H\}$ NMR (100 MHz, $(CD_3)_2CO$, 25 °C): δ = 162.9, 141.8, 133.8, 130.9,132.8, 122.0, 119.7, 117.5, 112.2, 109.8, 99.9, 13.1, 8.1; HRMS (ESI/Q-TOF) m/z $[M + H]^+$ calcd for $C_{13}H_{12}NO_2S$: 246.0583; found: 246.0567.

5-Ethyl 1-methyl 3-((tert-butoxycarbonyl)amino)-2,6,11b-trimethyl-3,11b-dihydropyrrolo[2,3-*b*]thiazolo[3,2-*a*]indole-1,5-dicarboxylate 8a.

8a was isolated by column chromatography on silica gel (acetate/cyclohexane) in 70% yield (101 mg). Yellowish solid; mp: 93–95 °C; Notably, compound **8a** at NMR analysis shows two sets of peaks. This fact is probably ascribable to the presence of a second axis along the N–N bond that determines the existence of syn/anti rotamers of carbamates. ^{1}H NMR (400 MHz, DMSO$_{d6}$, 25 °C): δ = 8.96, 9.10, 9.40, 9.46 (4brs, 1H, NH), 7.60–7.65 (m, 1H, Ar), 7.28–7.37 (m, 1H, Ar), 7.19–7.24 (m, 1H, Ar), 7.03–7.08 (m, 1H, Ar), 4.05–4.18 (m, 2H, OCH_2CH$_3$), 3.71 (s, 3H, OCH_3), 2.59, 2.65 (2brs, 3H, CH_3), 1.98, 2.02 (2brs, 3H, CH_3), 1.60, 1.62 (2brs, 3H, CH_3), 1.37, 1.39, 1.42 (3brs, 9H, $C(CH_3)_3$), 1.17–1.24 (m, 3H, OCH$_2$*CH$_3$*); $^{13}C\{^1H\}$ NMR (100 MHz, DMSO$_{d6}$, 25 °C): δ = 165.9, 163.0, 158.6, 157.8, 155.7, 154.7, 143.9, 143.6, 141.2, 141.0, 140.1, 128.1, 128.0, 125.0, 124.9, 124.1, 123.6, 114.0, 113.9, 113.4, 113.2, 111.9, 101.8, 101.3, 101.3, 99.7, 98.2, 80.5, 80.0, 79.7, 59.8, 59.8, 55.8, 55.1, 50.4, 50.3, 27.9, 27.8, 27.6, 23.8, 23.8, 14.7, 14.3, 14.2, 12.7, 11.8; HRMS (ESI/Q-TOF) m/z $[M + H]^+$ calcd for $C_{25}H_{32}N_3O_6S$: 502.2006; found: 502.1987.

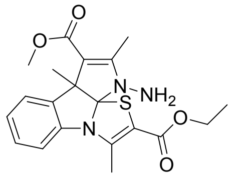

5-Ethyl 1-methyl 3-amino-2,6,11b-trimethyl-3,11b-dihydropyrrolo[2,3-*b*]thiazolo[3,2-*a*]indole-1,5-dicarboxylate 8b.

8b was isolated by column chromatography on silica gel (acetate/cyclohexane) in 67% yield (54 mg). Yellowish oil. °C; ^{1}H NMR (400 MHz, DMSO$_{d6}$, 25 °C): δ = 7.59 (dd, 1H, *J* = 7.6 Hz, *J* = 0.8 Hz, Ar), 7.33 (d, 1H, *J* = 7.6 Hz, Ar), 7.22 (dt, 1H, *J* = 8.0 Hz, *J* = 1.2 Hz, Ar), 7.06 (dt, 1H, *J* = 7.6 Hz, *J* = 0.8 Hz, Ar), 4.50 (s, 2H, NH_2), 4.11–4.16 (m, 2H, OCH_2CH$_3$), 3.67 (s, 3H, OCH_3), 2.67 (s, 3H, CH_3), 2.13 (s, 3H, CH_3), 1.58 (s, 3H, CH_3), 1.22 (t, 3H, *J* = 7.2 Hz, OCH$_2$*CH$_3$*); $^{13}C\{^1H\}$ NMR (100 MHz, DMSO$_{d6}$, 25 °C): δ = 166.2, 163.1, 160.1, 144.9, 141.2, 140.4, 127.9, 125.1, 124.3, 114.1, 112.8, 99.1, 98.1, 59.9, 55.4, 50.0, 24.2, 14.5, 14.3, 12.6; HRMS (ESI/Q-TOF) m/z $[M + H]^+$ calcd for $C_{20}H_{24}N_3O_4S$: 402.1482; found: 402.1495.

4-(*Tert*-butyl) 6-ethyl 7,12b-dimethyl-2-phenyl-1,12b-dihydro-4*H*-pyridazino[3,4-*b*]thiazolo[3,2-*a*]indole-4,6-dicarboxylate 10a.

10a was isolated by column chromatography on silica gel (acetate/cyclohexane) in 62% yield (92 mg). Yellowish solid; mp: 104–106 °C; ^{1}H NMR (400 MHz, DMSO$_{d6}$, 25 °C): δ = 7.77–7.80 (m, 2H, Ar), 7.37–7.48 (m, 3H, Ar), 7.33 (d, 1H, *J* = 8.0 Hz, Ar), 7.26 (d, 1H, *J* = 7.6 Hz, Ar), 7.21 (t, 1H, *J* = 8.0 Hz, Ar), 7.02 (t, 1H, *J* = 7.2 Hz, Ar), 4.13–4.18 (m, 2H, OCH_2CH$_3$), 3.70 (d, 1H, *J* = 18.0 Hz, CH_2), 2.74 (d, 1H,

J = 18.0 Hz, CH_2), 2.54 (s, 3H, CH_3), 1.43 (s, 9H, $C(CH_3)_3$), 1.41 (s, 3H, CH_3), 1.22 (t, 3H, *J* = 7.2 Hz, $OCH_2\mathit{CH_3}$); $^{13}C\{^1H\}$ NMR (100 MHz, $DMSO_{d6}$, 25 °C): δ = 163.0, 151.6, 145.0, 144.4, 140.8, 138.3, 136.3, 129.2, 128.4, 128.4, 125.4, 124.8, 122.4, 116.1, 101.3, 98.3, 81.4, 59.8, 49.9, 28.1, 27.7, 14.4, 13.5; HRMS (ESI/Q-TOF) m/z $[M + H]^+$ calcd for $C_{28}H_{32}N_3O_4S$: 506.2108; found: 506.2122.

5 NMR spectra

5.1 NMR spectra of compounds 3a–z.

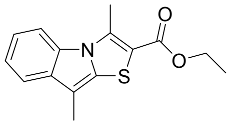

Ethyl 3,9-dimethylthiazolo[3,2-*a*]indole-2-carboxylate 3a.

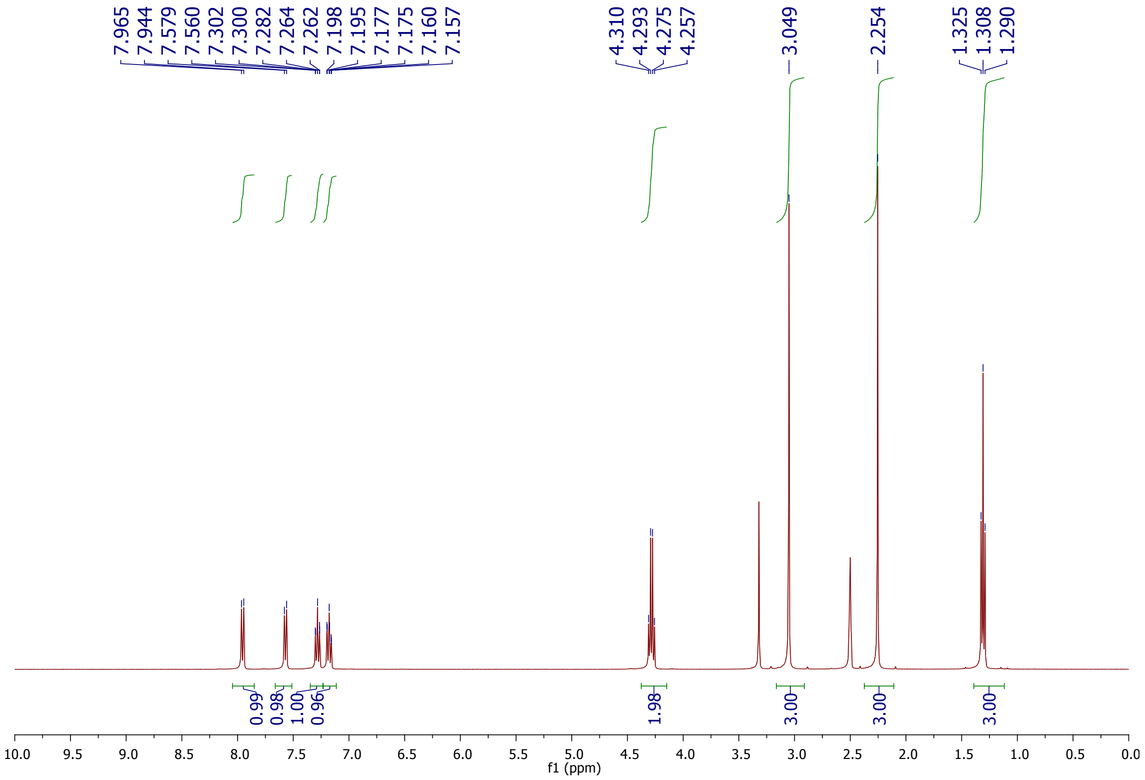

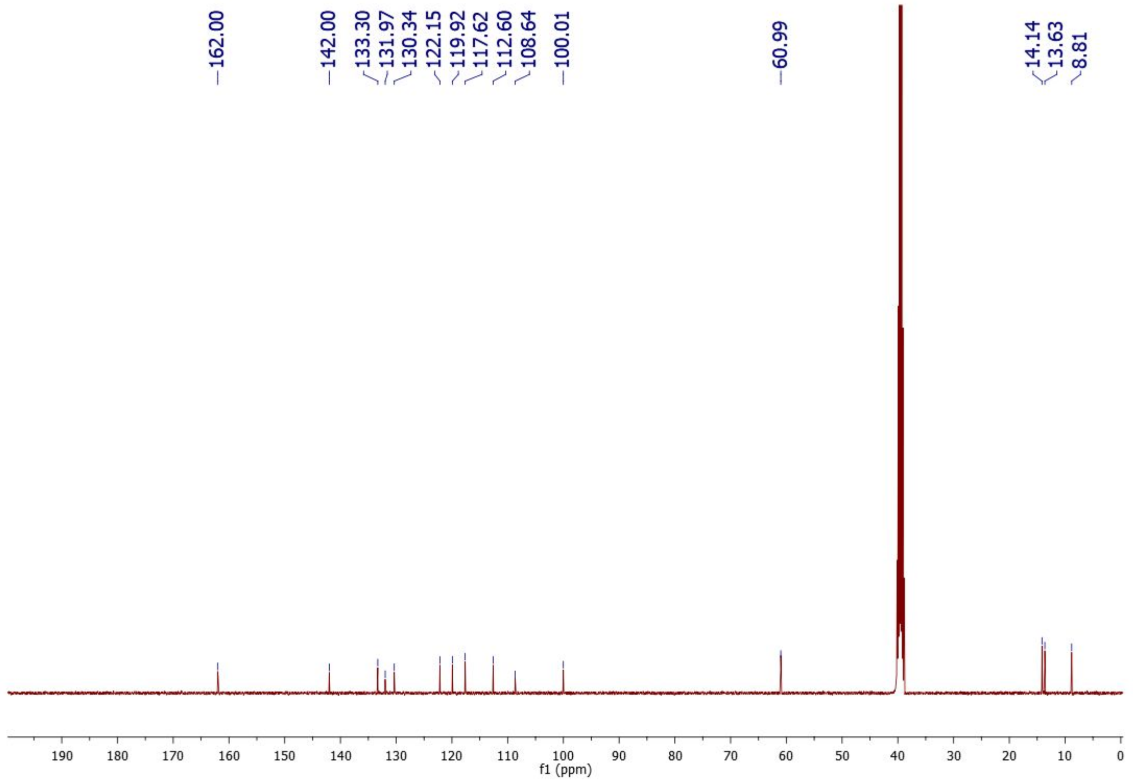

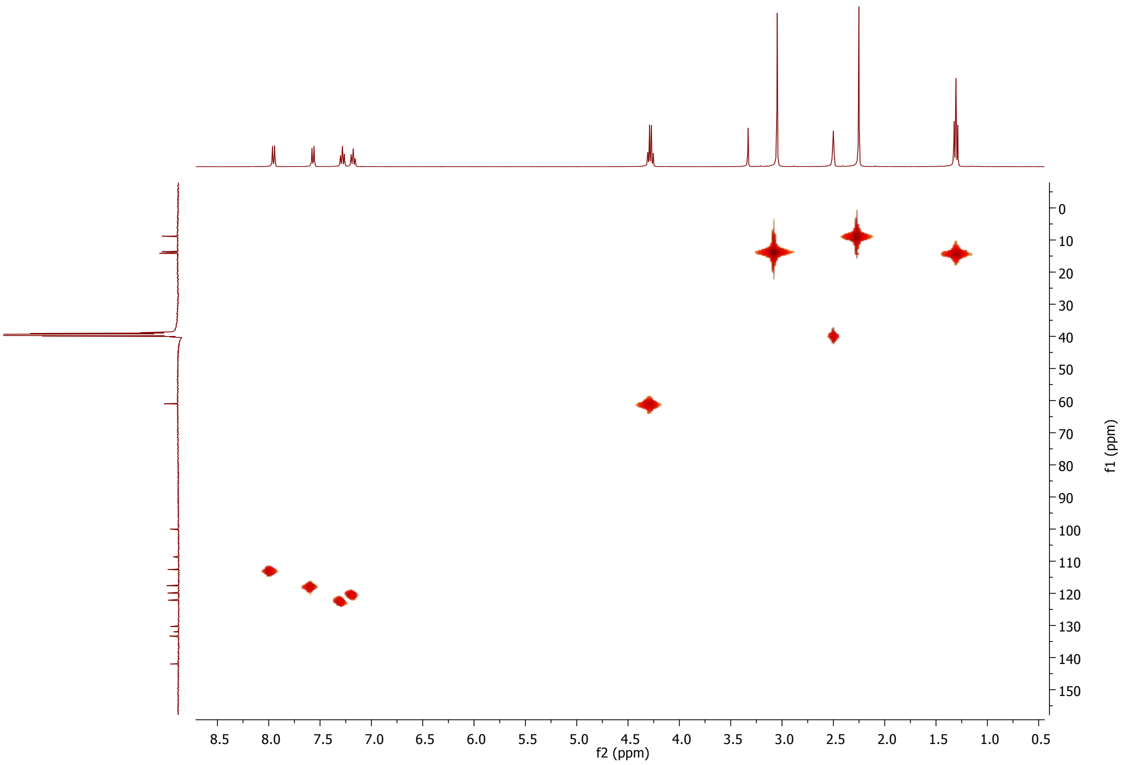

f1 (ppm)
f2 (ppm)

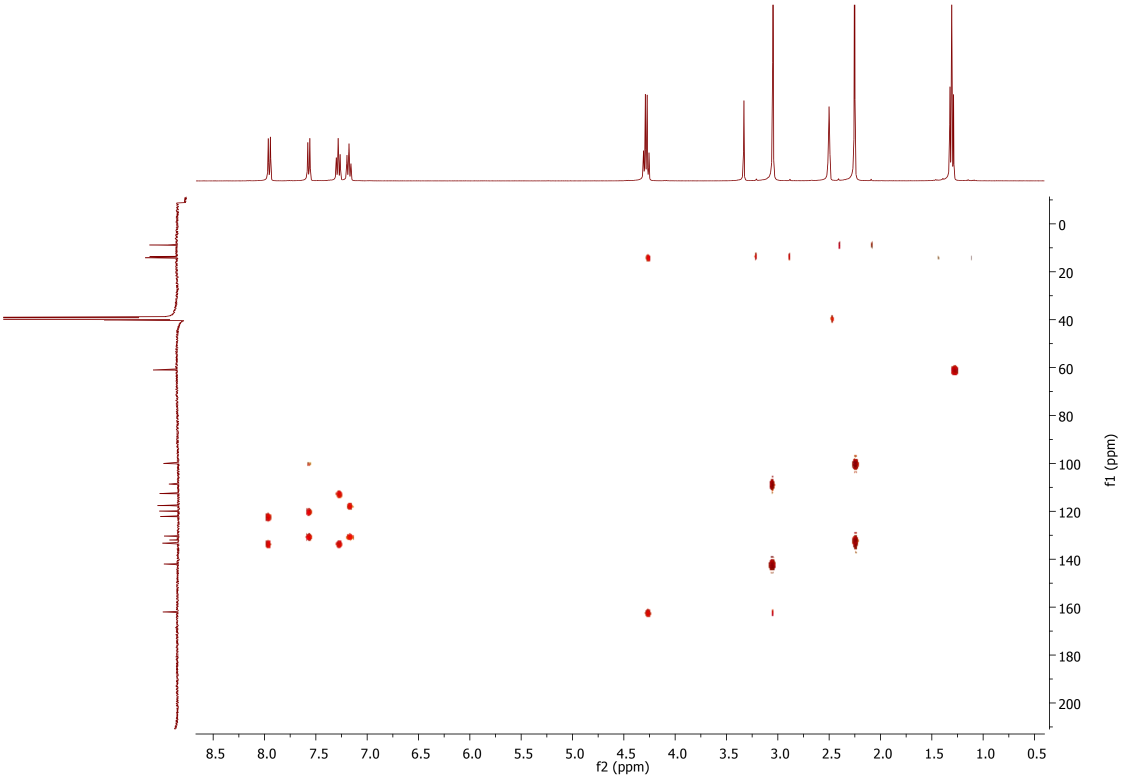

f1 (ppm)
f2 (ppm)

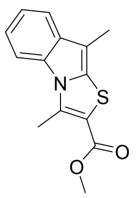

Methyl 3,9-dimethylthiazolo[3,2-*a*]indole-2-carboxylate 3b.

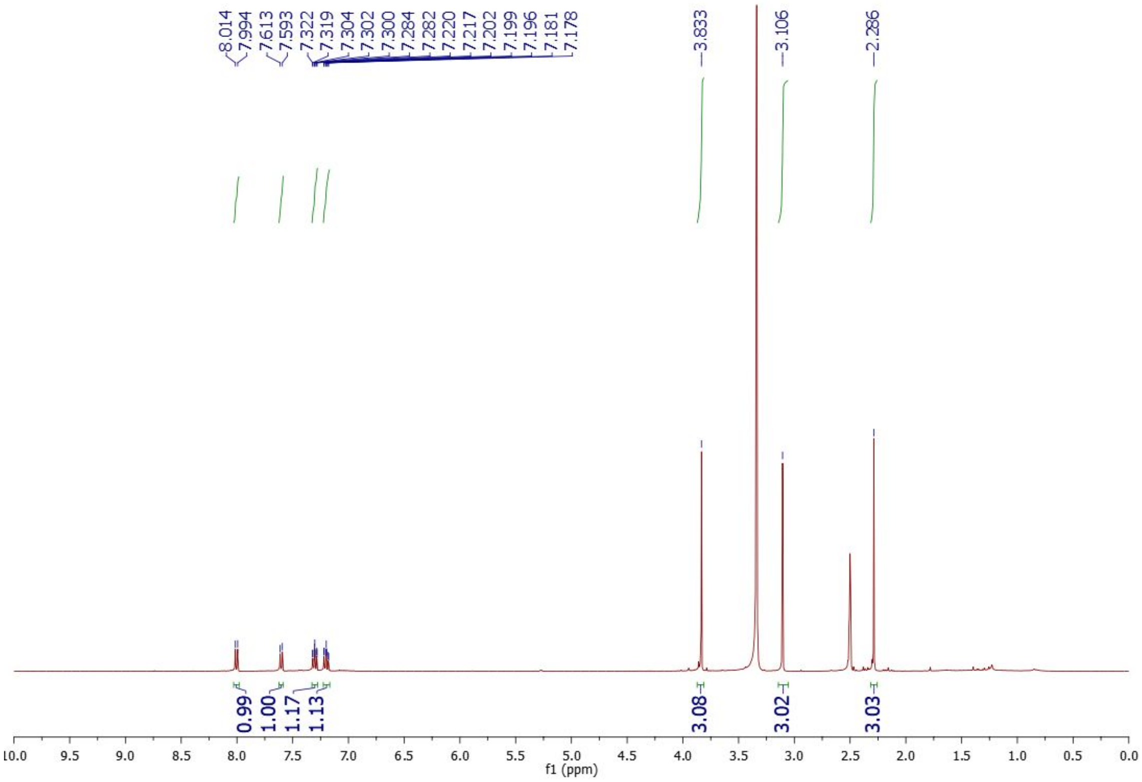

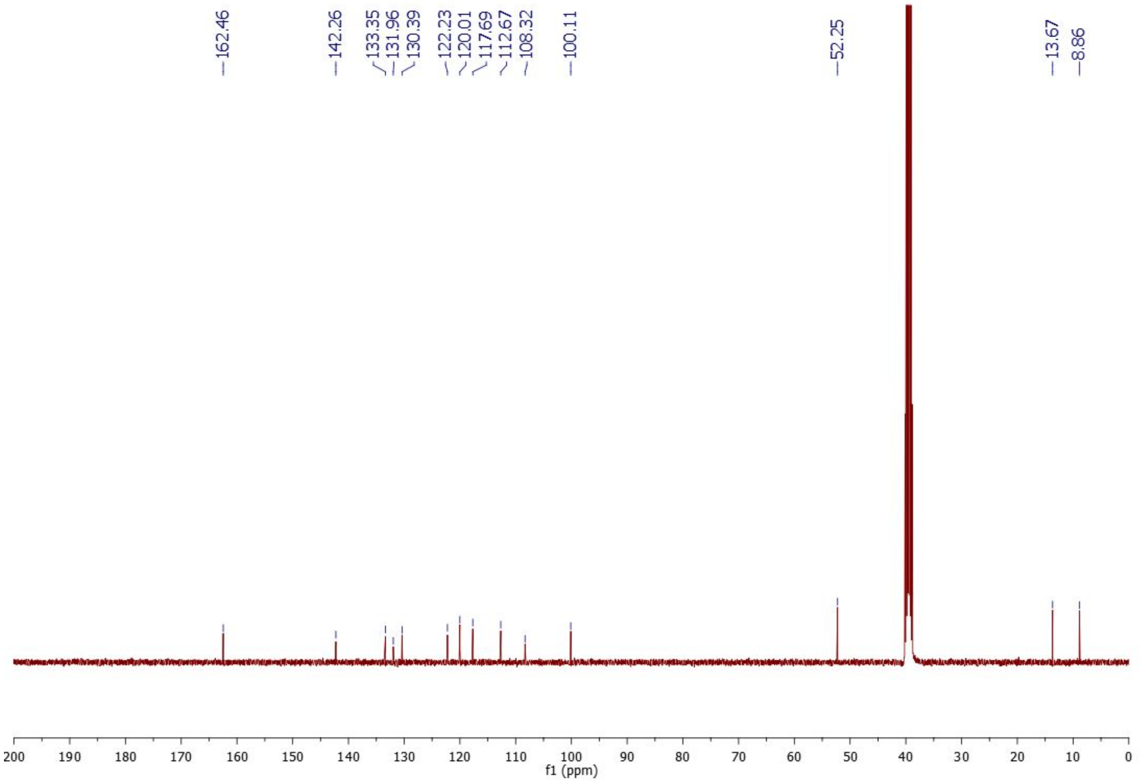

Isopropyl 3,9-dimethylthiazolo [3,2-*a*]indole-2-carboxylate 3c.

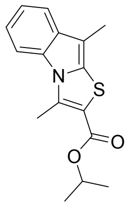

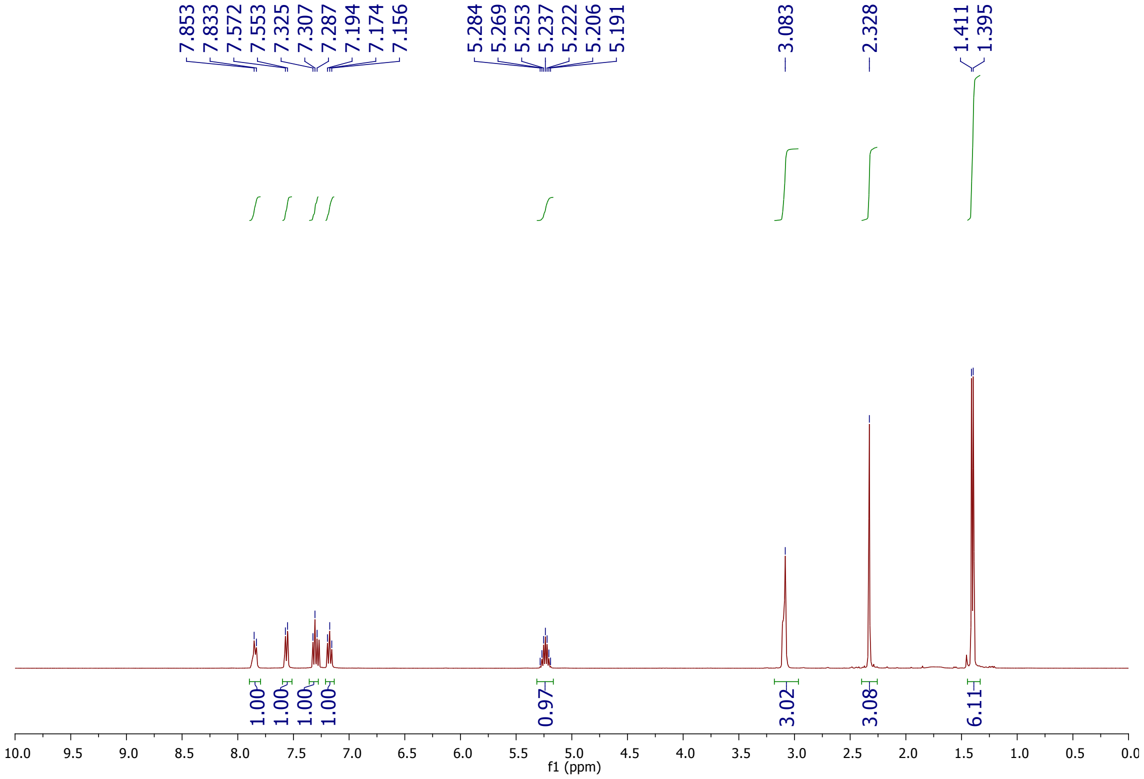

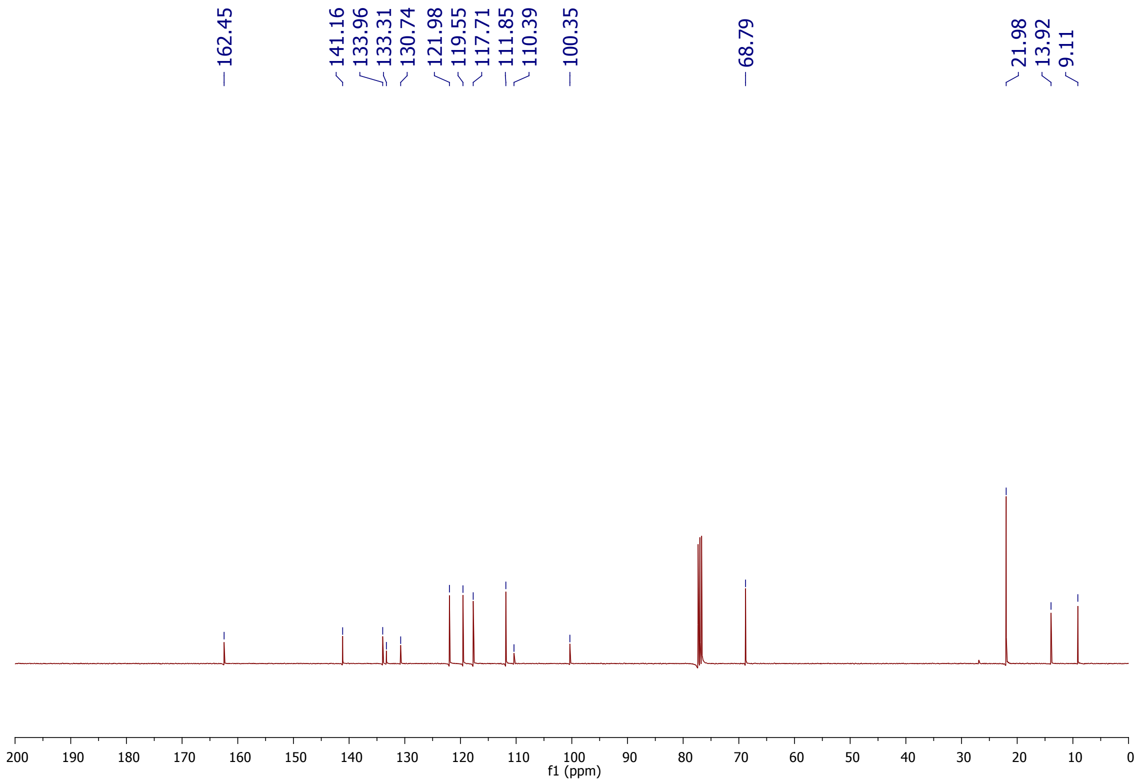

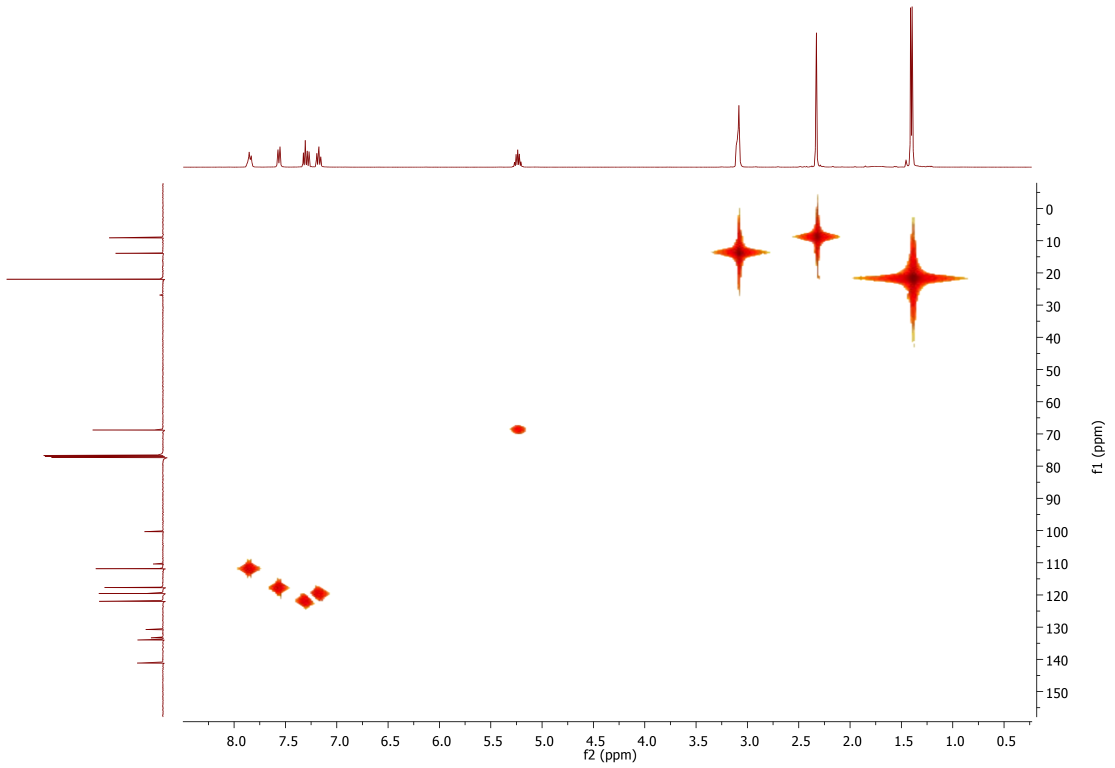
f1 (ppm)
f2 (ppm)

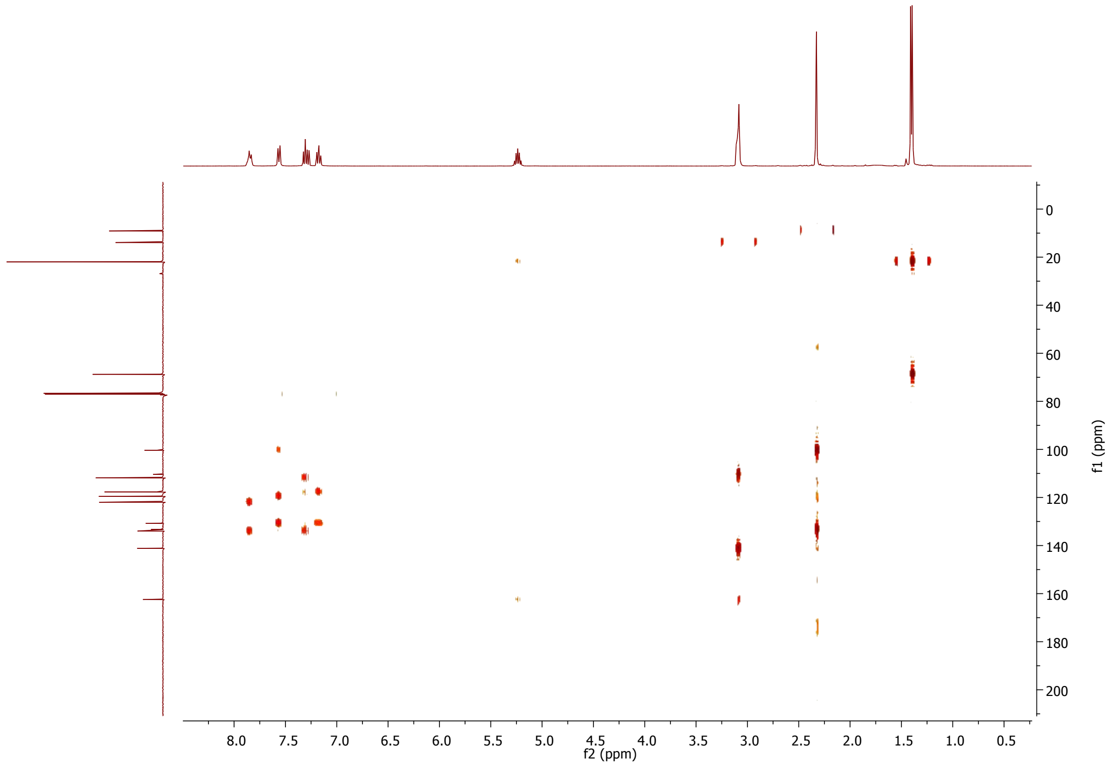
f1 (ppm)
f2 (ppm)

Tert-butyl 3,9-dimethylthiazolo[3,2-_a_]indole-2-carboxylate 3d.

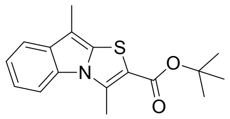

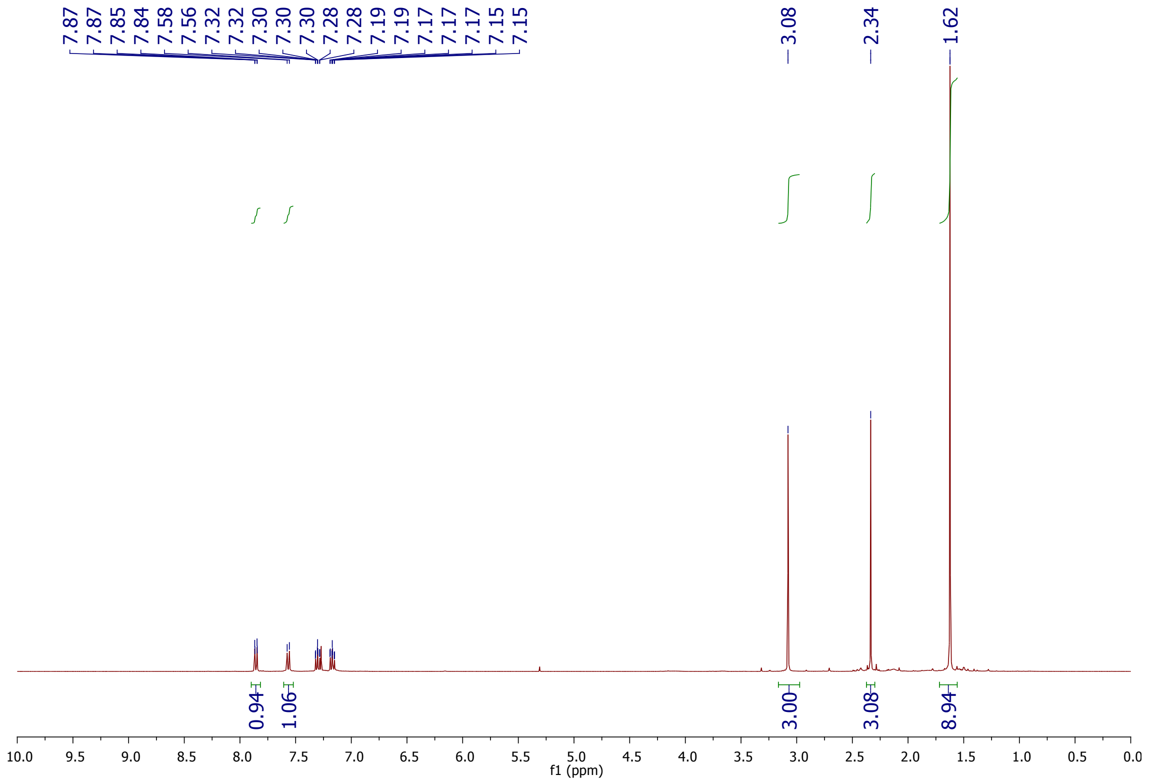

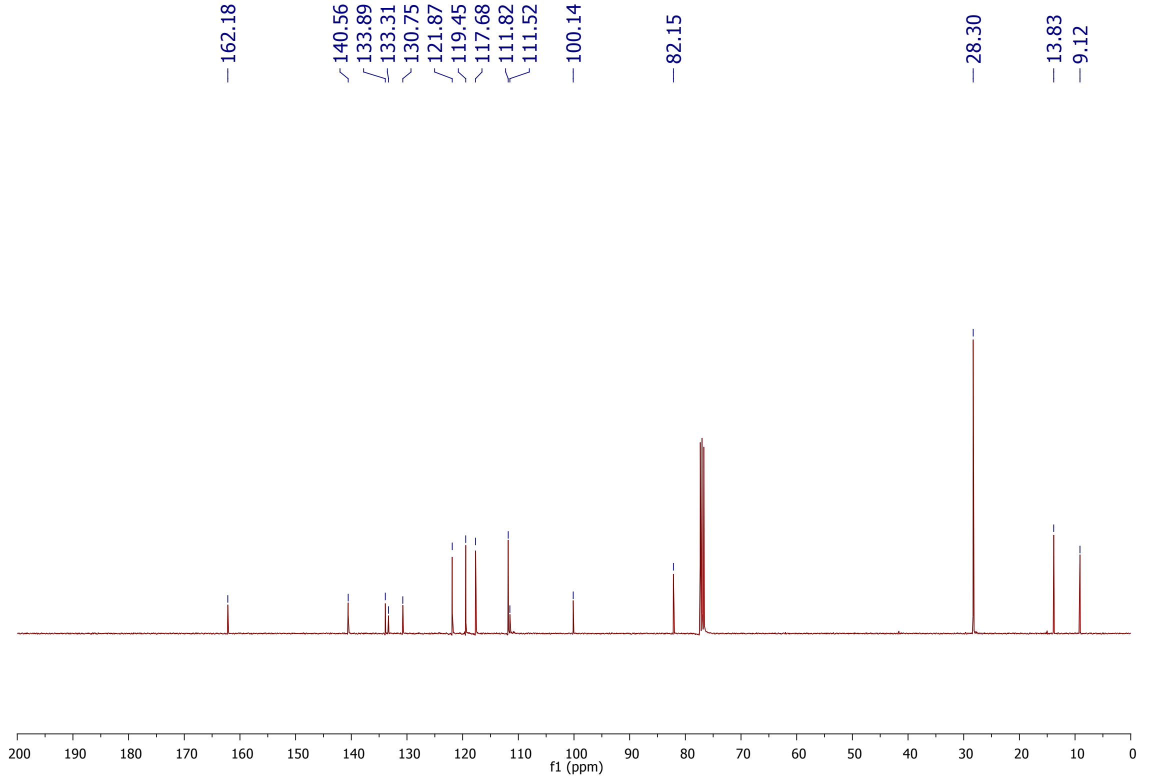

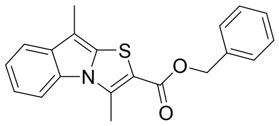

Benzyl 3,9-dimethylthiazolo[3,2-*a*]indole-2-carboxylate 3e.

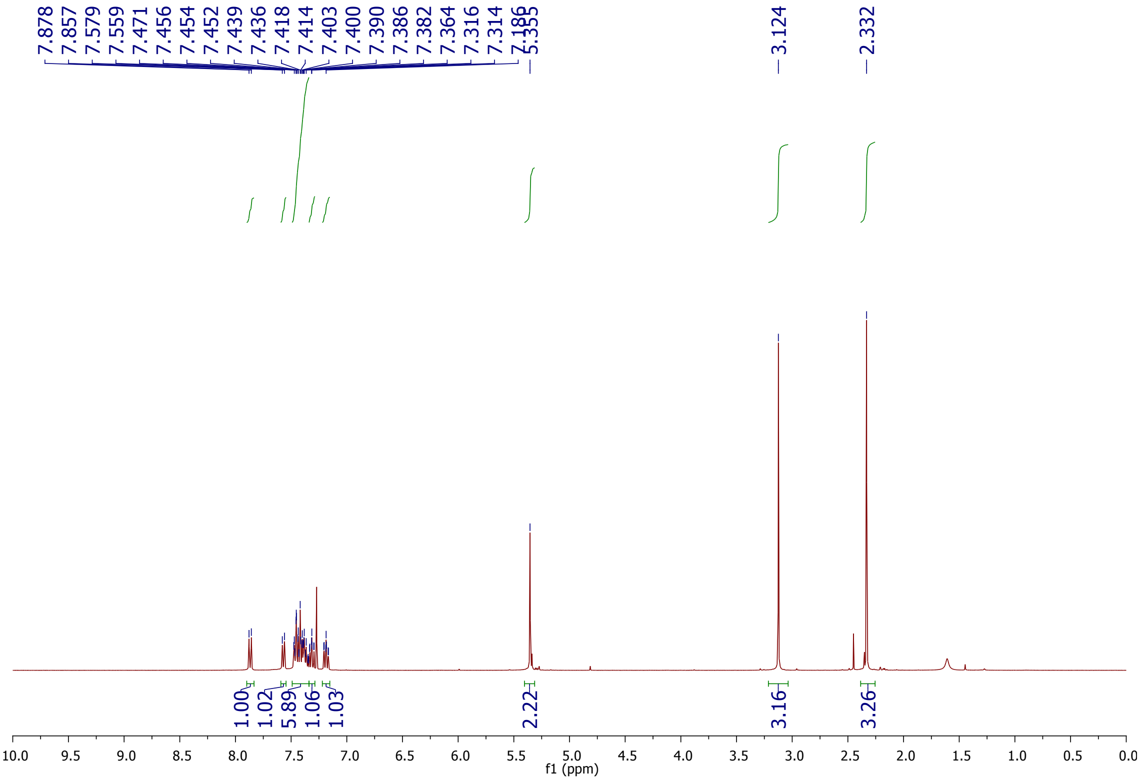

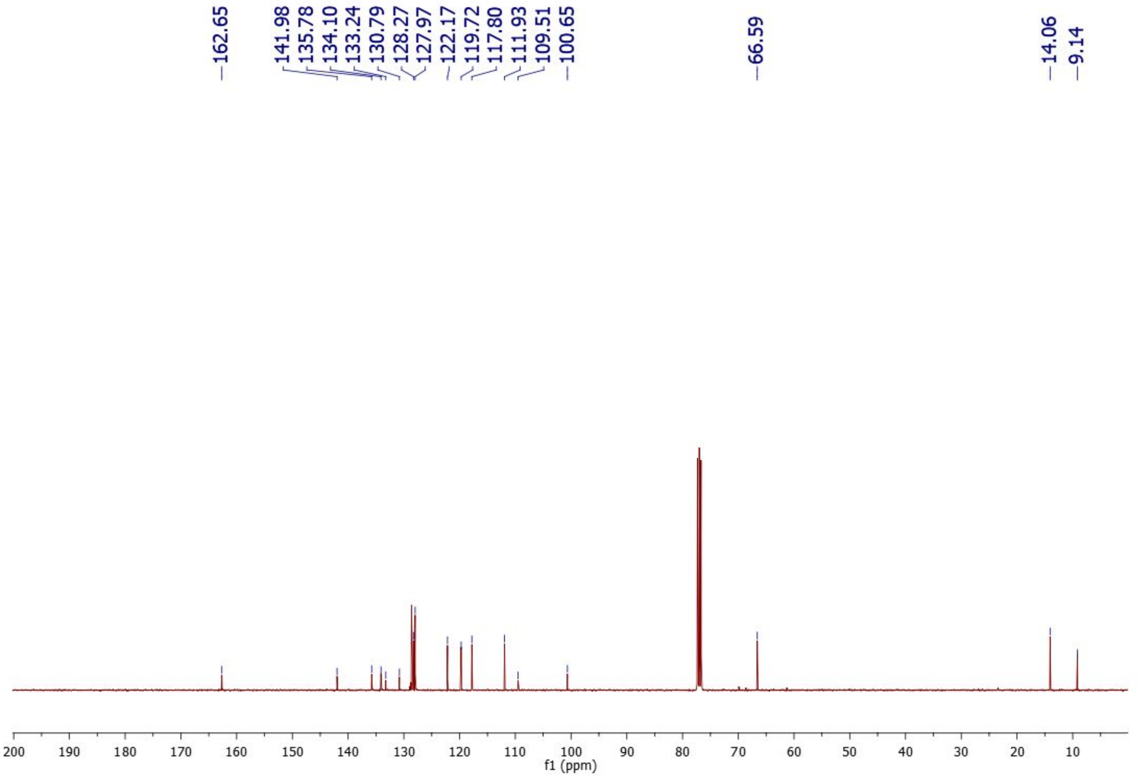

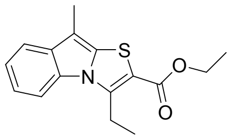

Ethyl 3-ethyl-9-methylthiazolo[3,2-*a*]indole-2-carboxylate 3f.

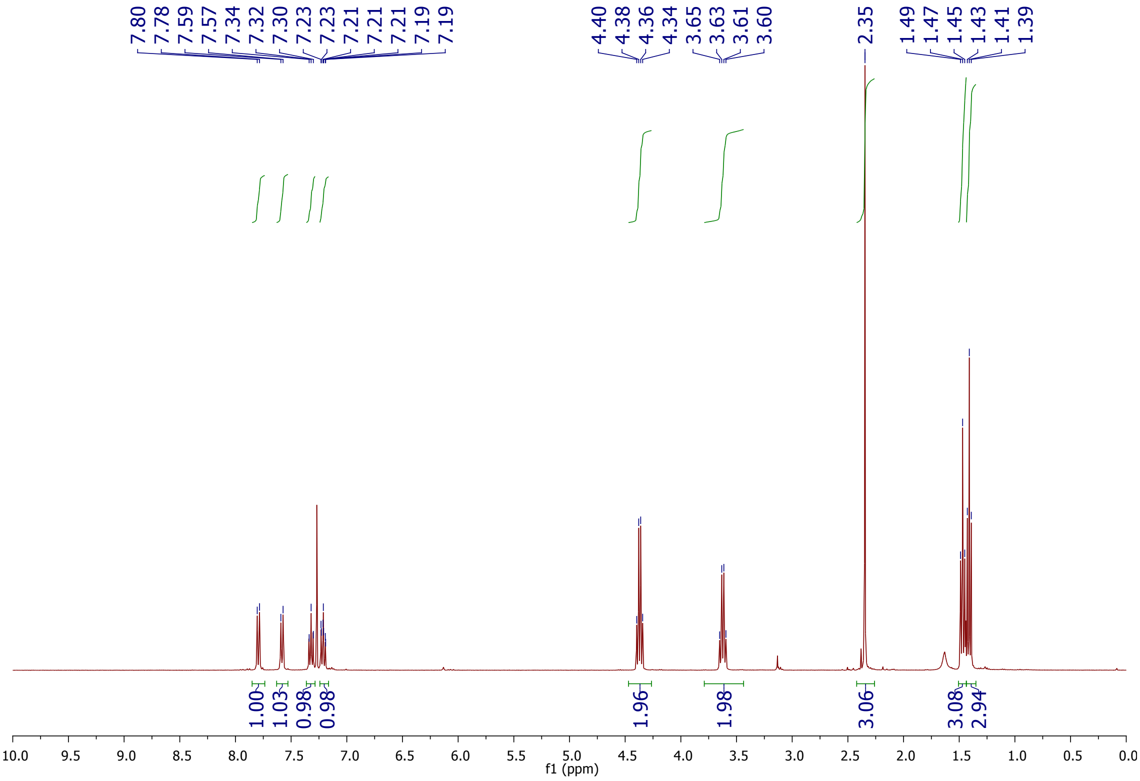

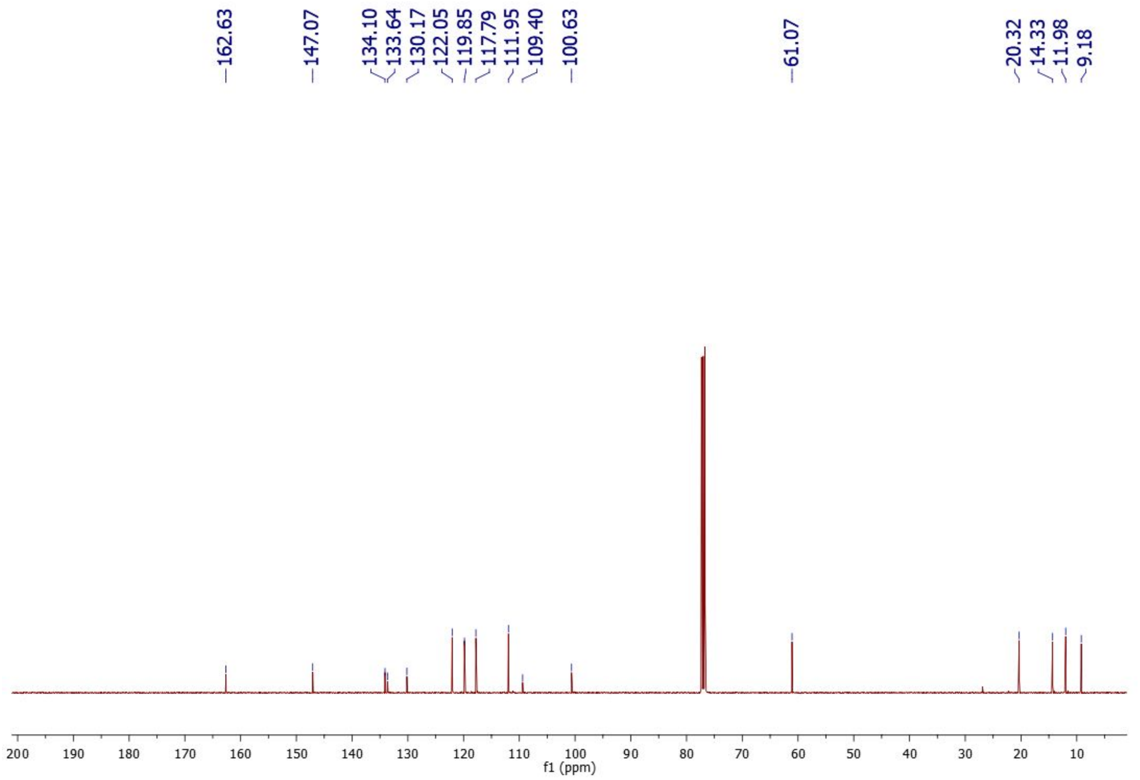

Ethyl 9-methyl-3-phenylthiazolo[3,2-*a*]indole-2-carboxylate 3g.

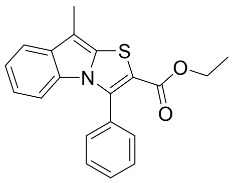

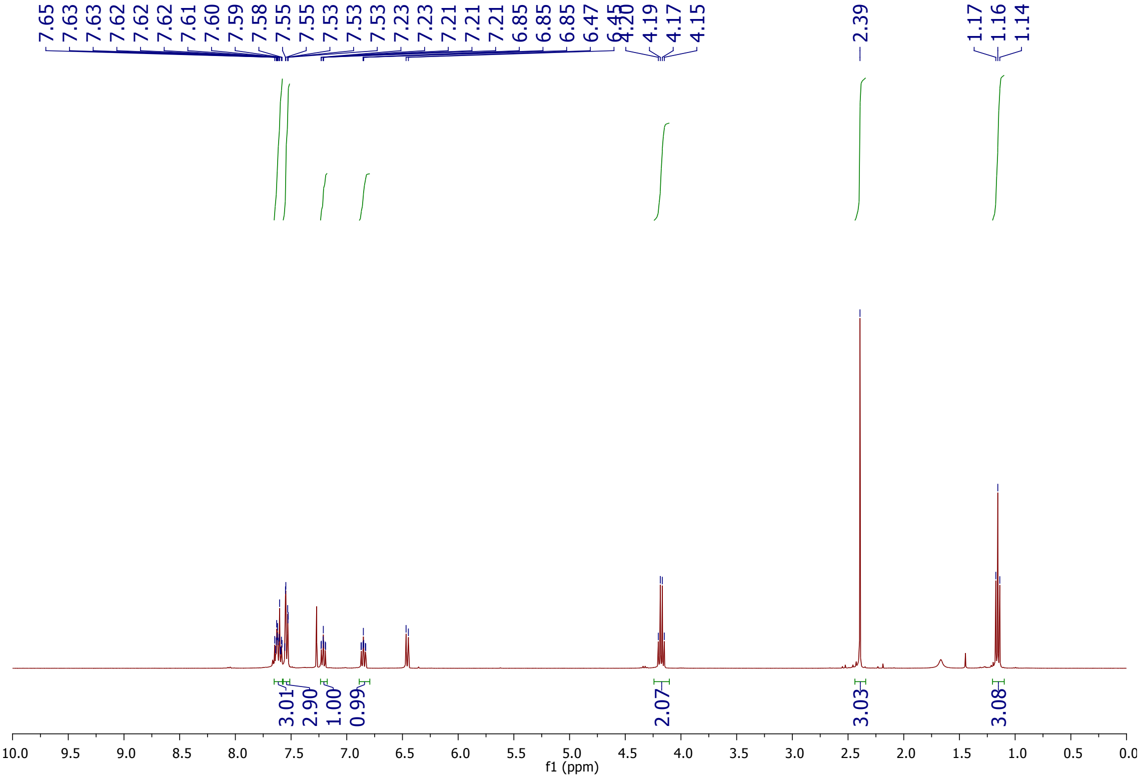

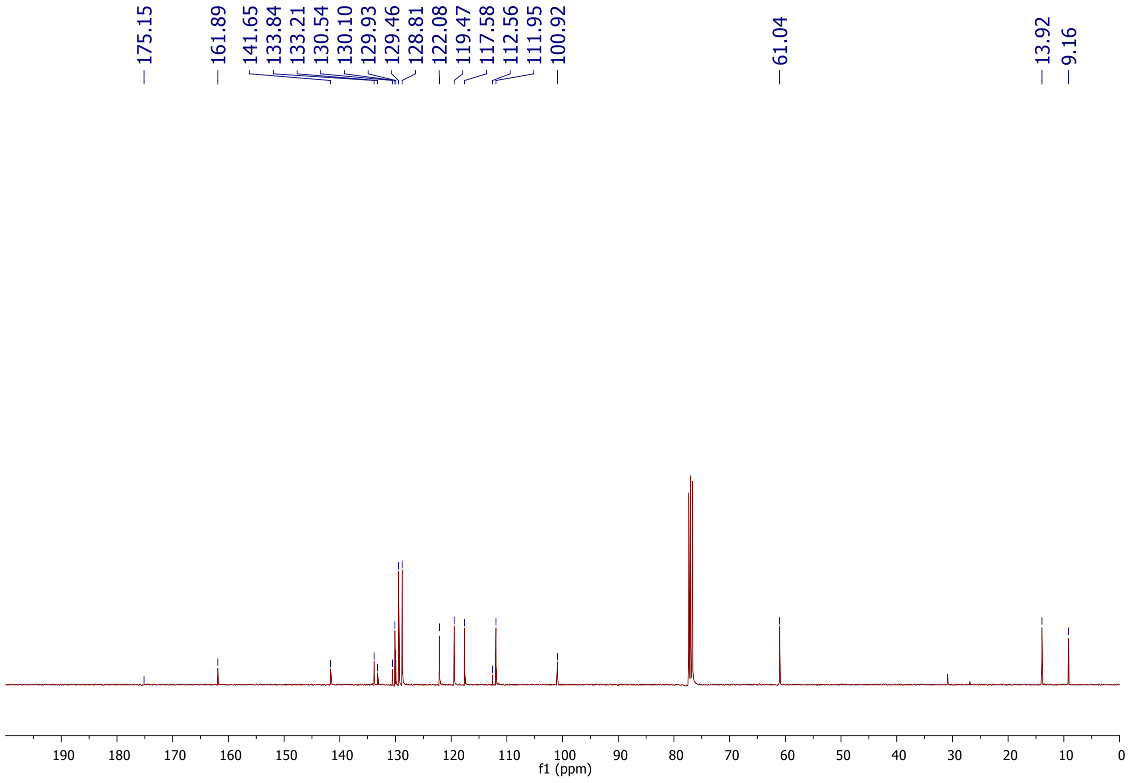

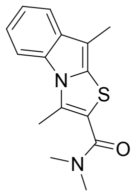

***N*,*N*,3,9-tetramethylthiazolo[3,2-*a*]indole-2-carboxamide 3h**

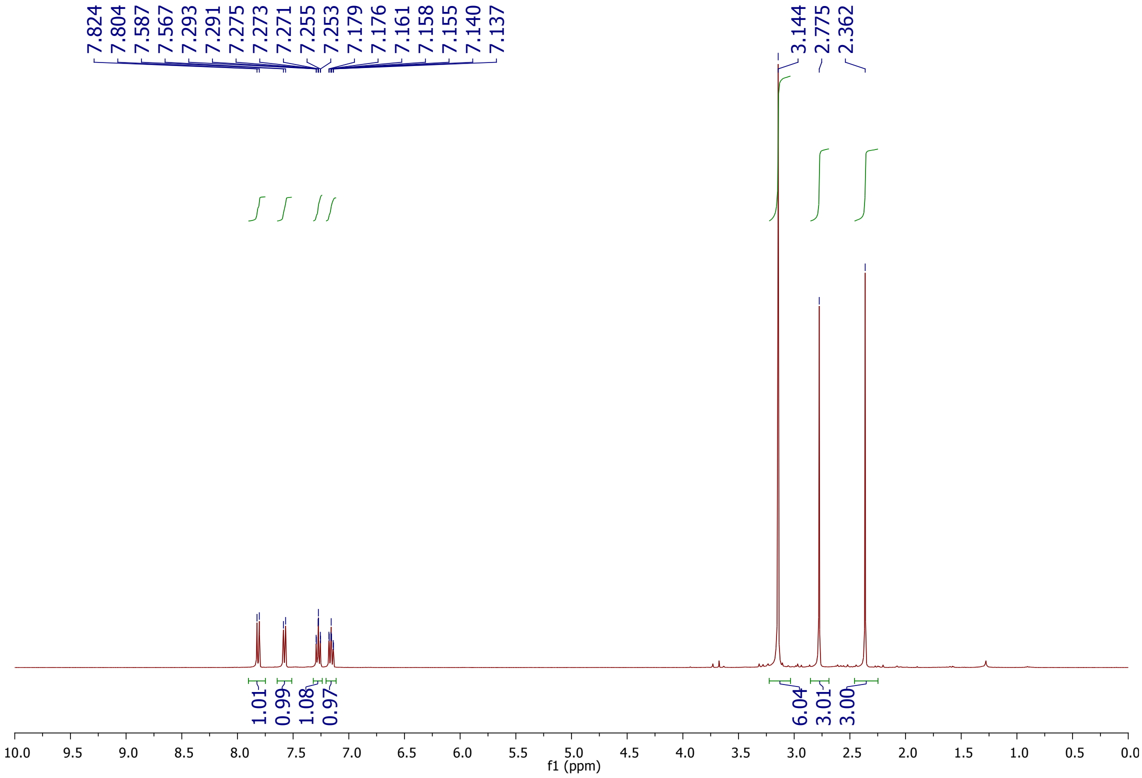

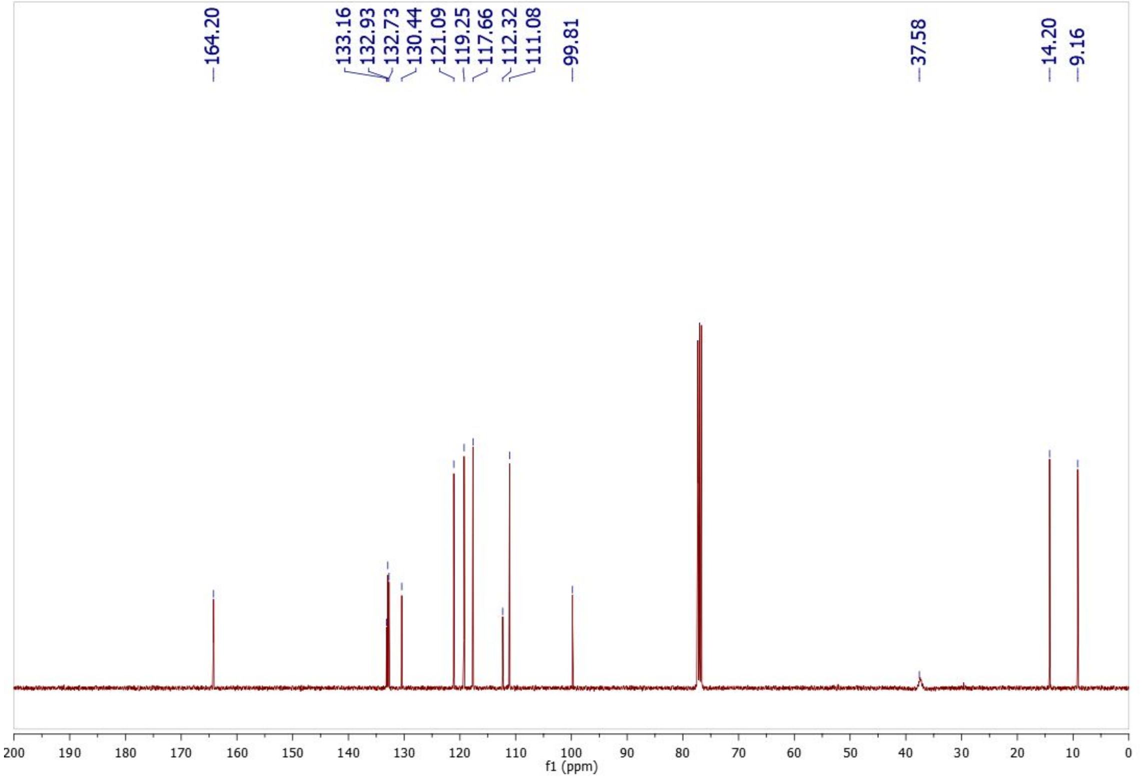

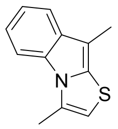

3,9-Dimethylthiazolo[3,2-*a*]indole 3i.

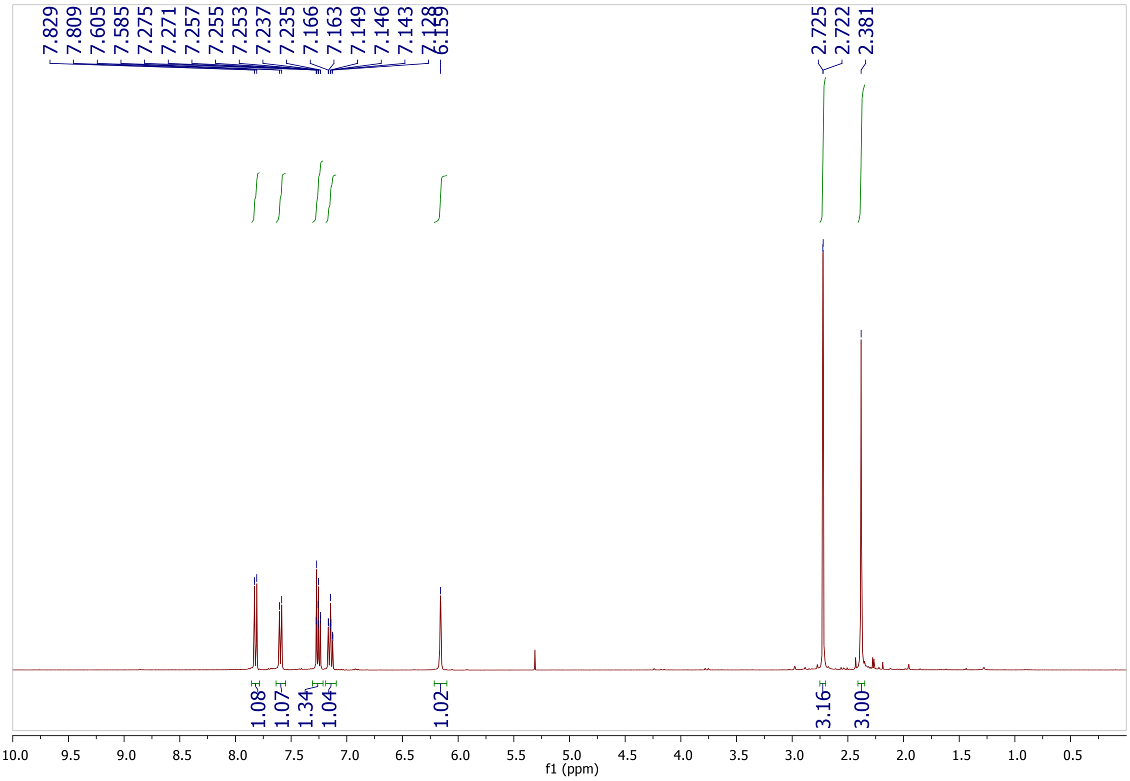

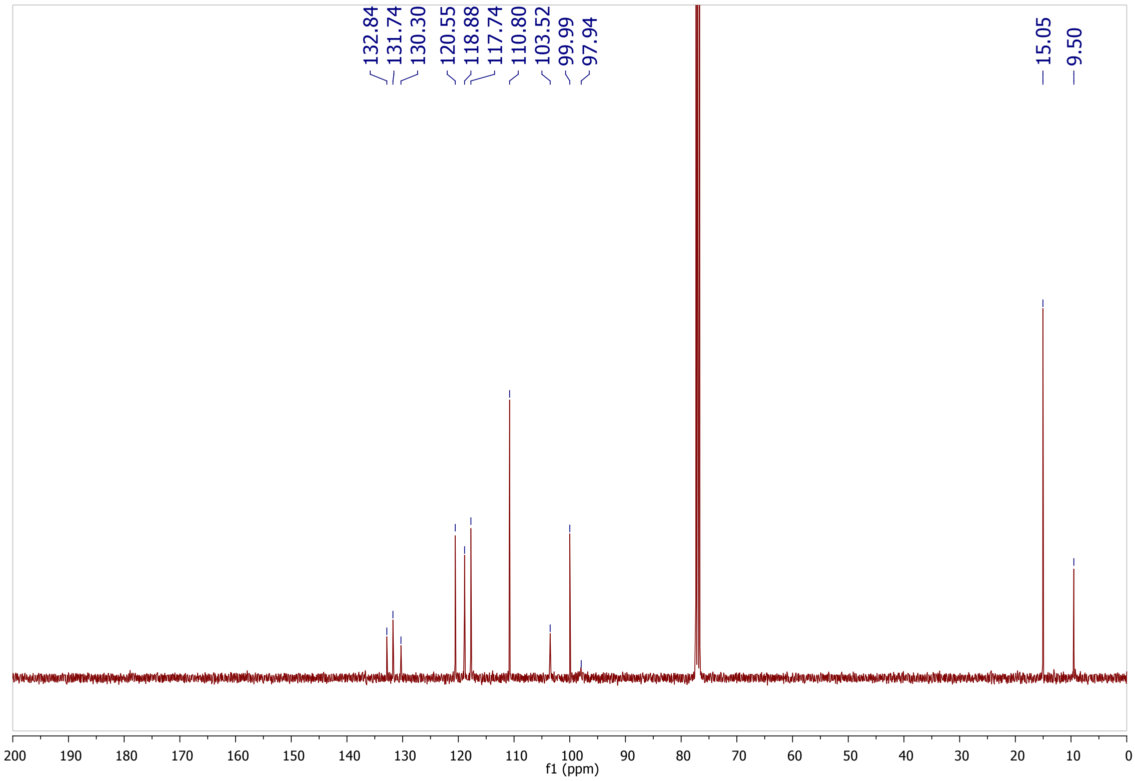

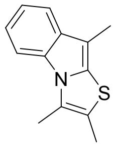

2,3,9-Trimethylthiazolo[3,2-*a*]indole 3j .

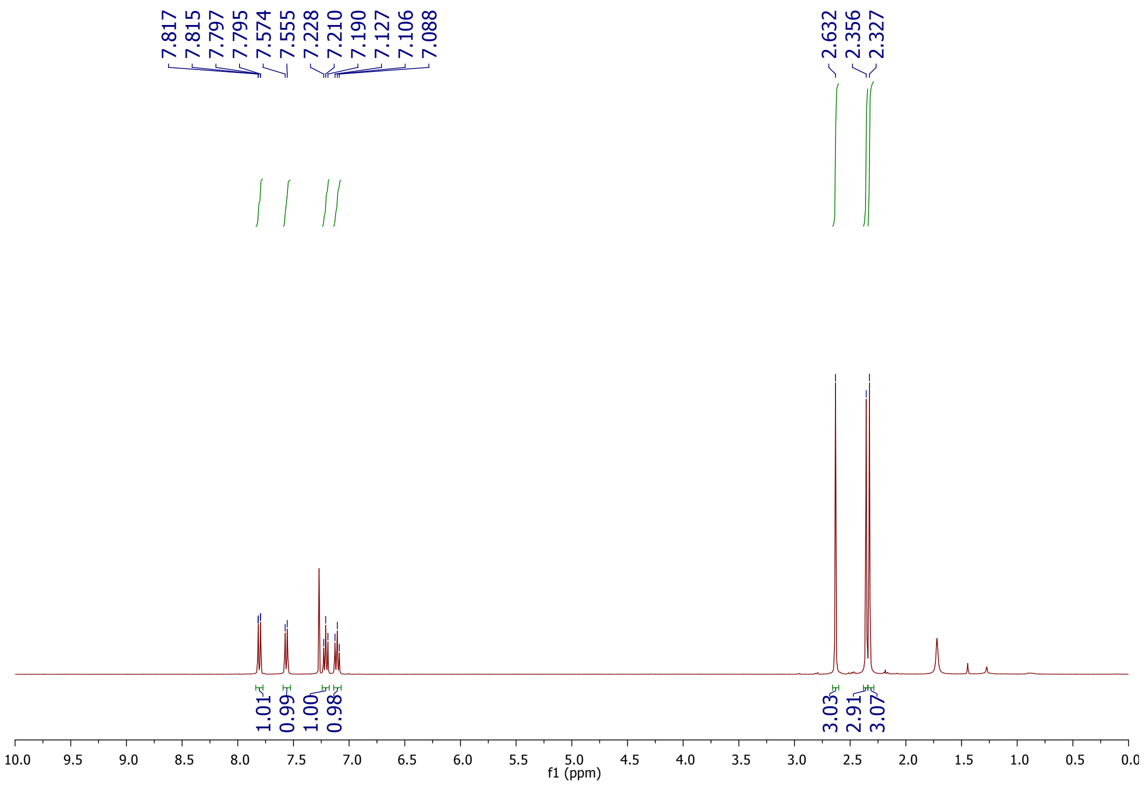
7.817
7.815
7.797
7.795
7.574
7.555
7.228
7.210
7.190
7.127
7.106
7.088
2.632
2.356
2.327
1.01
0.99
1.00
0.98
3.03
2.91
3.07
f1 (ppm)

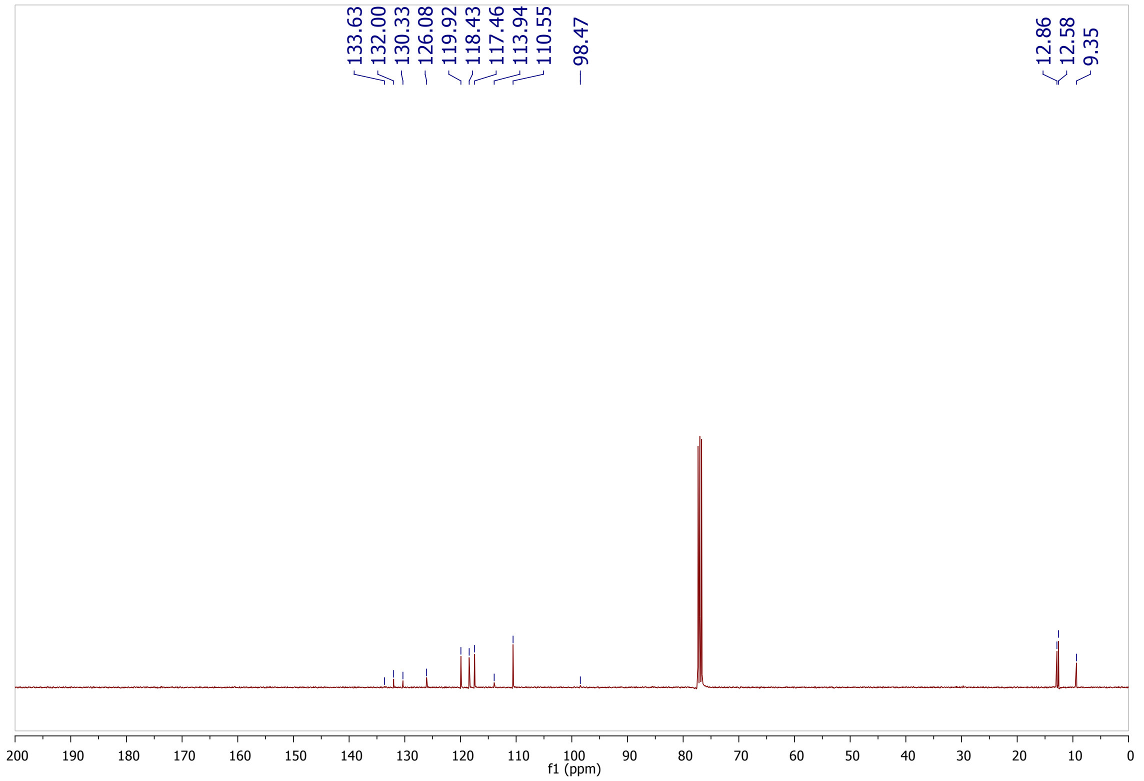
133.63
132.00
130.33
126.08
119.92
118.43
117.46
113.94
110.55
98.47
12.86
12.58
9.35
f1 (ppm)

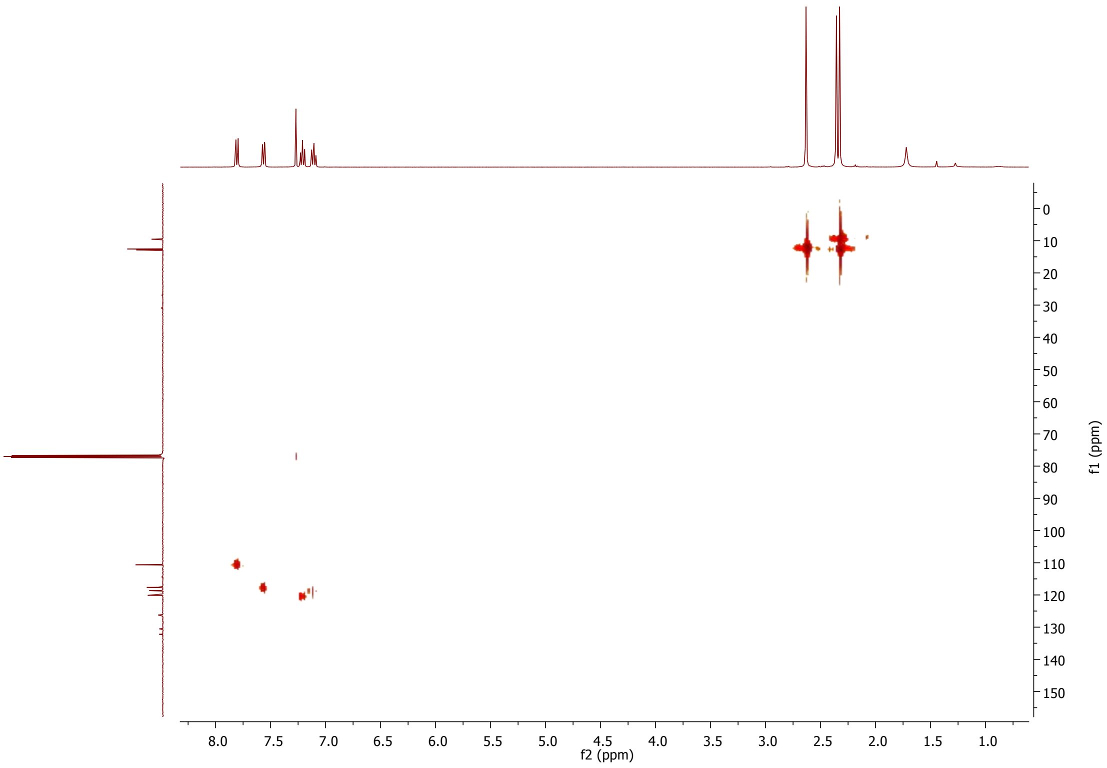
f1 (ppm)
f2 (ppm)
8.0 7.5 7.0 6.5 6.0 5.5 5.0 4.5 4.0 3.5 3.0 2.5 2.0 1.5 1.0
0 10 20 30 40 50 60 70 80 90 100 110 120 130 140 150

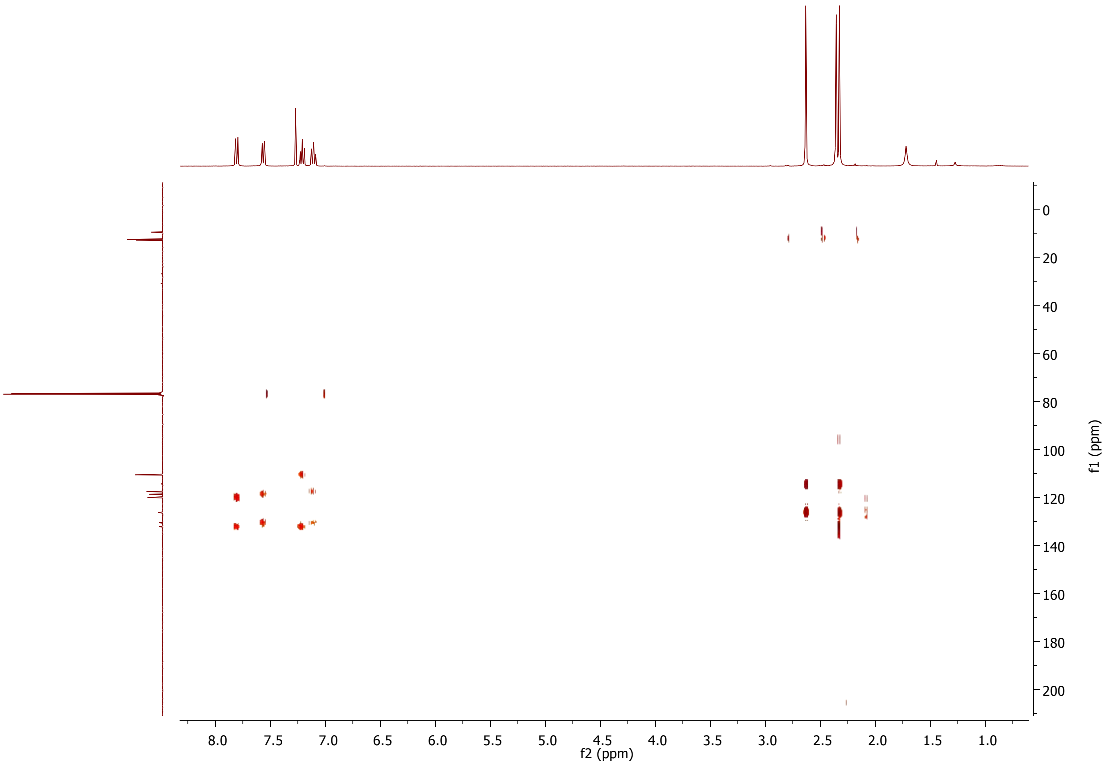
f1 (ppm)
f2 (ppm)
8.0 7.5 7.0 6.5 6.0 5.5 5.0 4.5 4.0 3.5 3.0 2.5 2.0 1.5 1.0
0 20 40 60 80 100 120 140 160 180 200

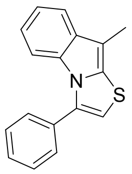

9-Methyl-3-phenylthiazolo[3,2-*a*]indole 3k.

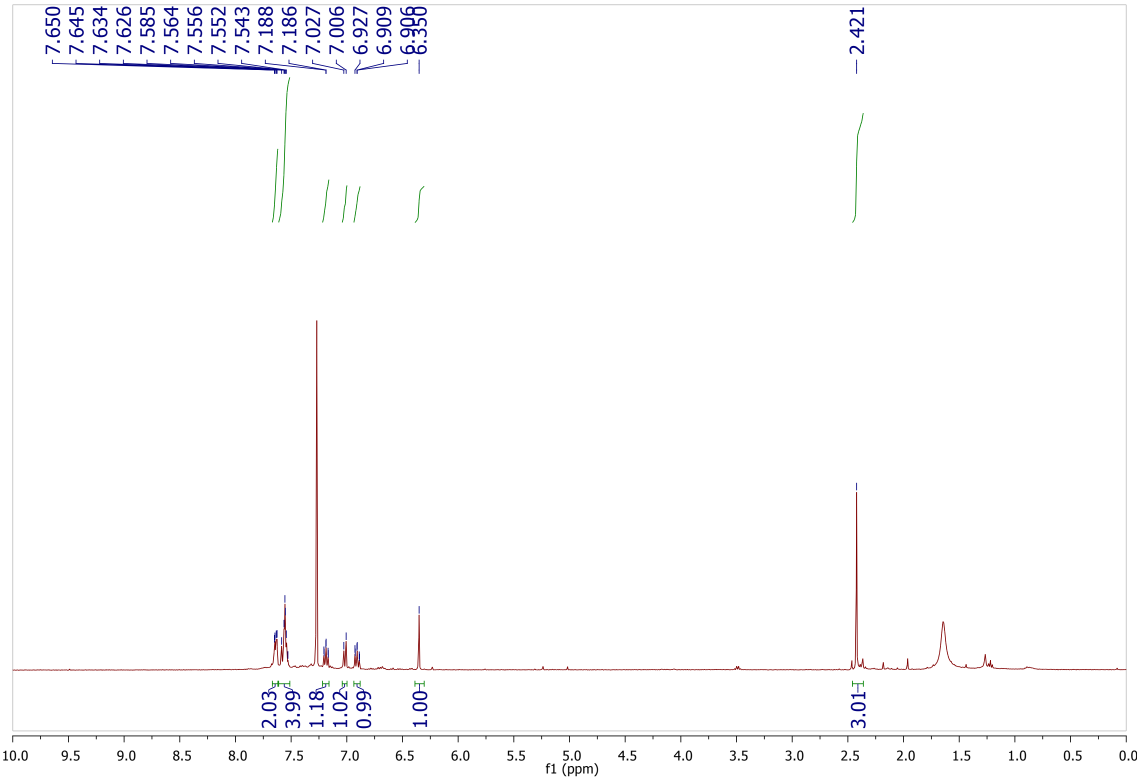

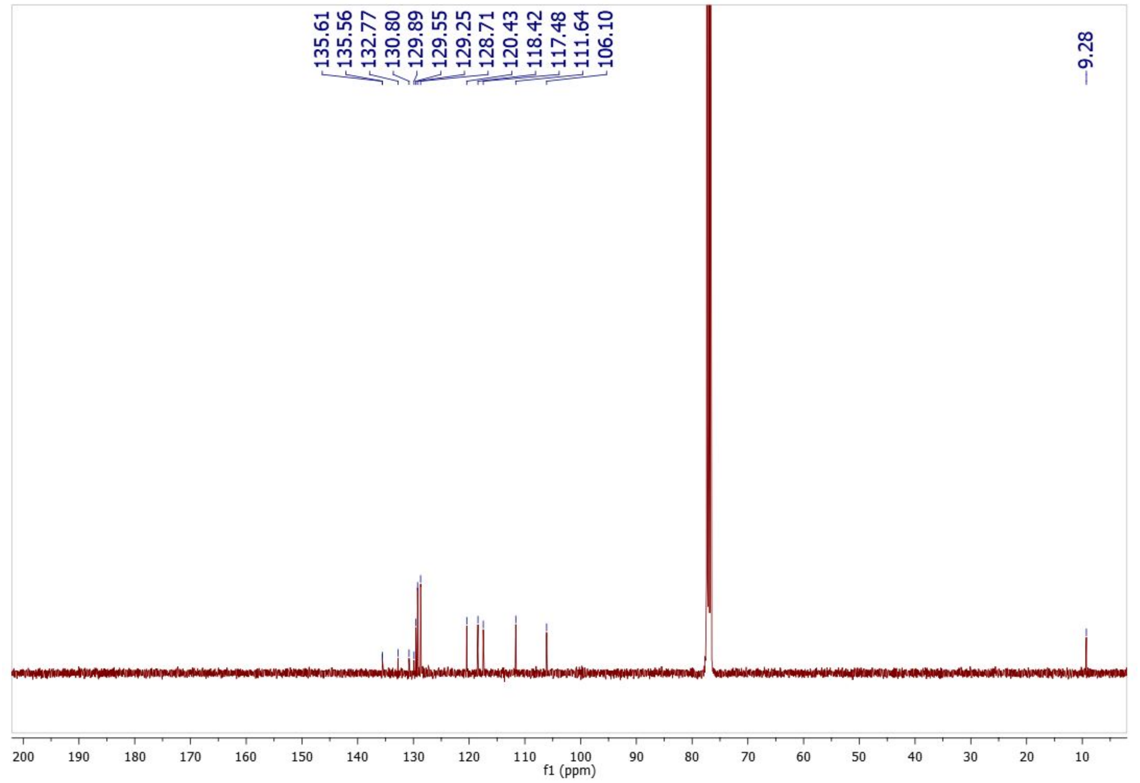

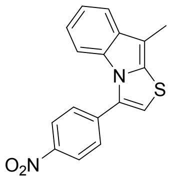

9-Methyl-3-(4-nitrophenyl)thiazolo[3,2-*a*]indole 3l.

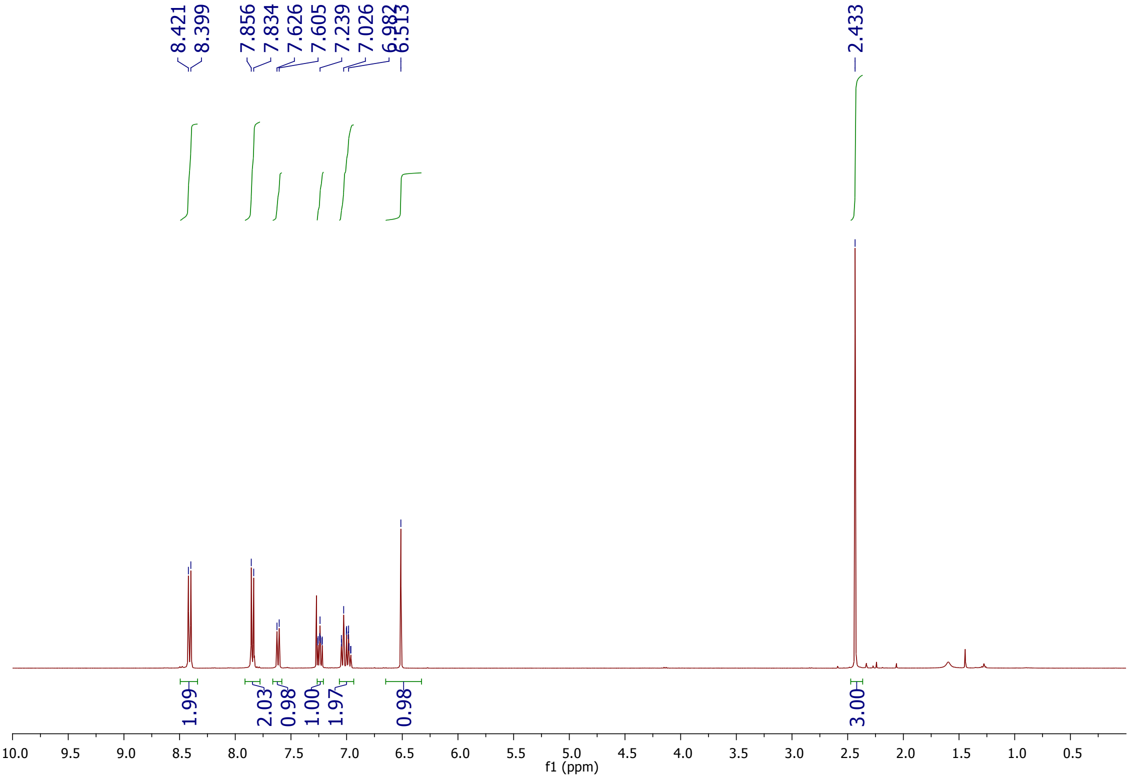

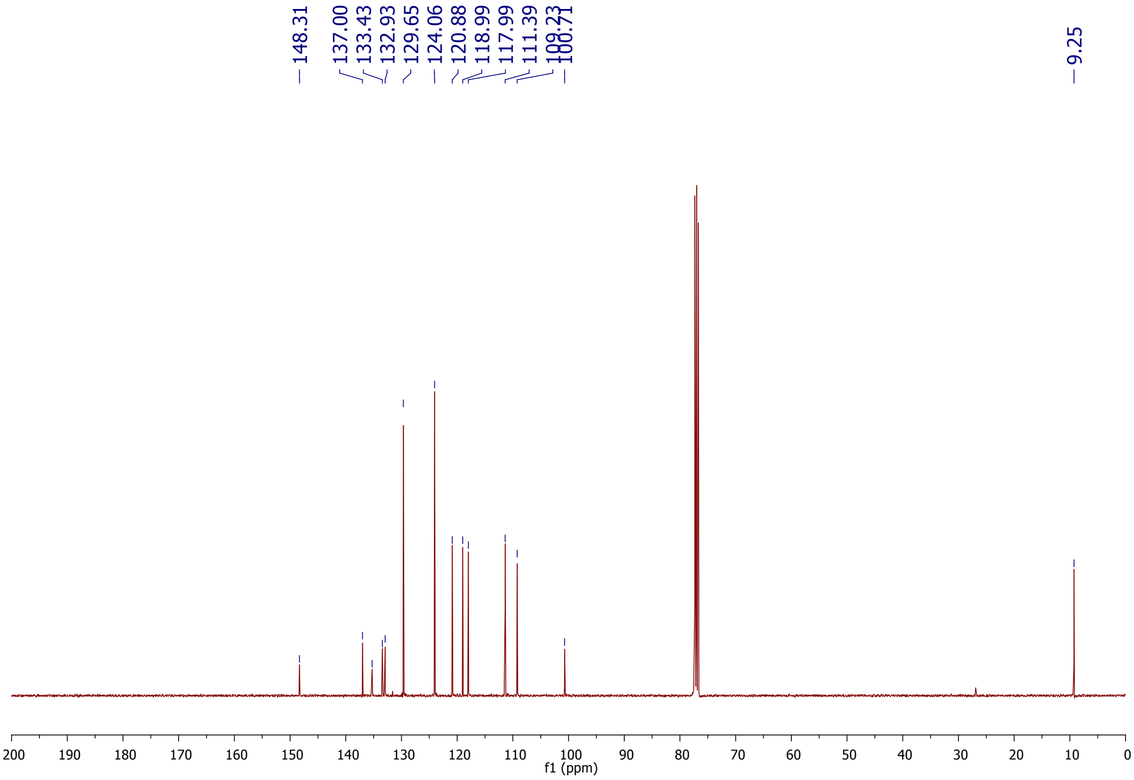

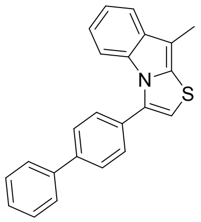

3-([1,1'-biphenyl]-4-yl)-9-methylthiazolo[3,2-*a*]indole 3m.

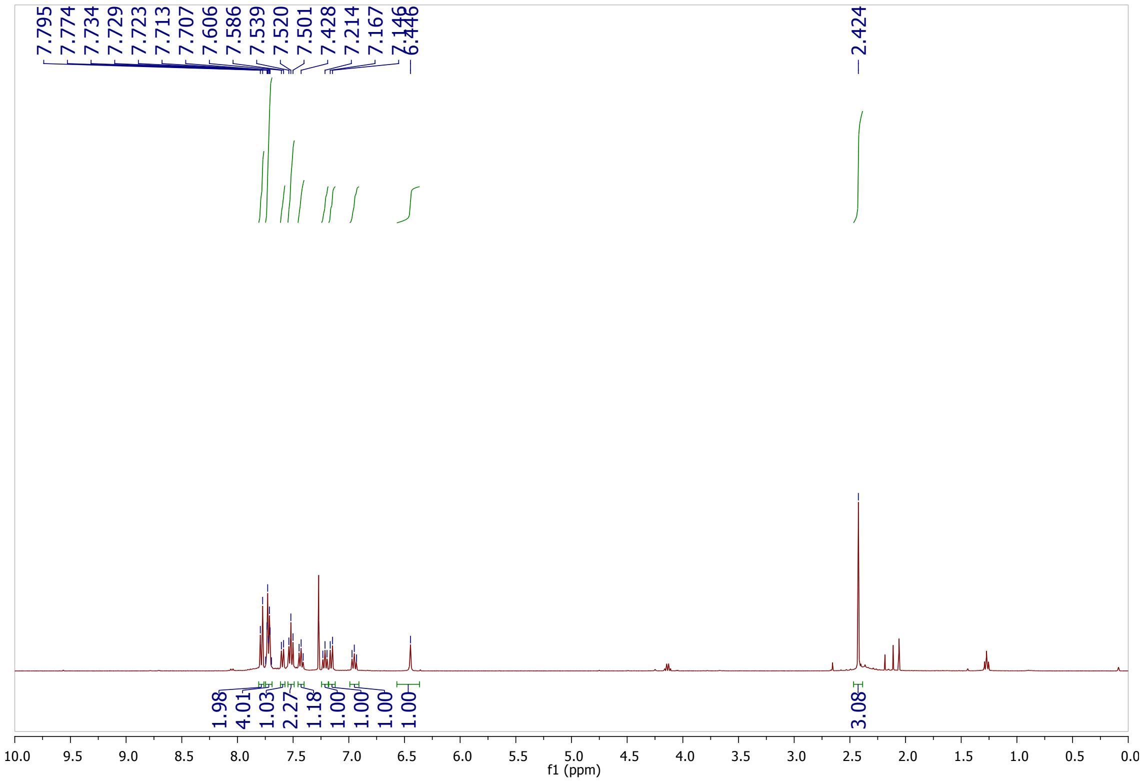

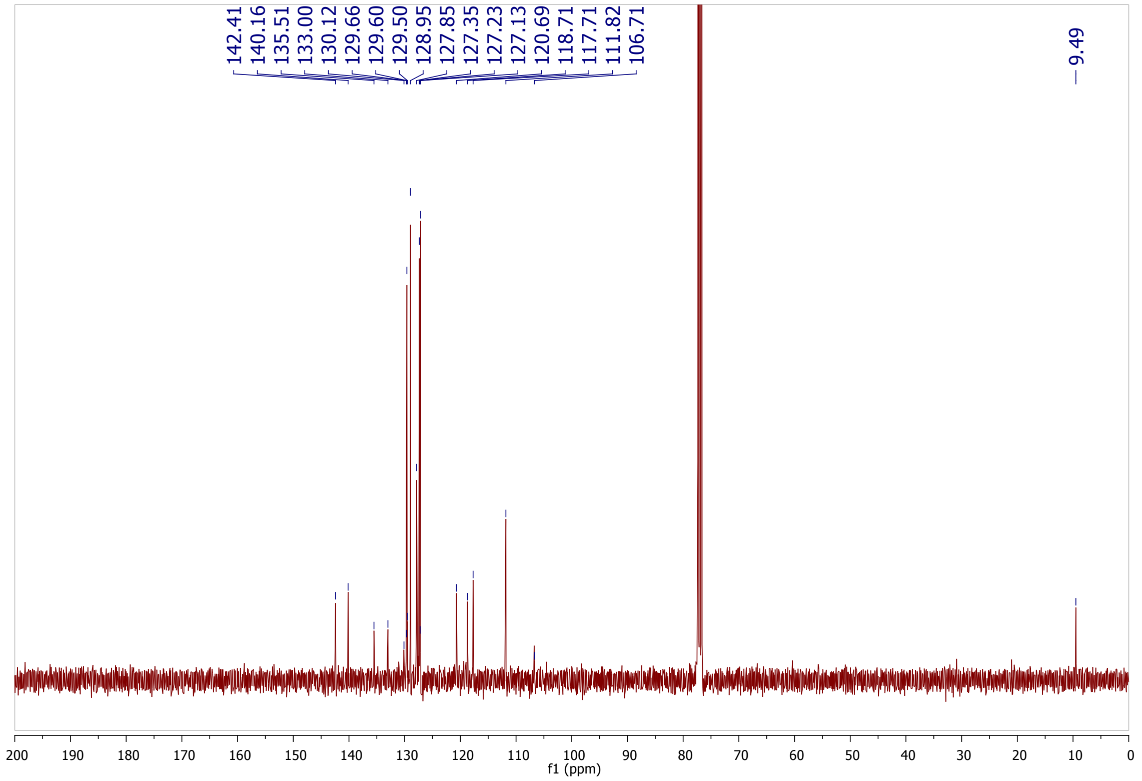

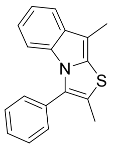

2,9-Dimethyl-3-phenylthiazolo[3,2-*a*]indole 3n.

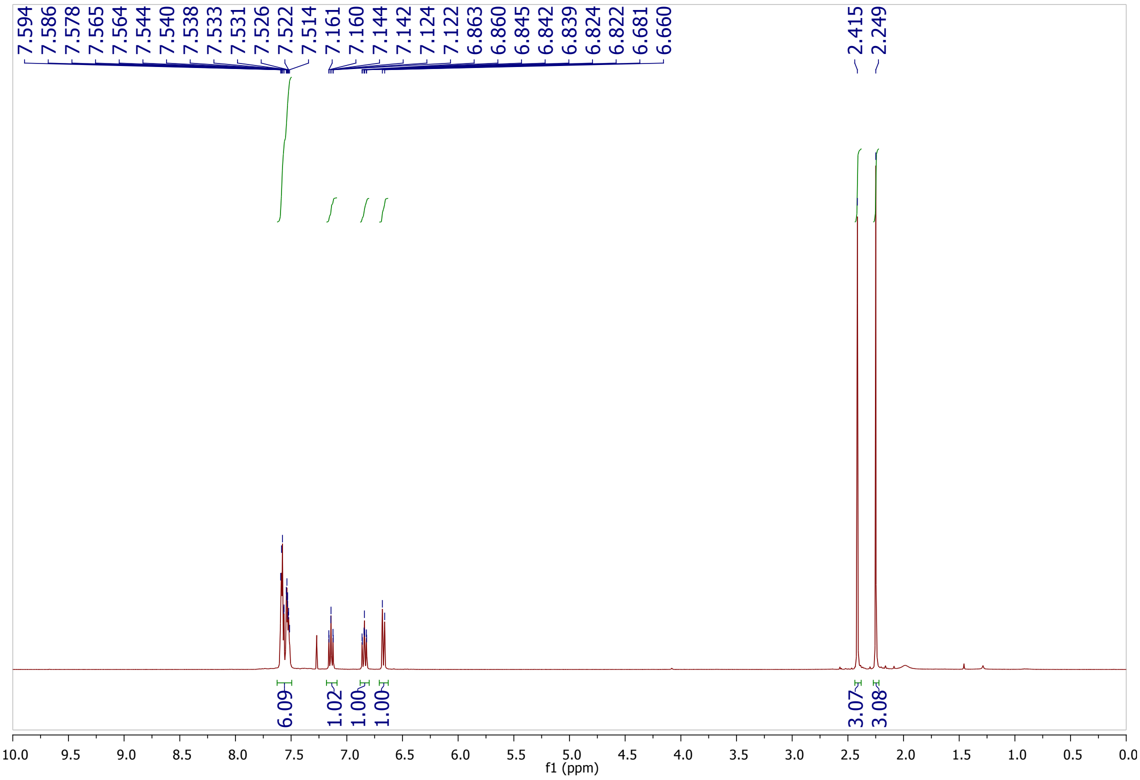

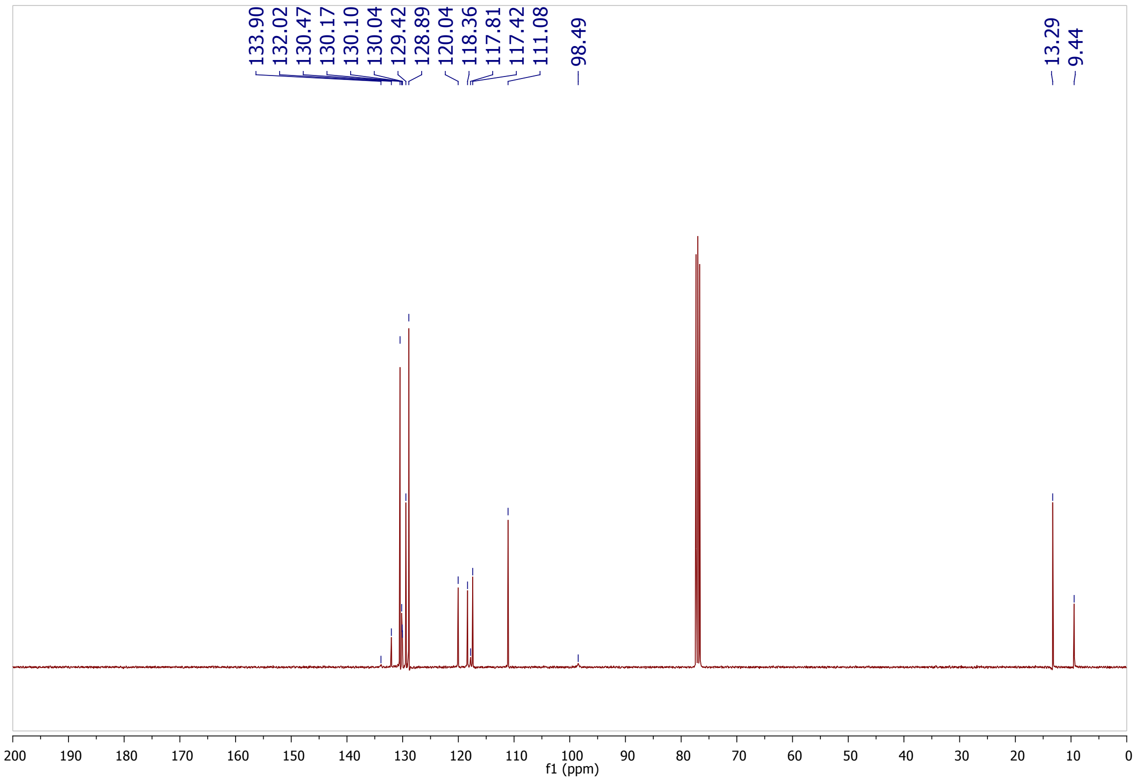

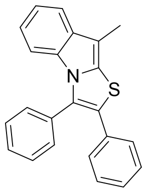

9-Methyl-2,3-diphenylthiazolo[3,2-*a*]indole 3o

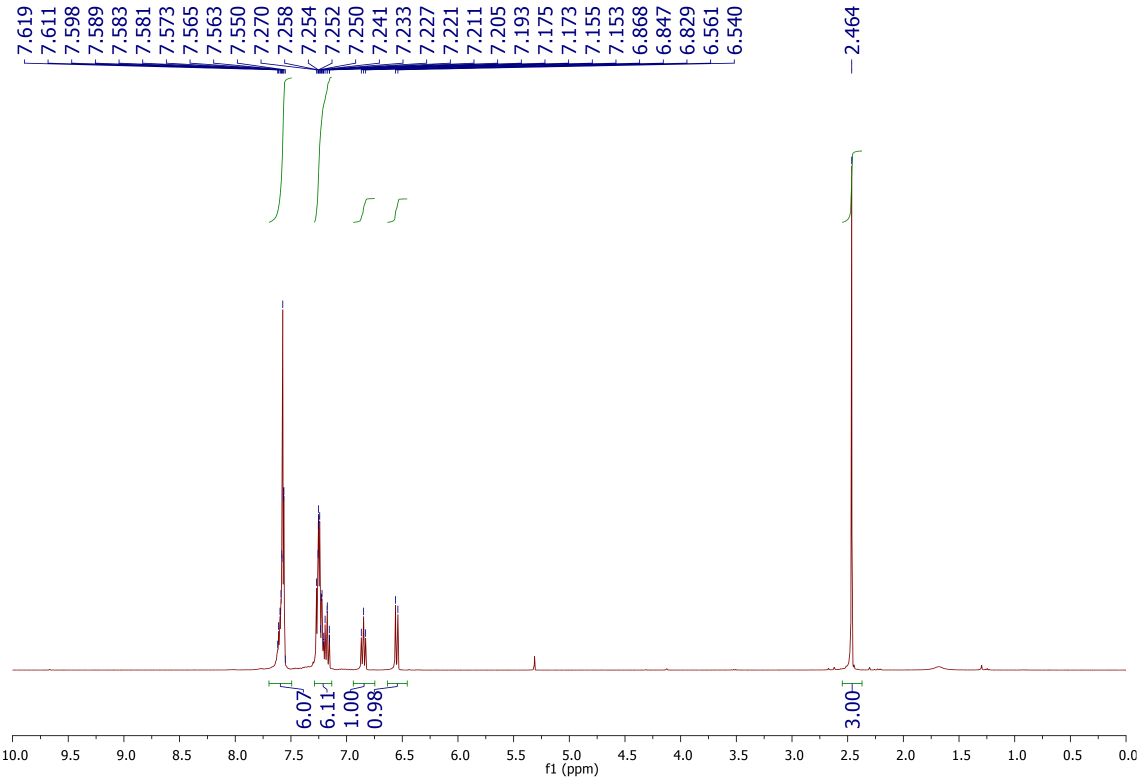
7.619
7.611
7.598
7.589
7.583
7.581
7.573
7.565
7.563
7.550
7.270
7.258
7.254
7.252
7.250
7.241
7.233
7.227
7.221
7.211
7.205
7.193
7.175
7.173
7.155
7.153
6.868
6.847
6.829
6.561
6.540
2.464
6.07
6.11
1.00
0.98
3.00
10.0 9.5 9.0 8.5 8.0 7.5 7.0 6.5 6.0 5.5 5.0 4.5 4.0 3.5 3.0 2.5 2.0 1.5 1.0 0.5 0.0
f1 (ppm)

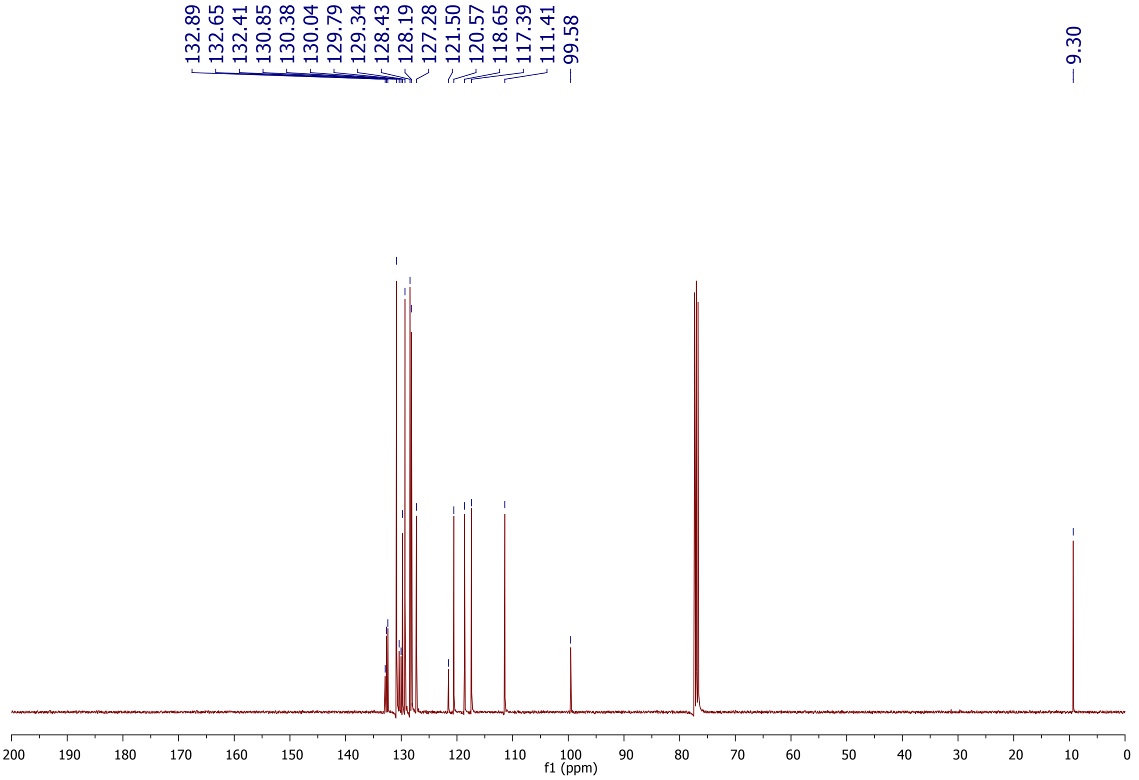
132.89
132.65
132.41
130.85
130.38
130.04
129.79
129.34
128.43
128.19
127.28
121.50
120.57
118.65
117.39
111.41
99.58
9.30
200 190 180 170 160 150 140 130 120 110 100 90 80 70 60 50 40 30 20 10 0
f1 (ppm)

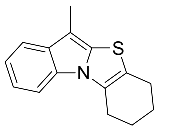

11-Methyl-6,7,8,9-tetrahydrobenzo[4,5]thiazolo[3,2-*a*]indole 3p

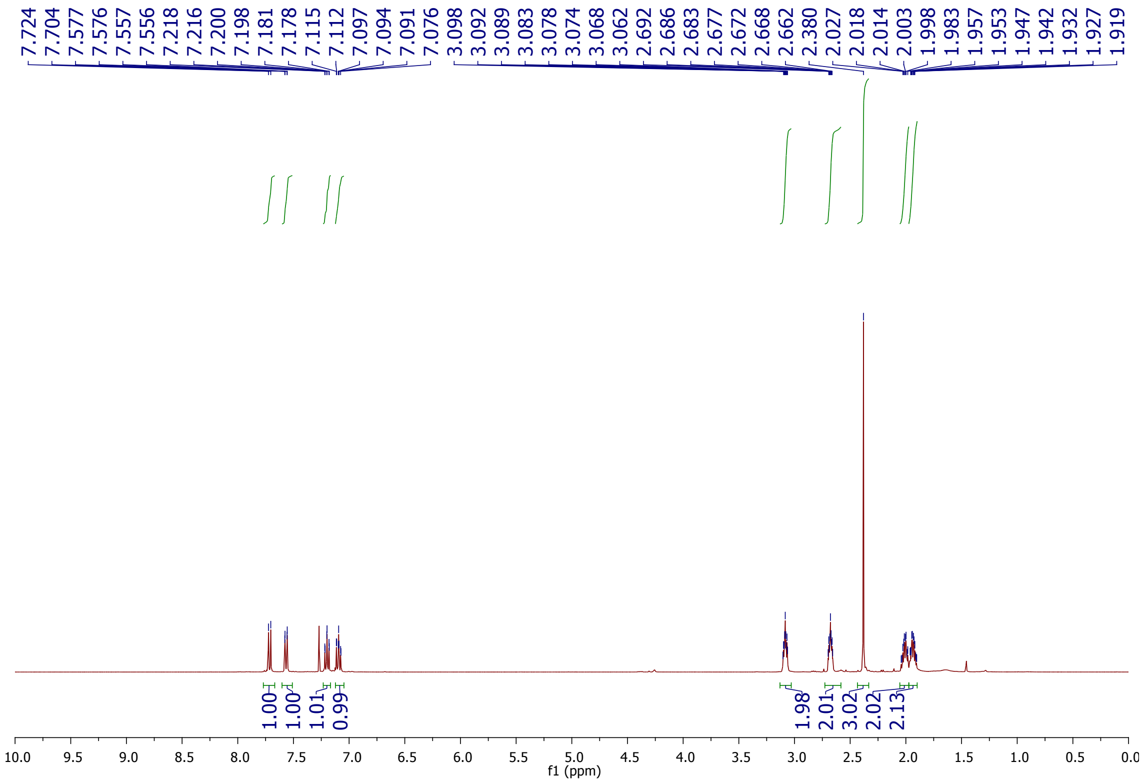

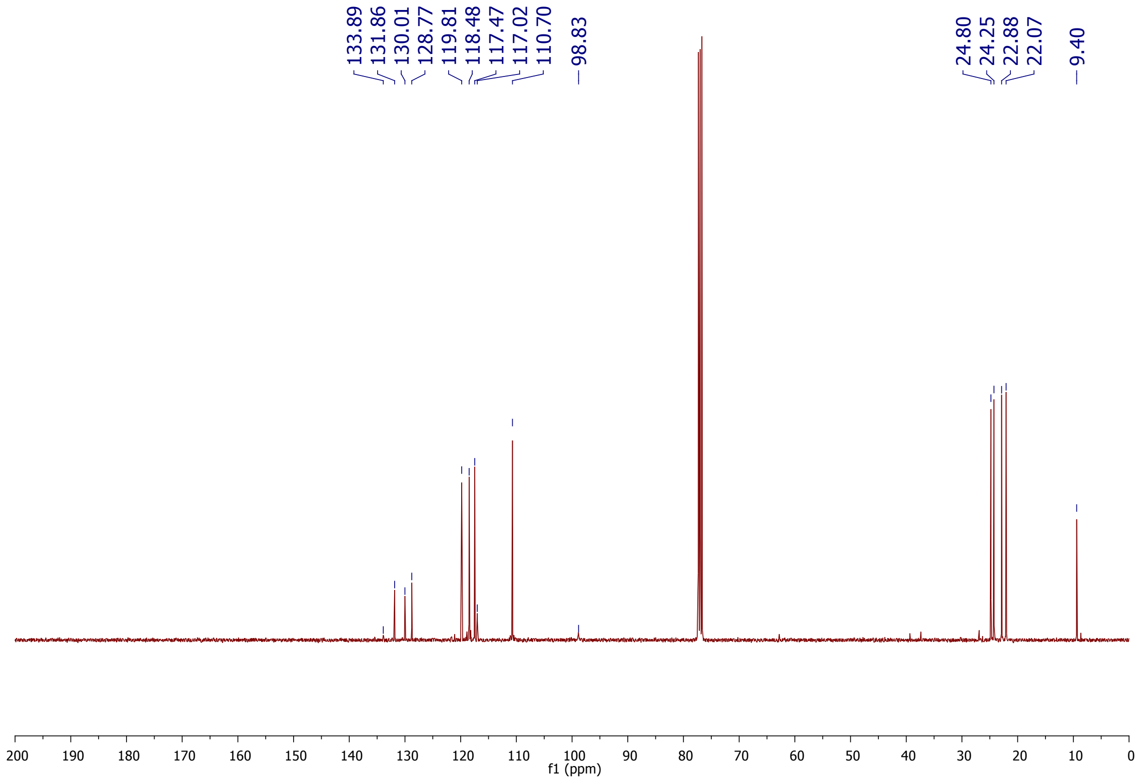

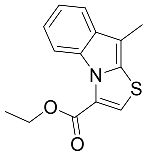

Ethyl 9-methylthiazolo[3,2-*a*]indole-3-carboxylate 3q.

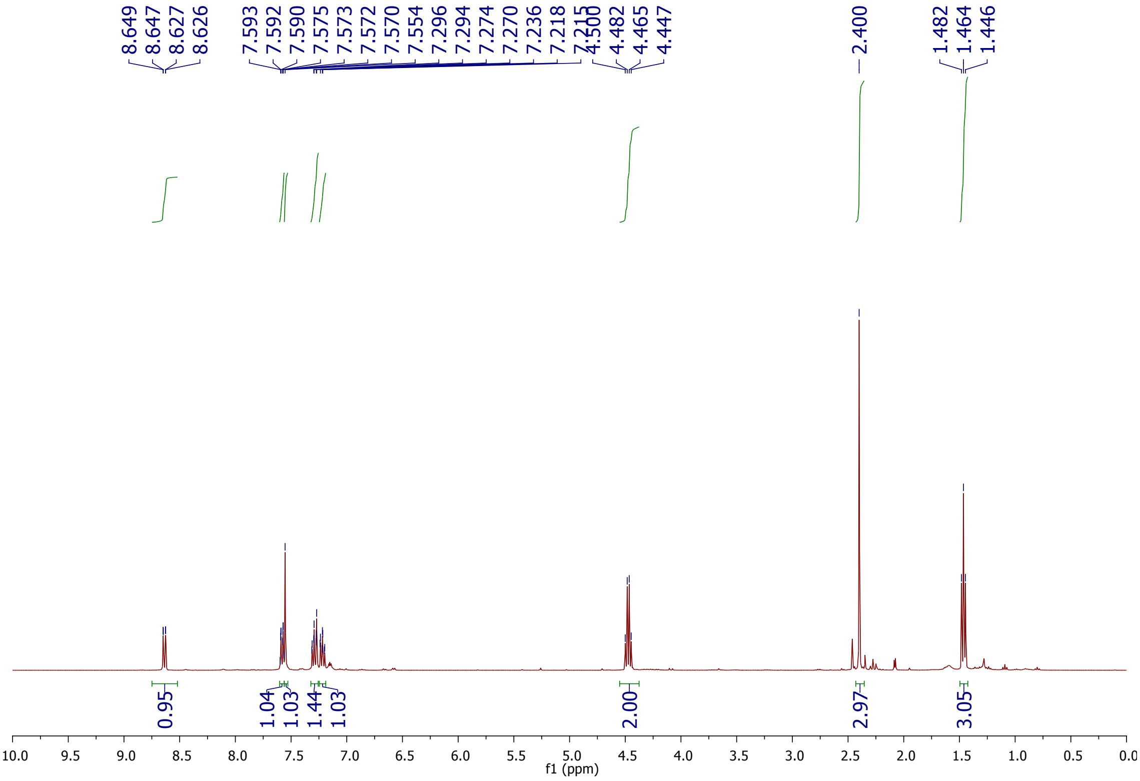

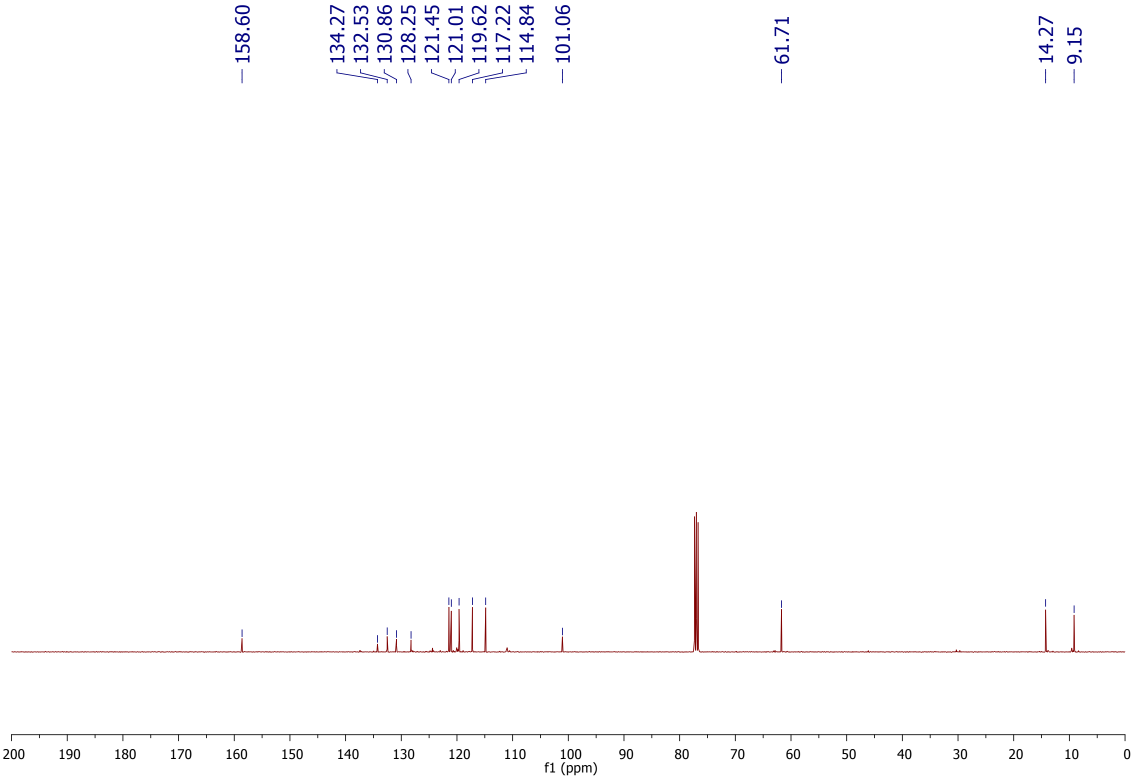

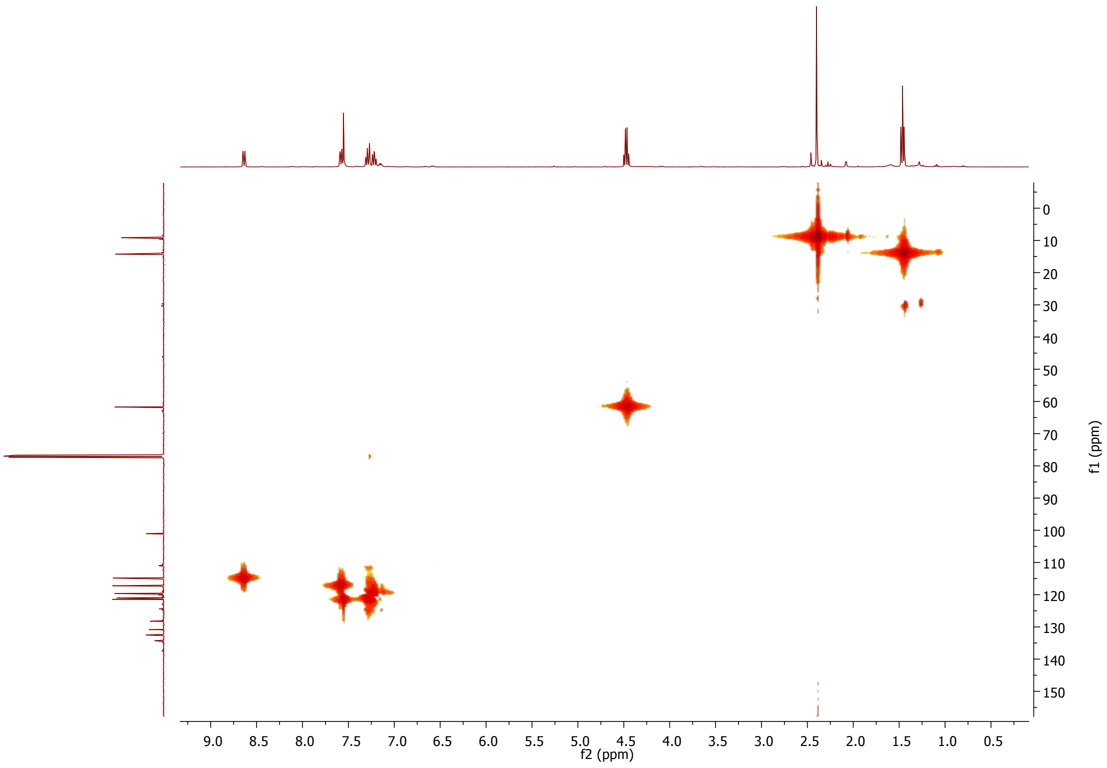
f1 (ppm)
f2 (ppm)

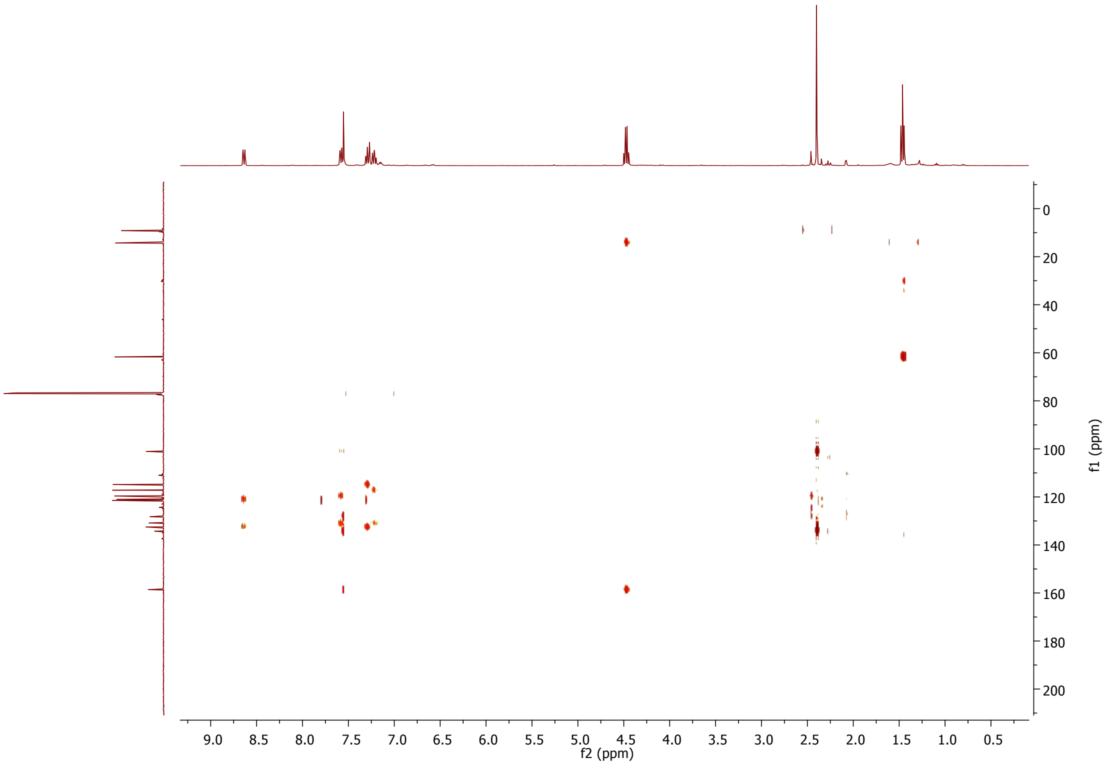
f1 (ppm)
f2 (ppm)

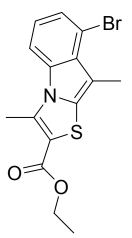

Ethyl 8-bromo-3,9-dimethylthiazolo[3,2-*a*]indole-2-carboxylate 3r.

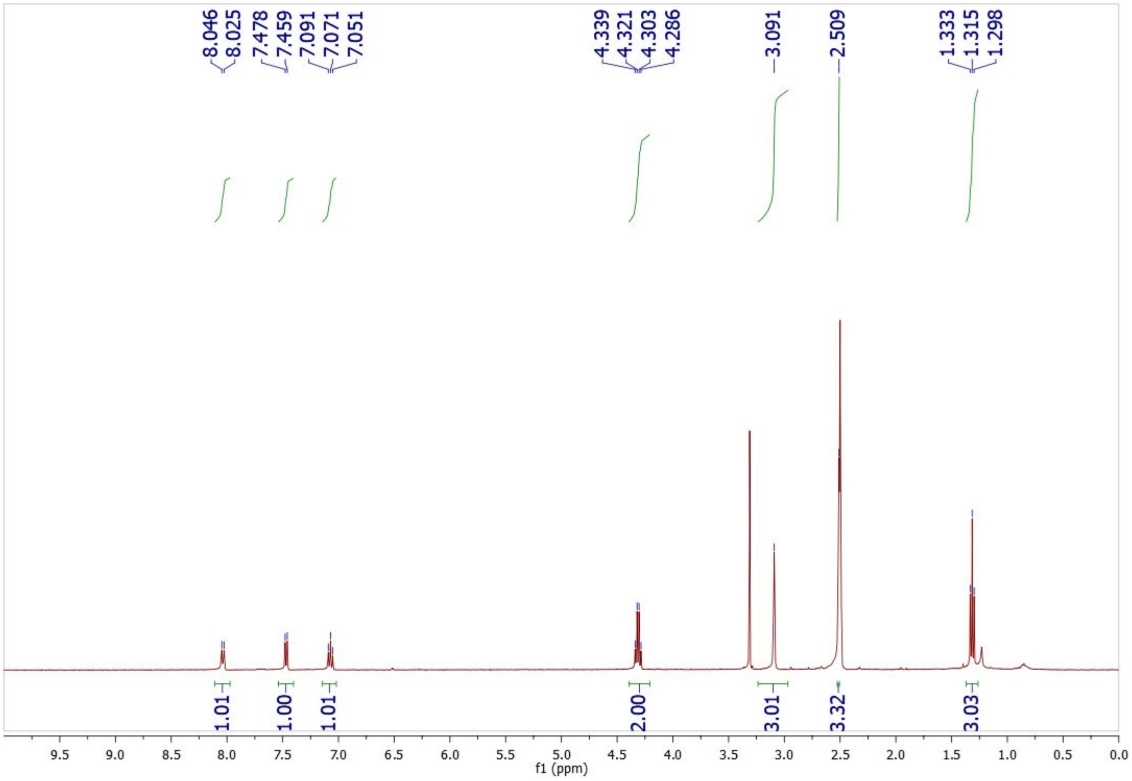

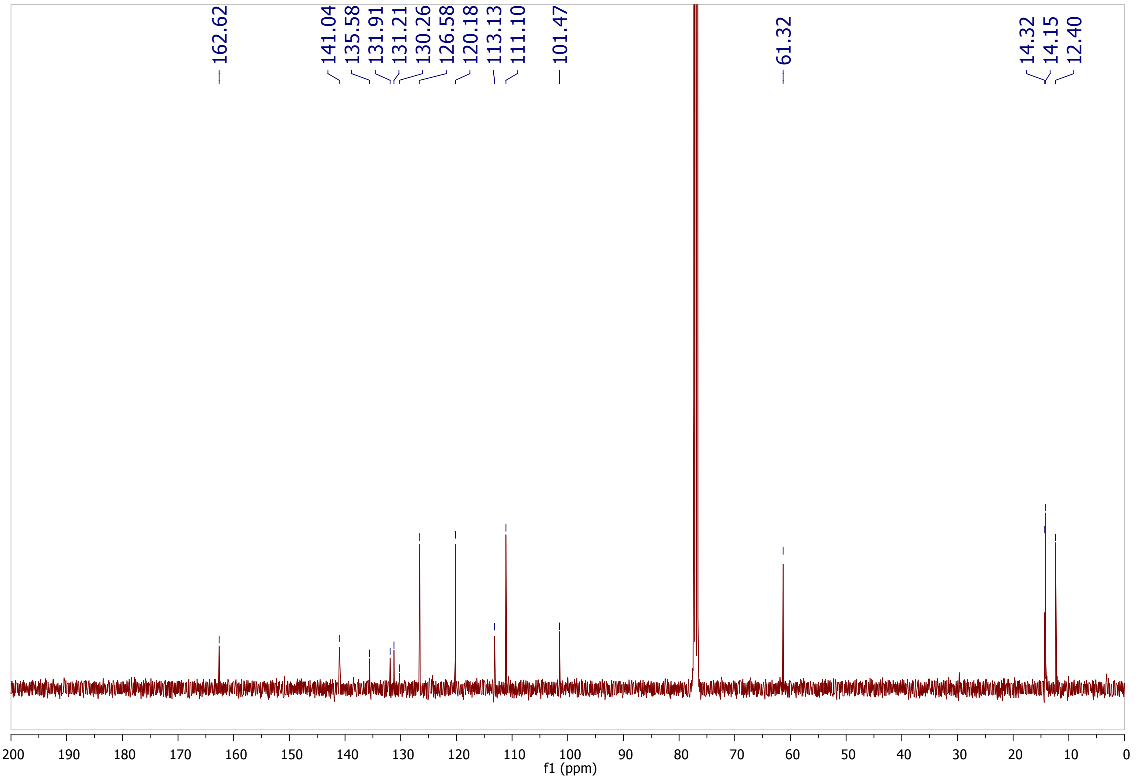

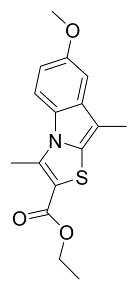

Ethyl 7-methoxy-3,9-dimethylthiazolo[3,2-*a*]indole-2-carboxylate 3s.

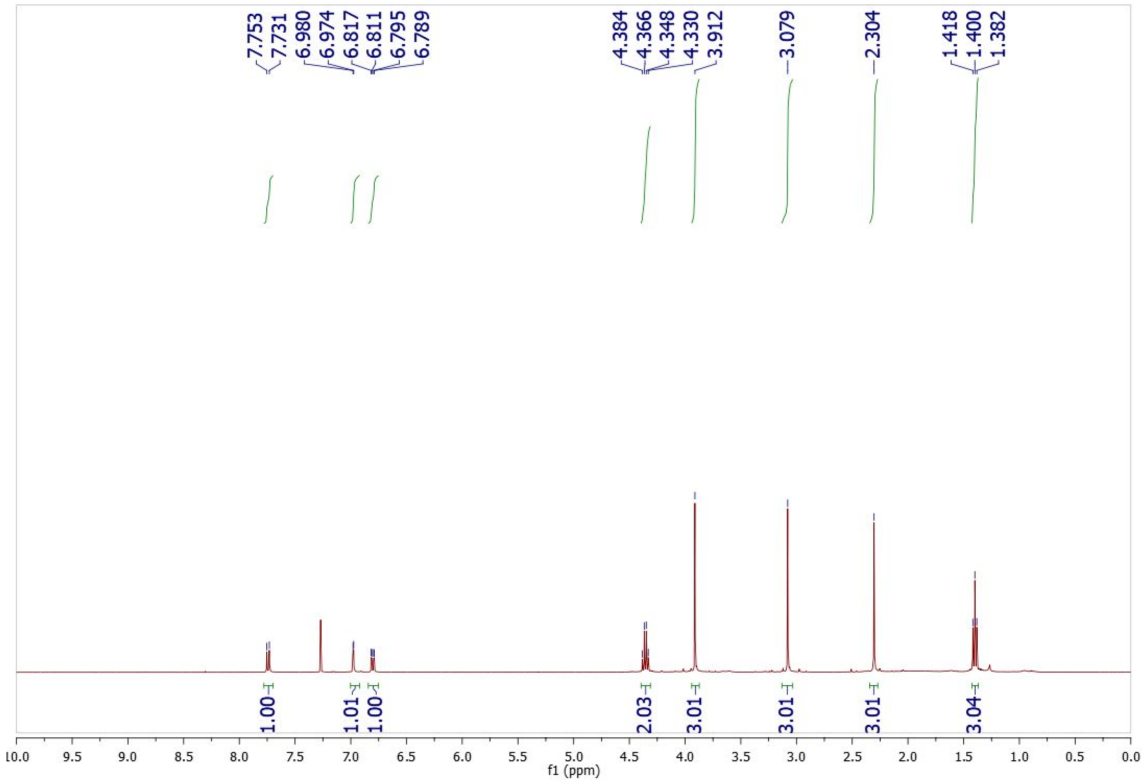

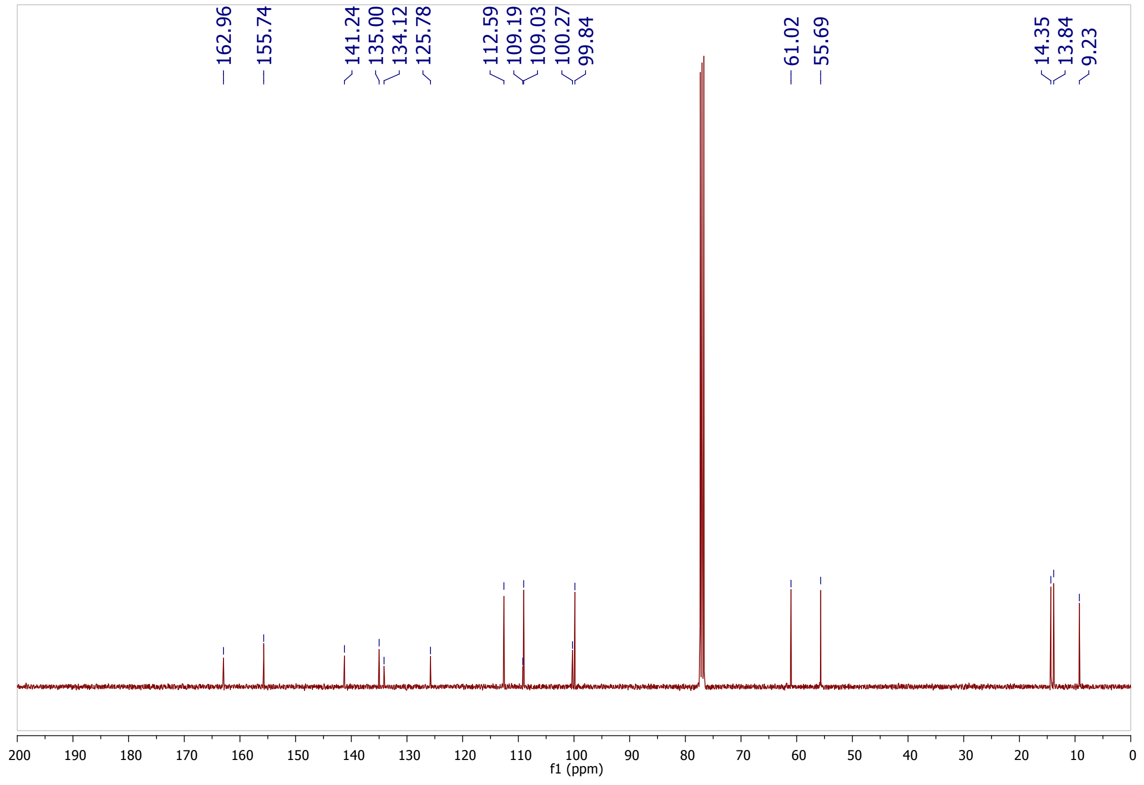

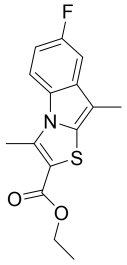

Ethyl 7-Fluoro-3,9-dimethylthiazolo[3,2-*a*]indole-2-carboxylate 3t.

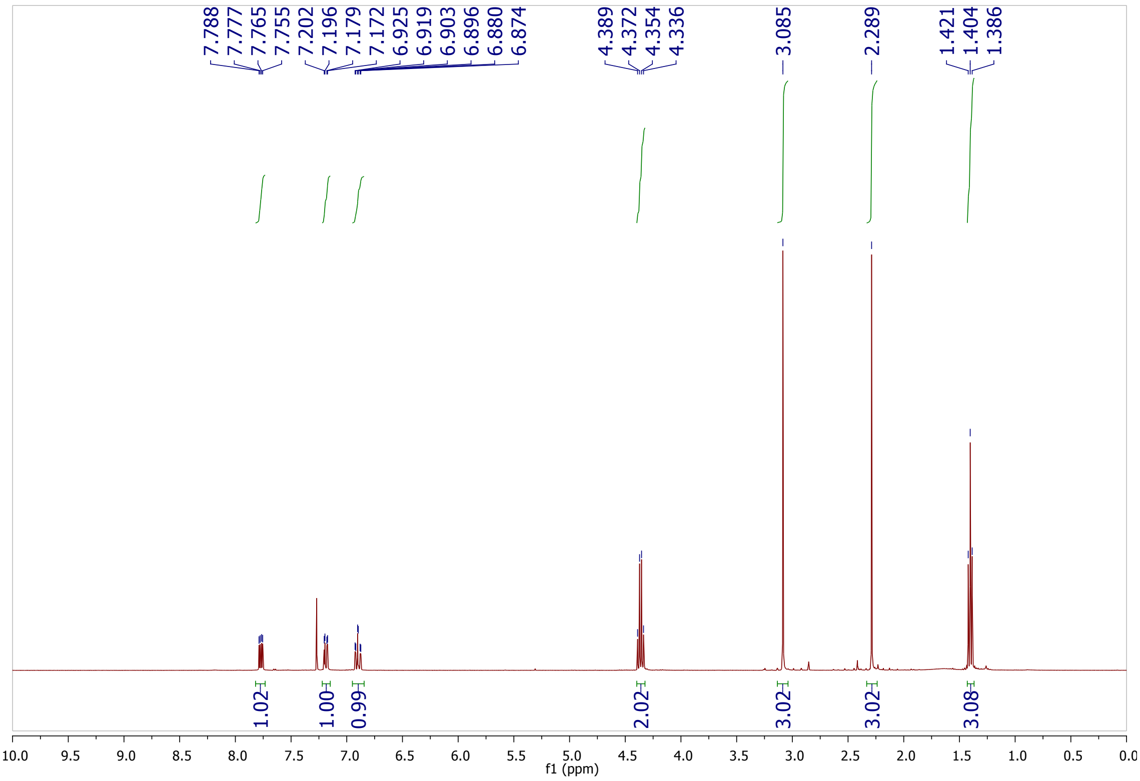

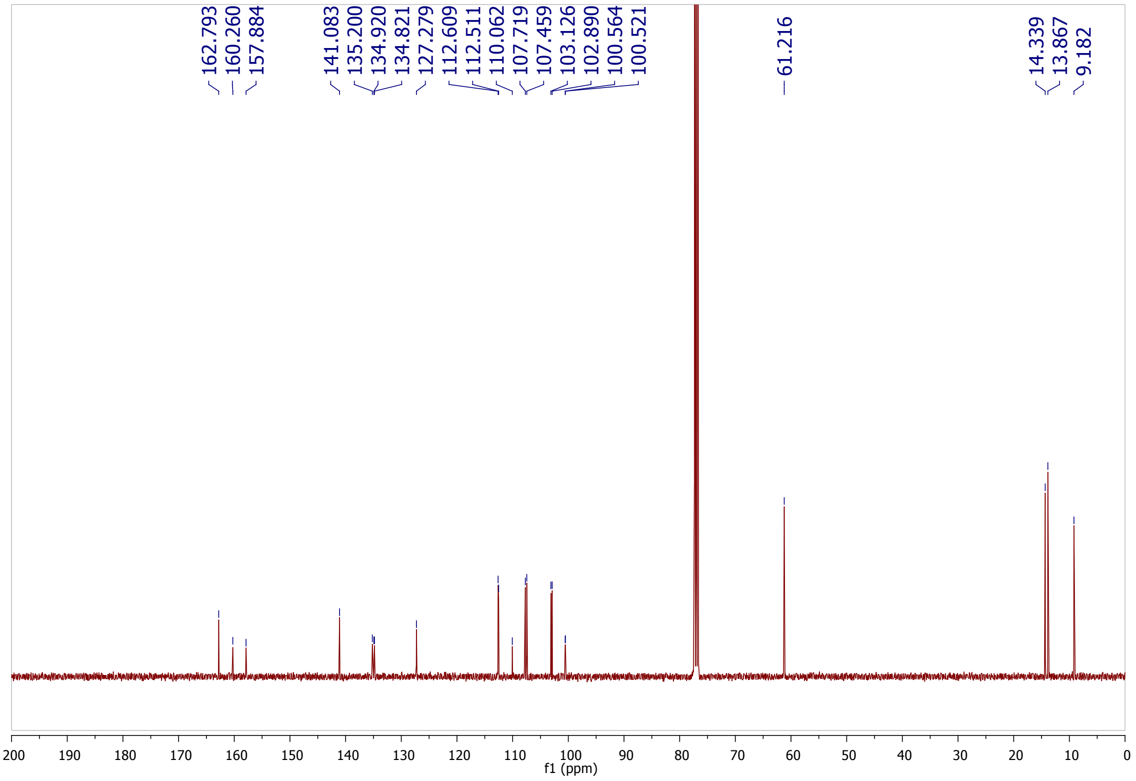

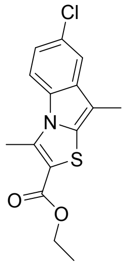

Ethyl 7-Chloro-3,9-dimethylthiazolo[3,2-*a*]indole-2-carboxylate 3u.

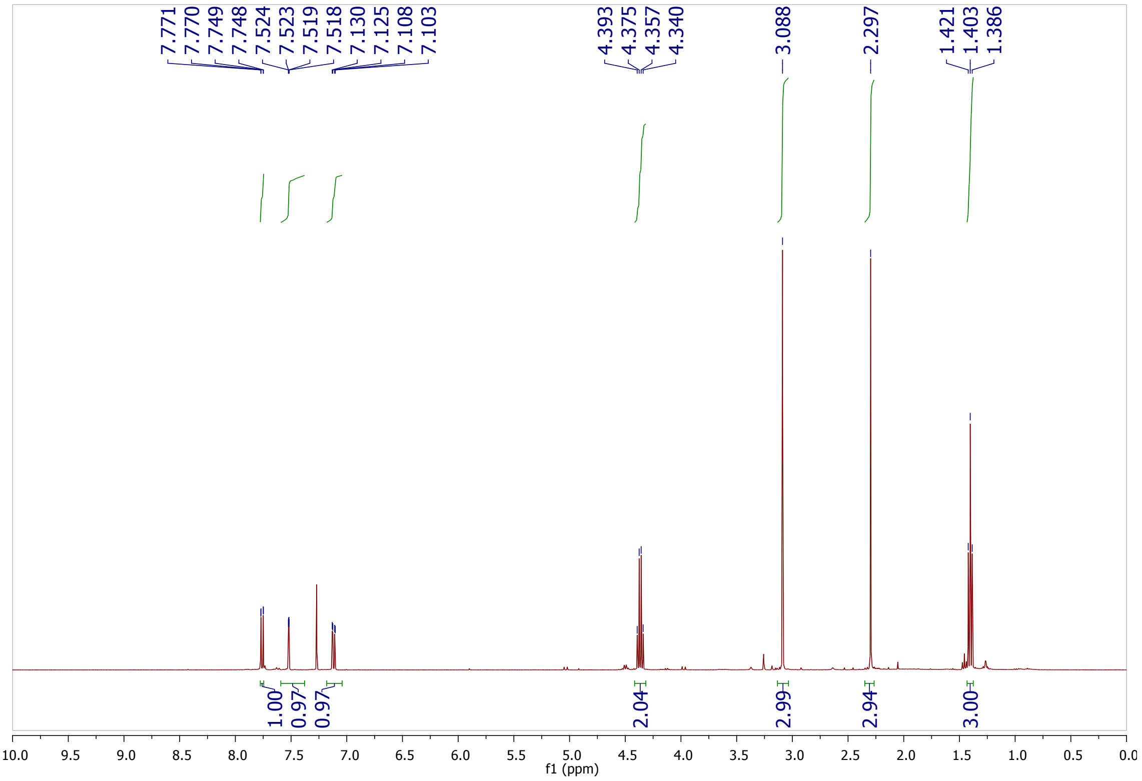

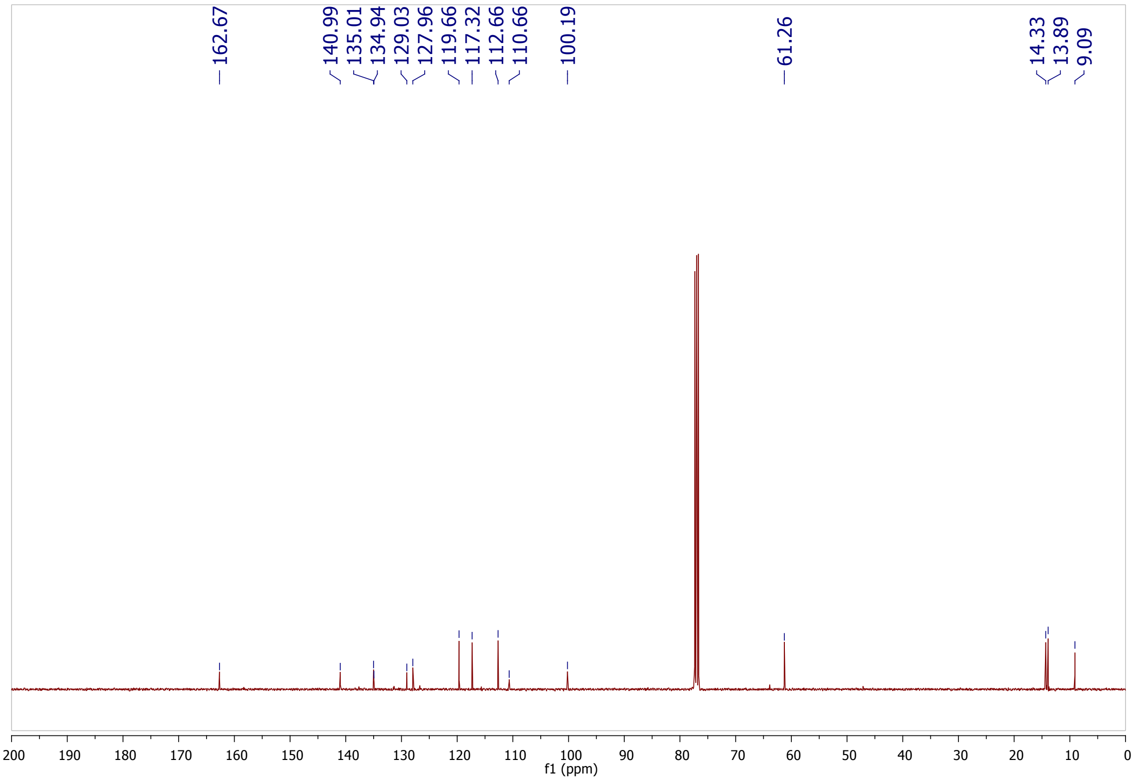

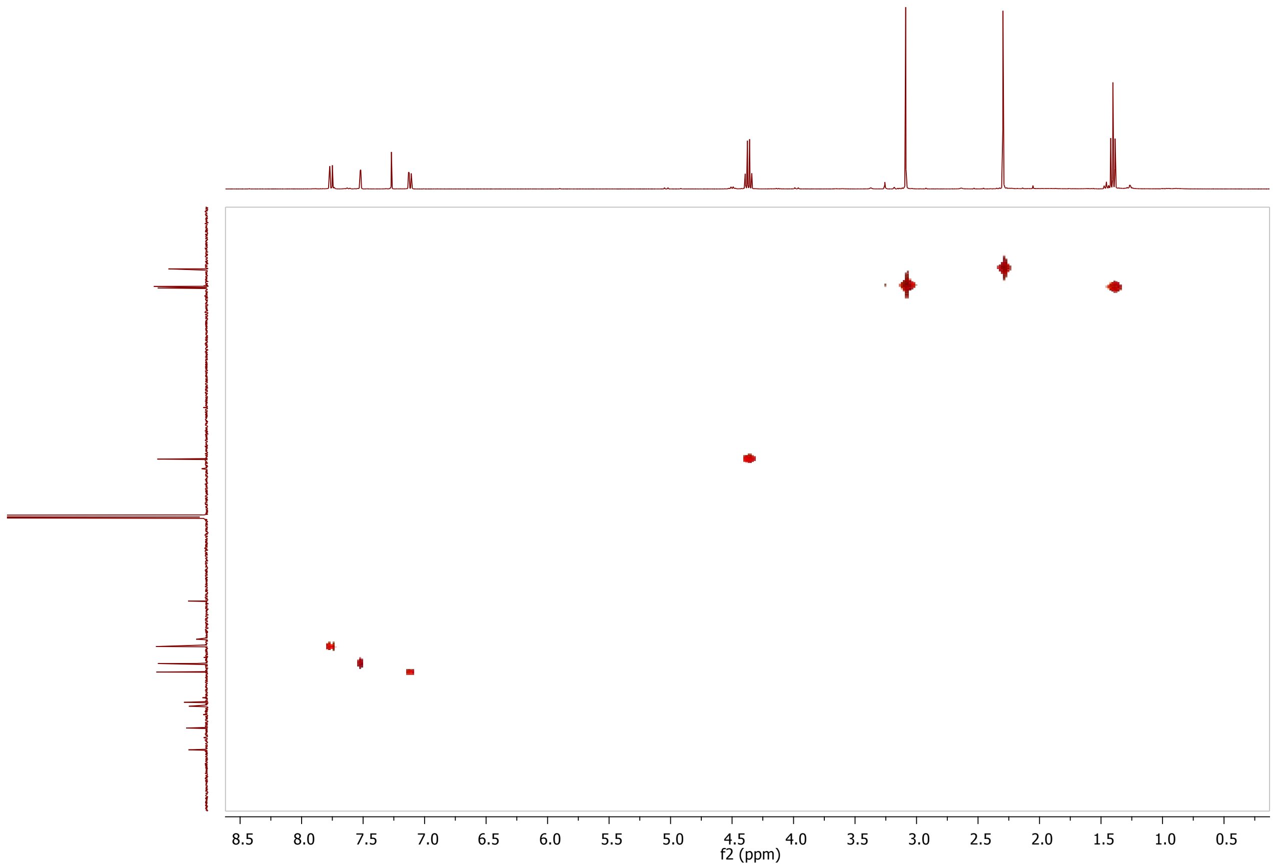
8.5
8.0
7.5
7.0
6.5
6.0
5.5
5.0
4.5
4.0
3.5
3.0
2.5
2.0
1.5
1.0
0.5
f2 (ppm)

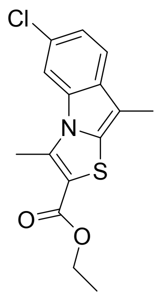

Ethyl 6-chloro-3,9-dimethylthiazolo[3,2-*a*]indole-2-carboxylate 3v.

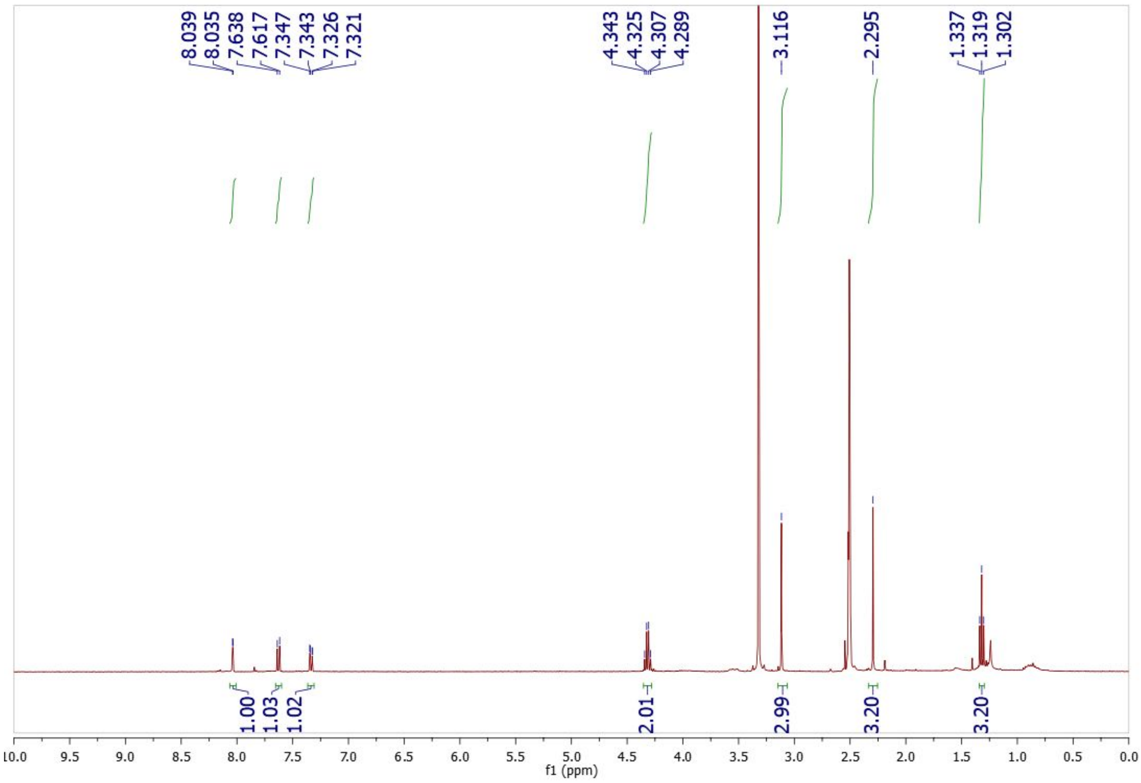

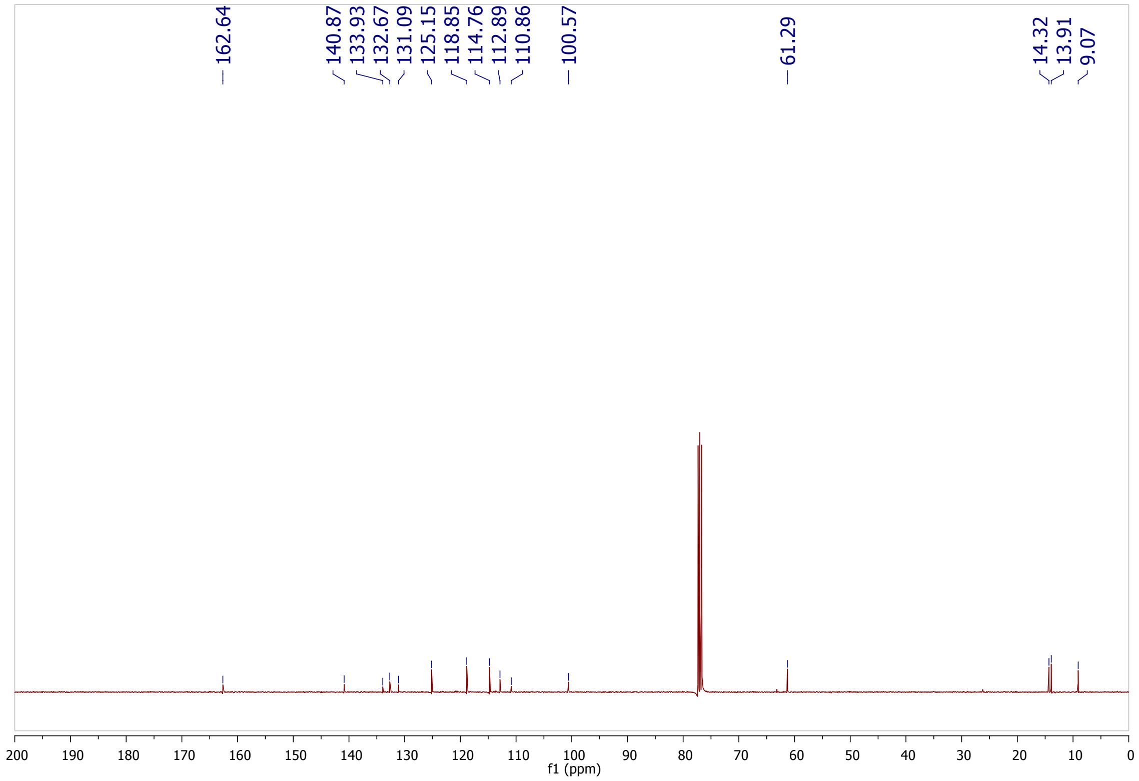

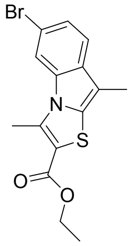

Ethyl 6-bromo-3,9-dimethylthiazolo[3,2-*a*]indole-2-carboxylate 3w.

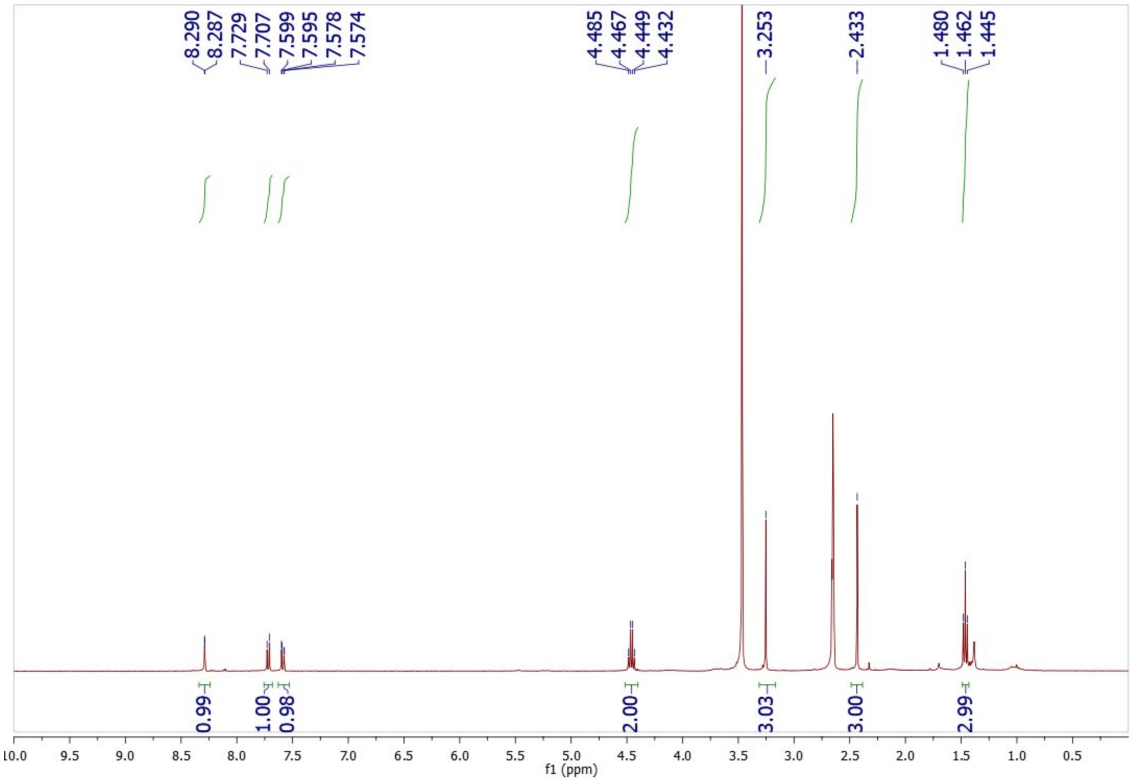

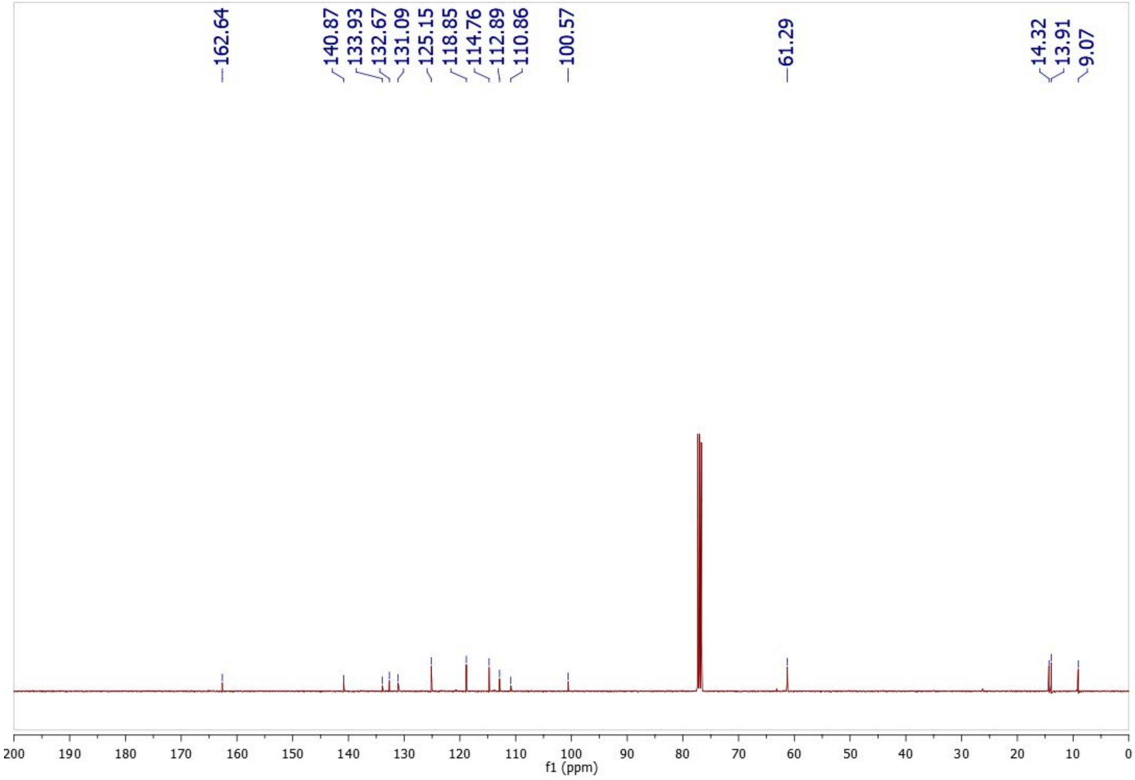

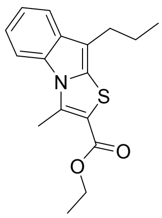

Ethyl 3-methyl-9-propylthiazolo[3,2-*a*]indole-2-carboxylate 3x.

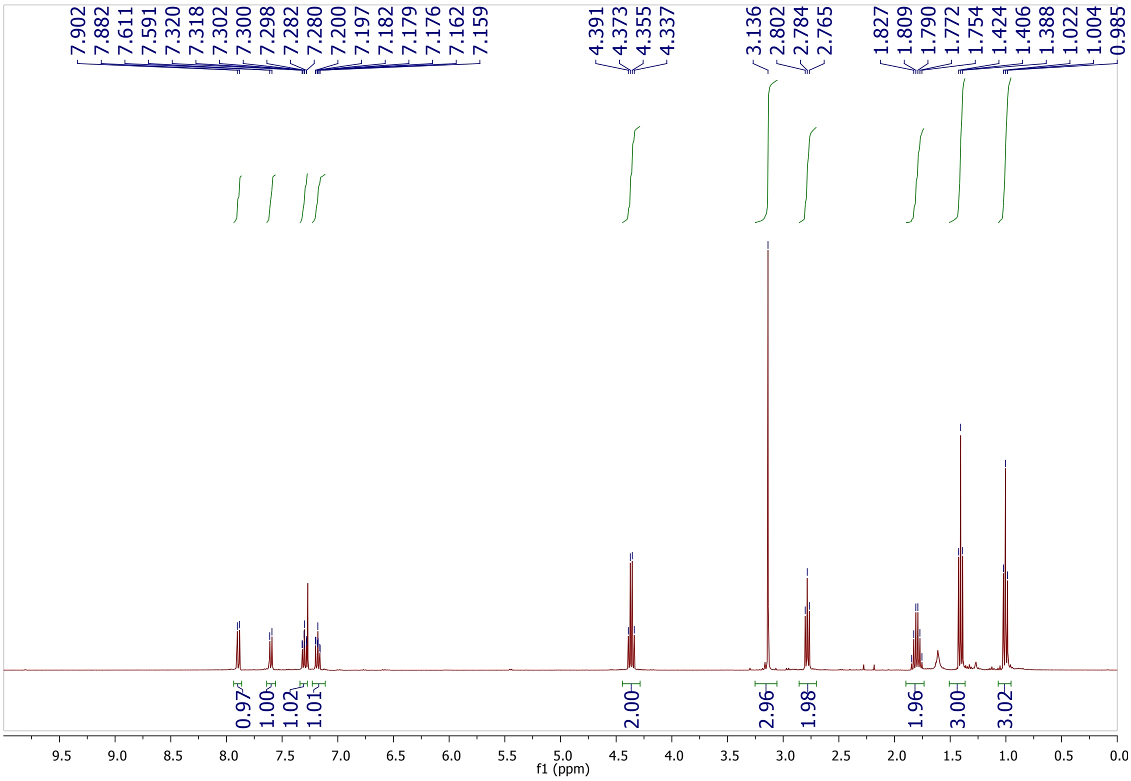

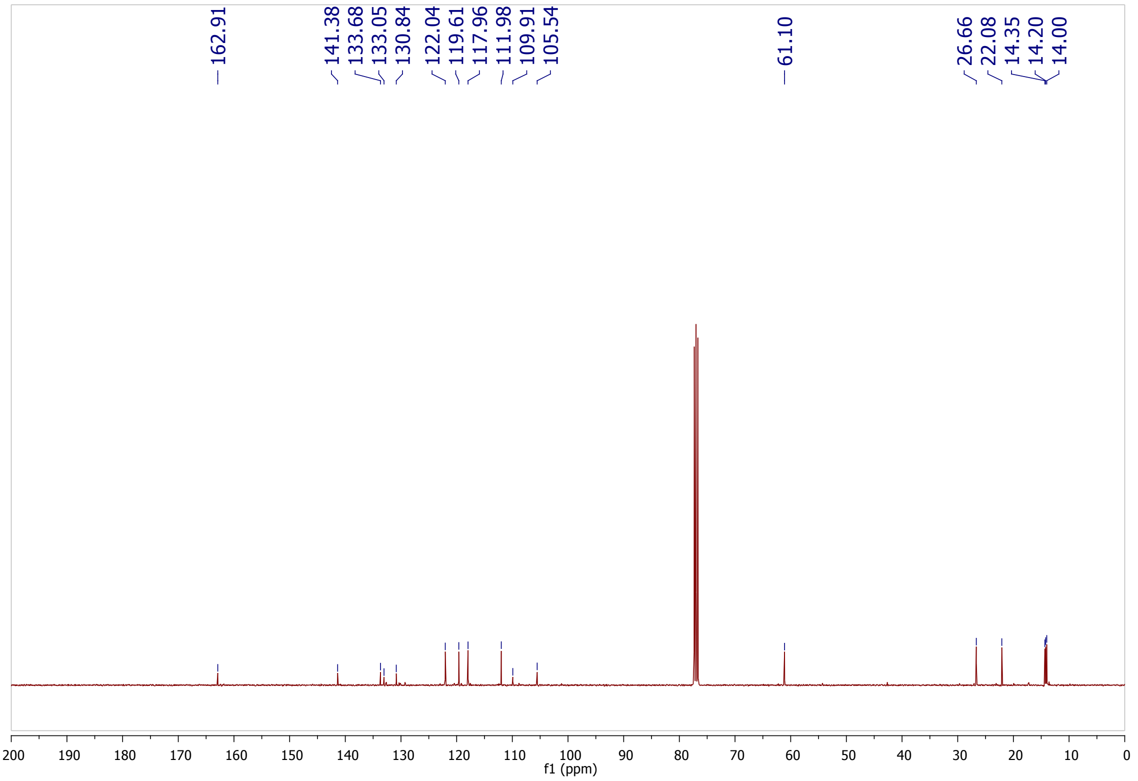

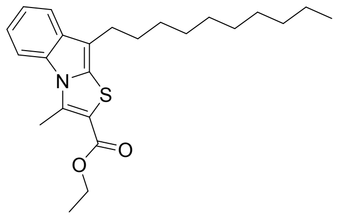

Ethyl 9-decyl-3-methylthiazolo[3,2-a]indole-2-carboxylate 3y

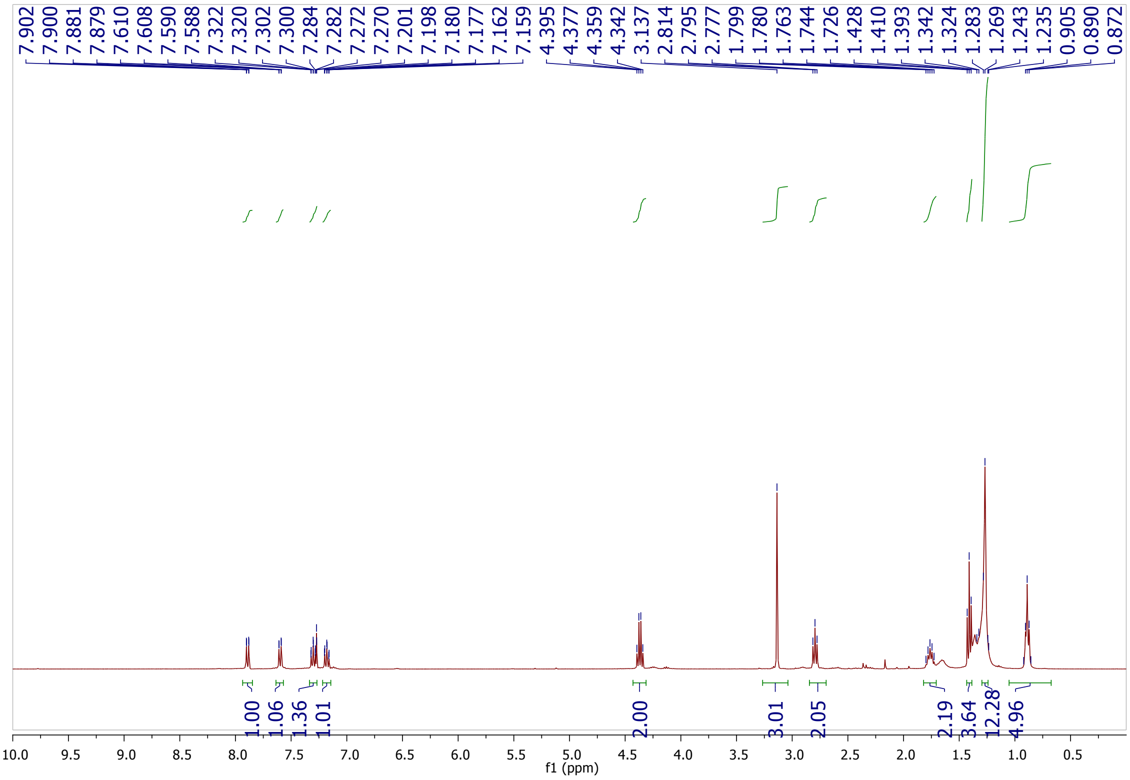

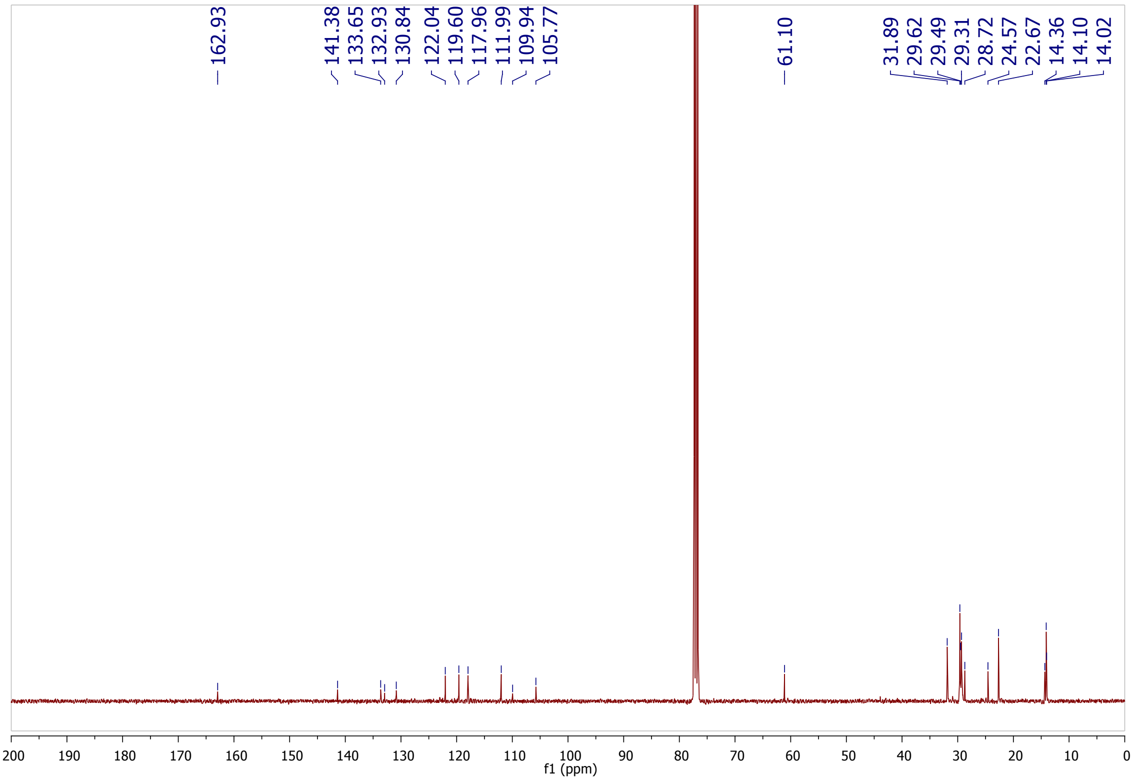

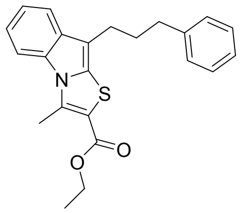

Ethyl 3-methyl-9-(3-phenylpropyl)thiazolo[3,2-a]indole-2-carboxylate 3z

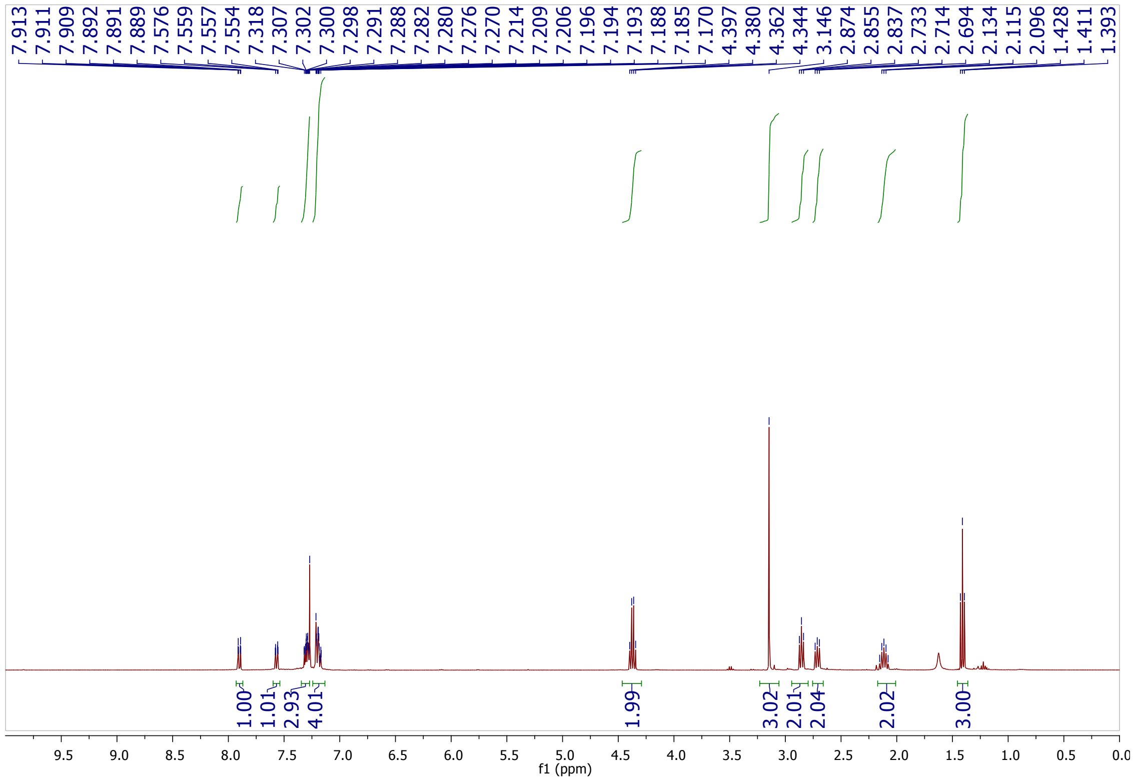

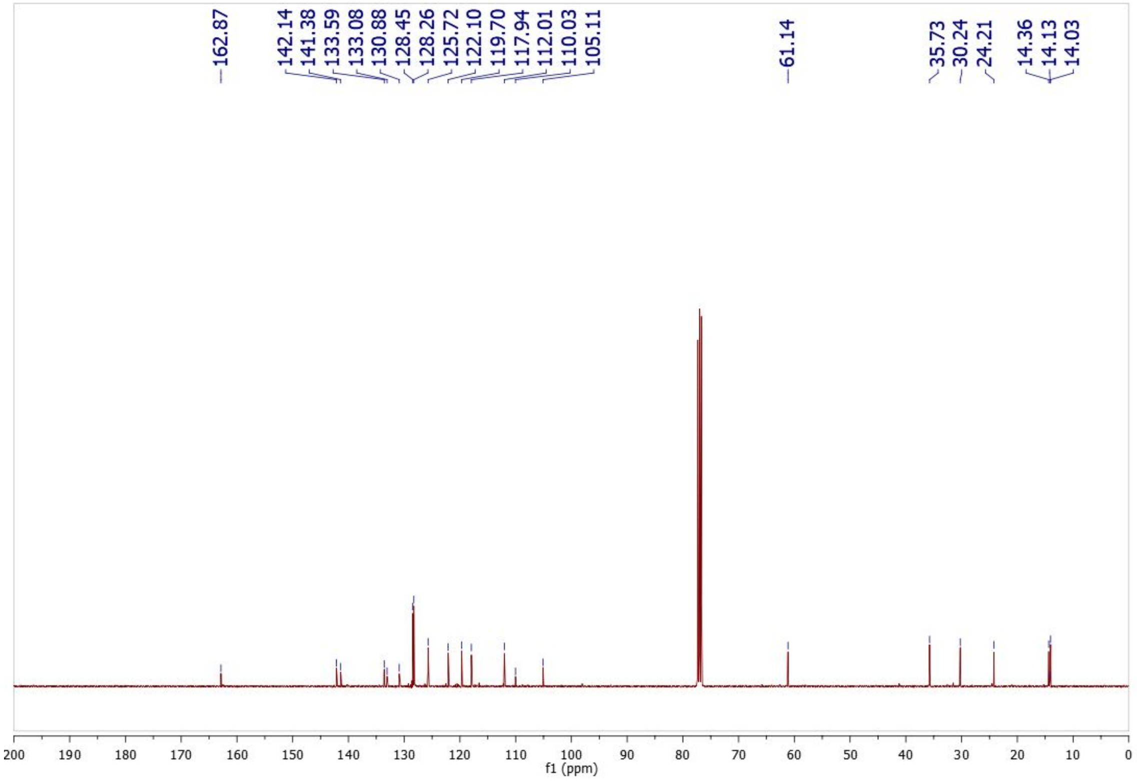

5.2 NMR spectra of compounds 6a, 8a,b.

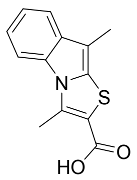

3,9-Dimethylthiazolo[3,2-*a*]indole-2-carboxylic acid 6a.

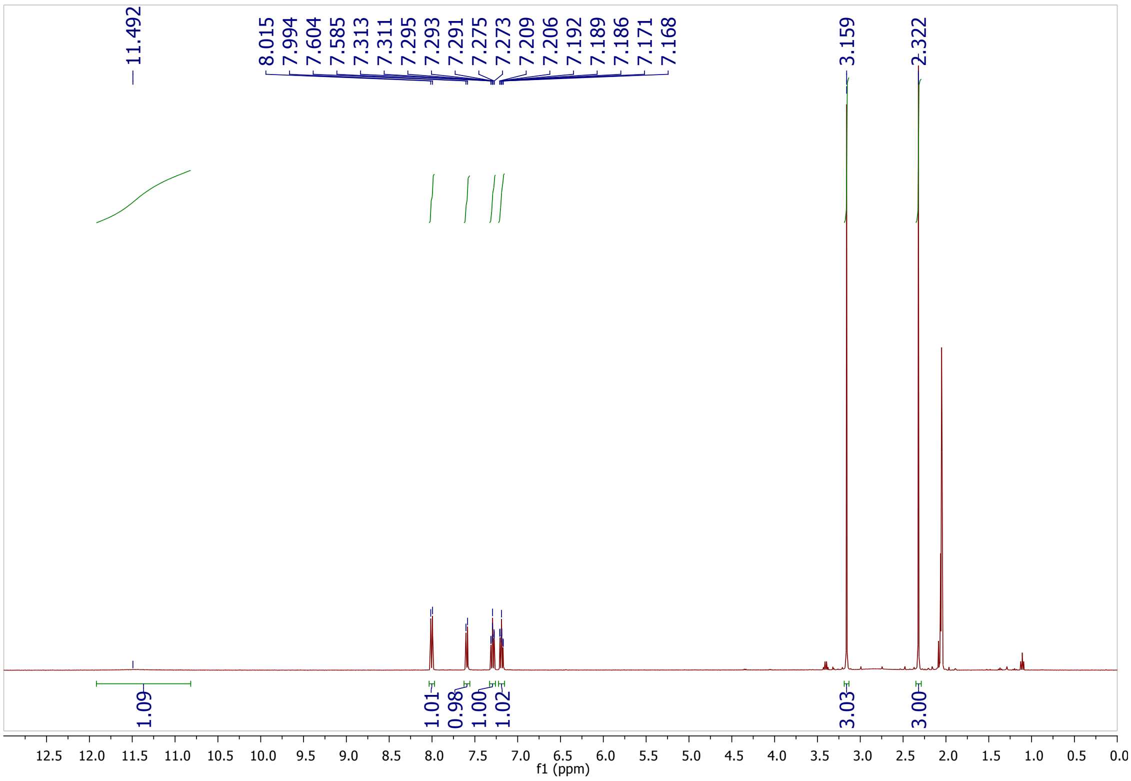

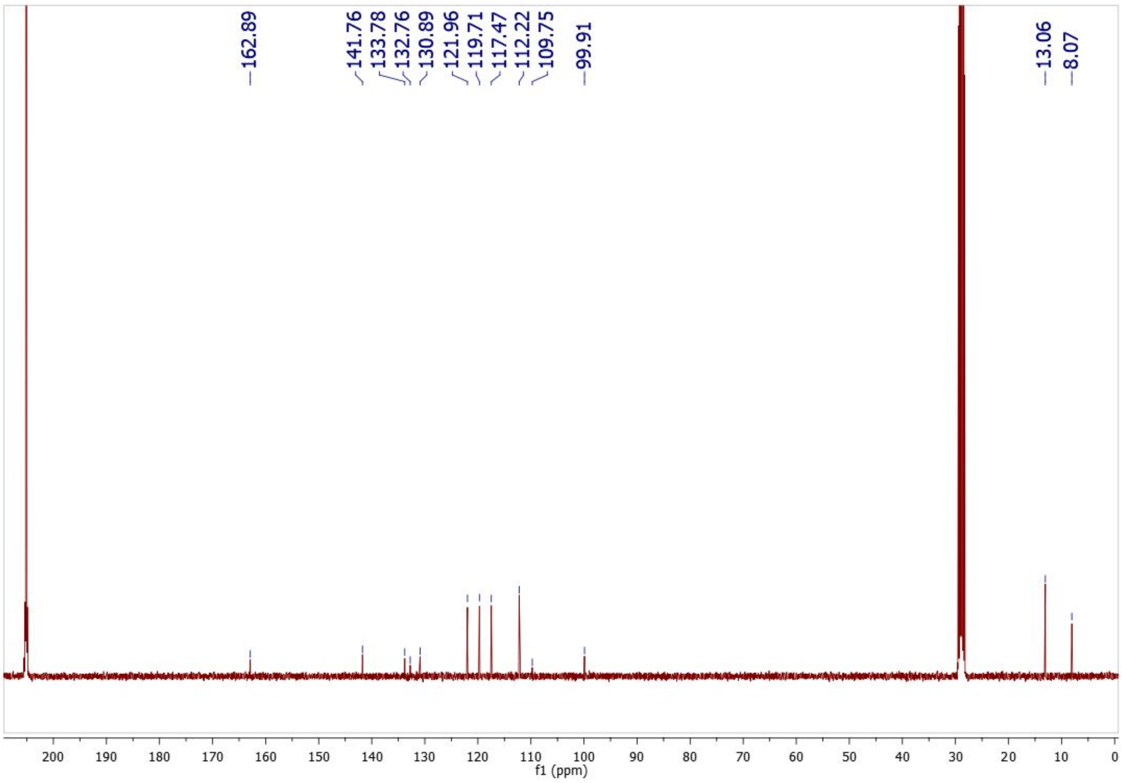

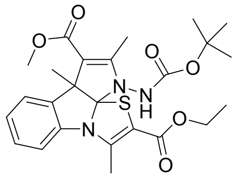

5-Ethyl 1-methyl 3-((tert-butoxycarbonyl)amino)-2,6,11b-trimethyl-3,11b-dihydropyrrolo[2,3-*b*]thiazolo[3,2-*a*]indole-1,5-dicarboxylate 8a

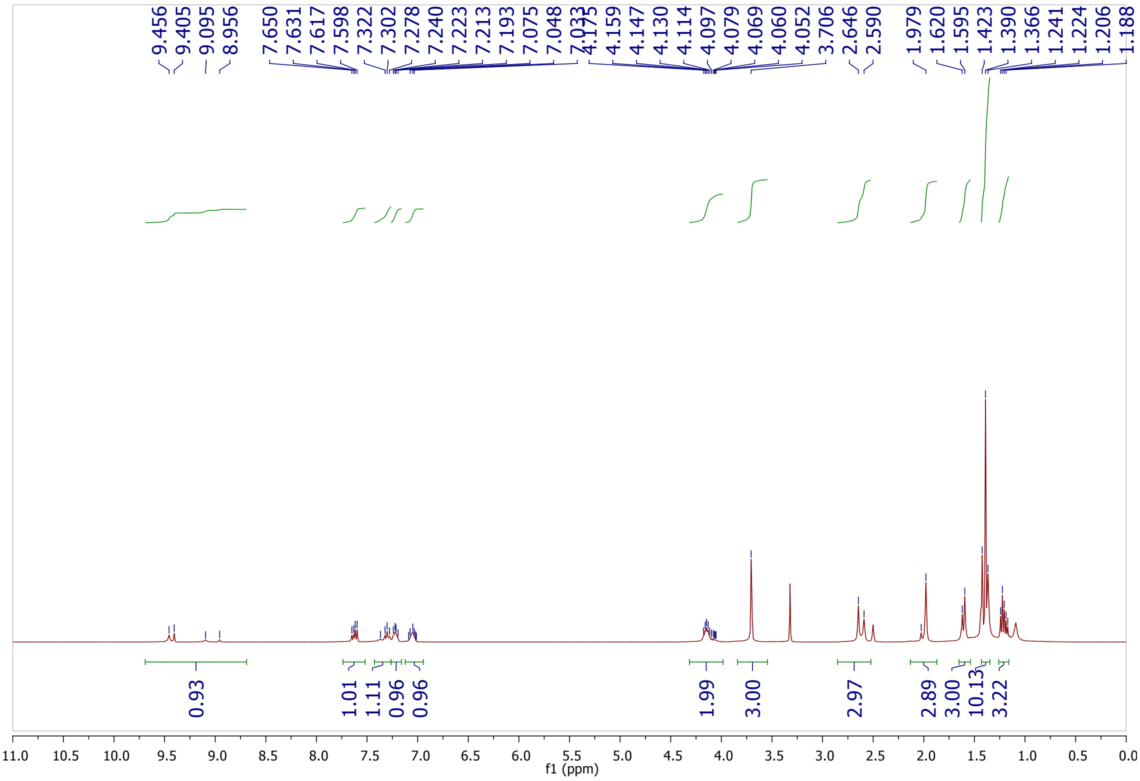

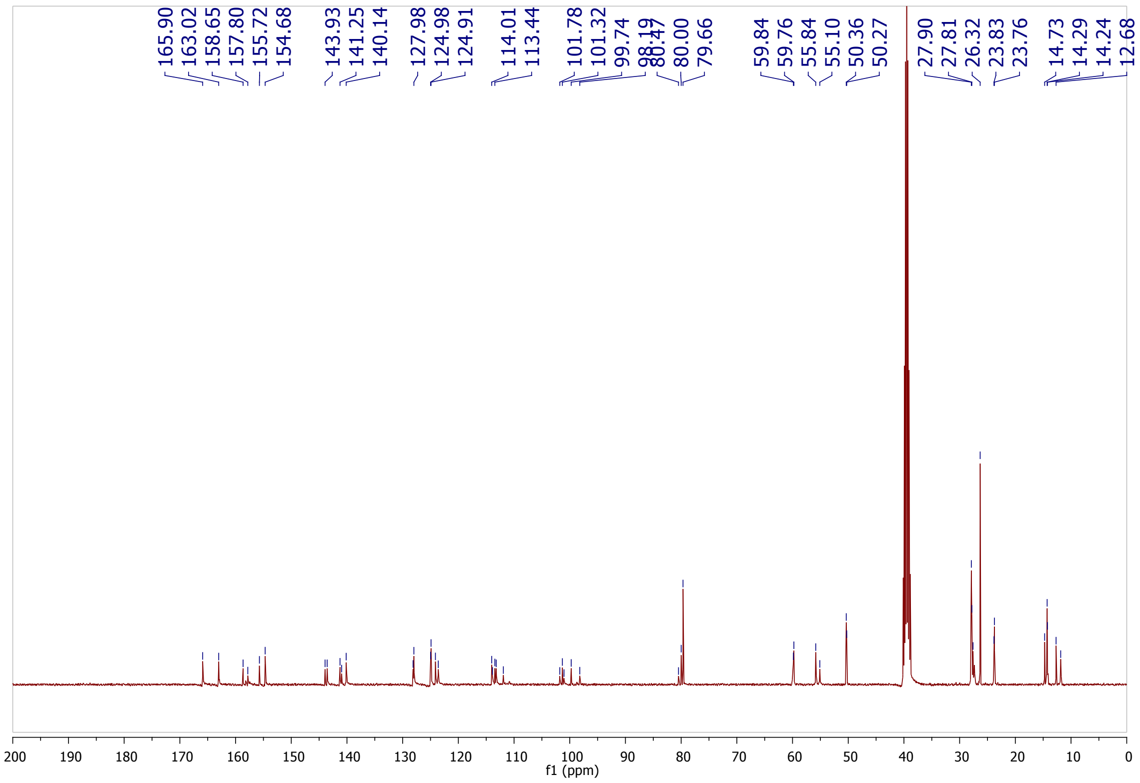

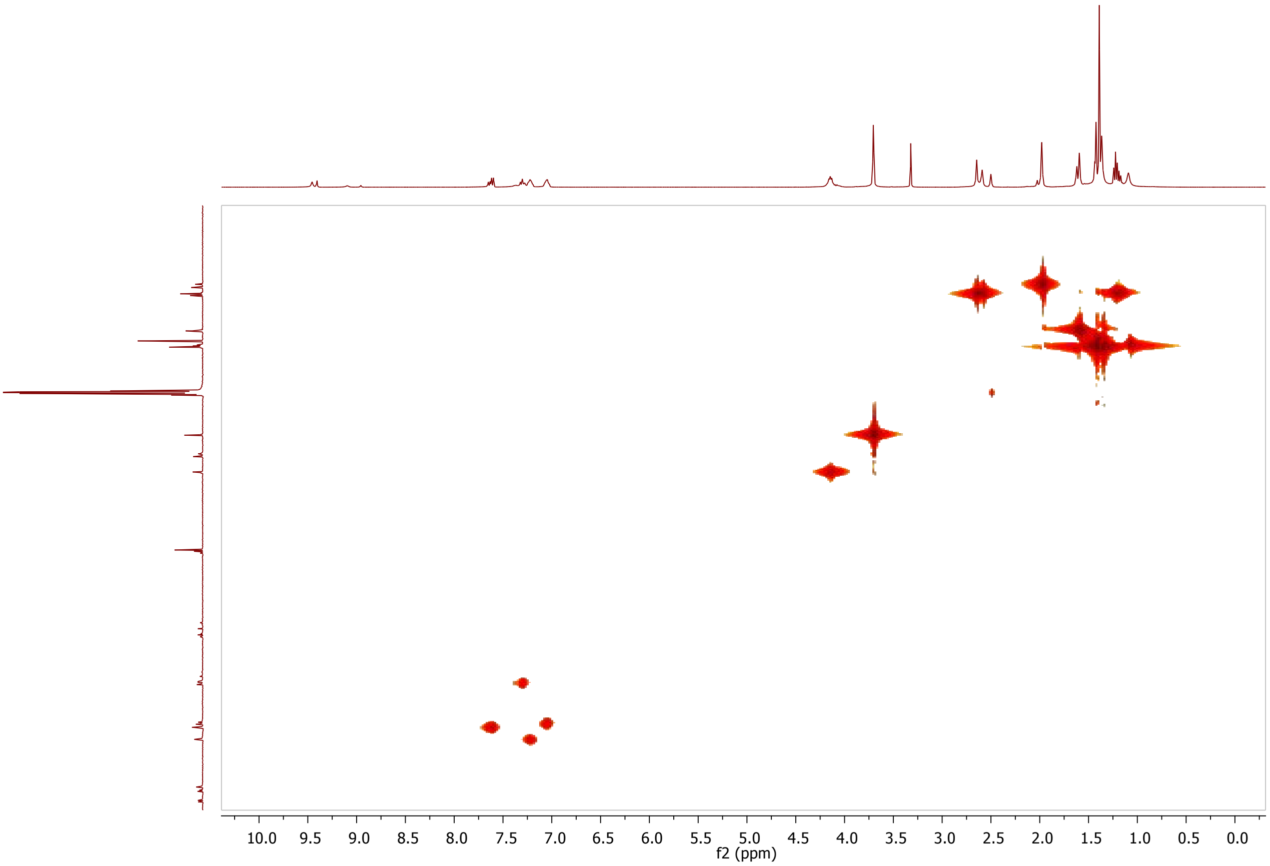
10.0
9.5
9.0
8.5
8.0
7.5
7.0
6.5
6.0
5.5
5.0
4.5
4.0
3.5
3.0
2.5
2.0
1.5
1.0
0.5
0.0
f2 (ppm)

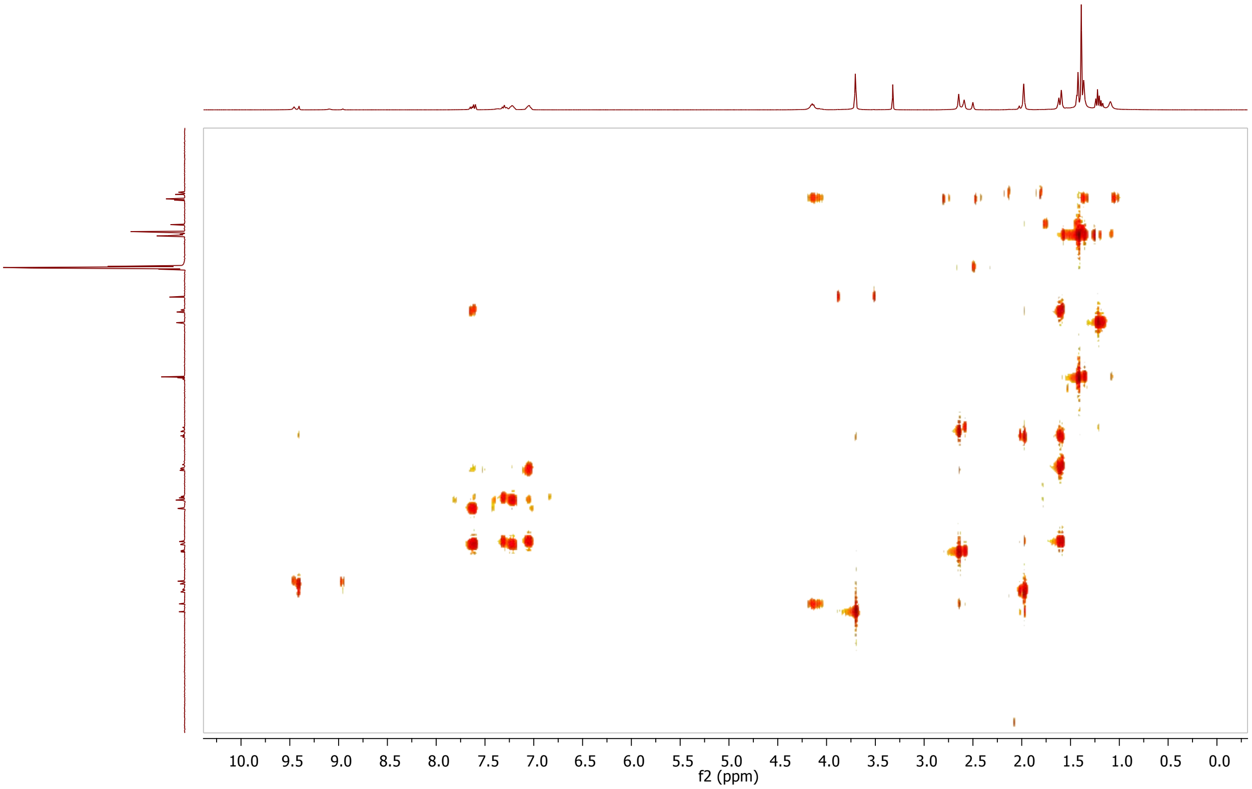
10.0
9.5
9.0
8.5
8.0
7.5
7.0
6.5
6.0
5.5
5.0
4.5
4.0
3.5
3.0
2.5
2.0
1.5
1.0
0.5
0.0
f2 (ppm)

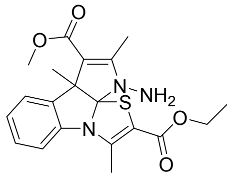

5-Ethyl 1-methyl 3-amino-2,6,11b-trimethyl-3,11b-dihydropyrrolo[2,3-*b*]thiazolo[3,2-*a*]indole-1,5-dicarboxylate 8b

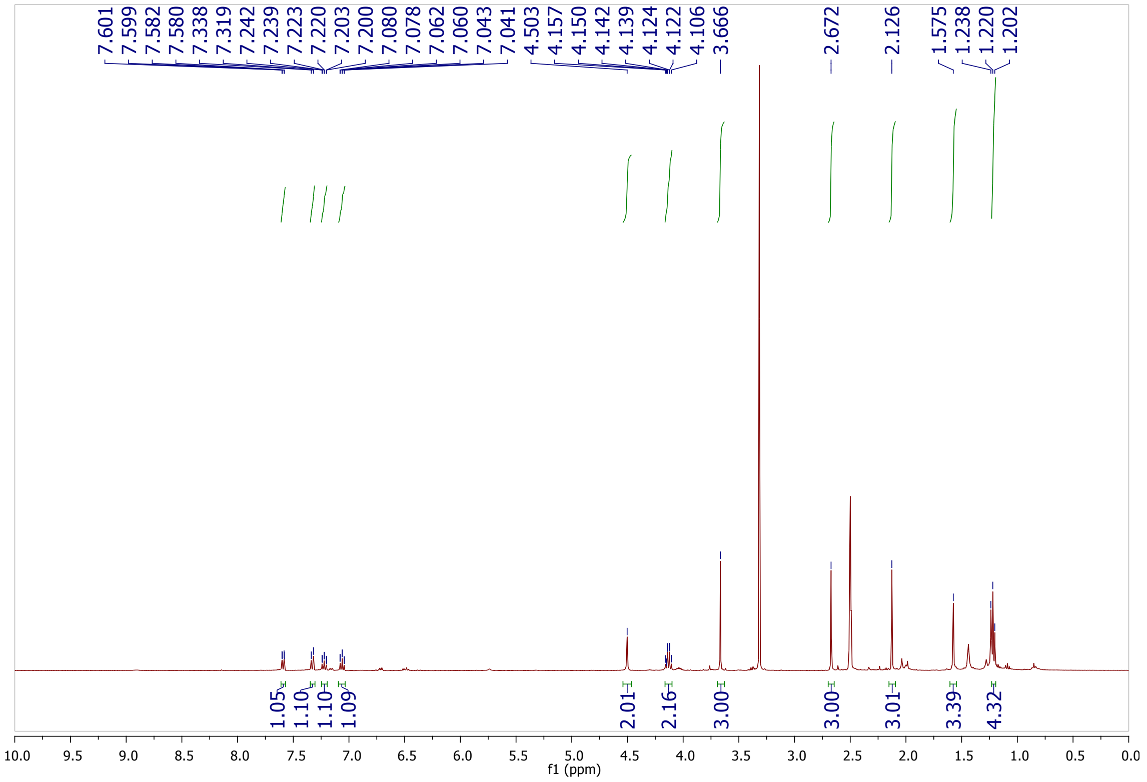

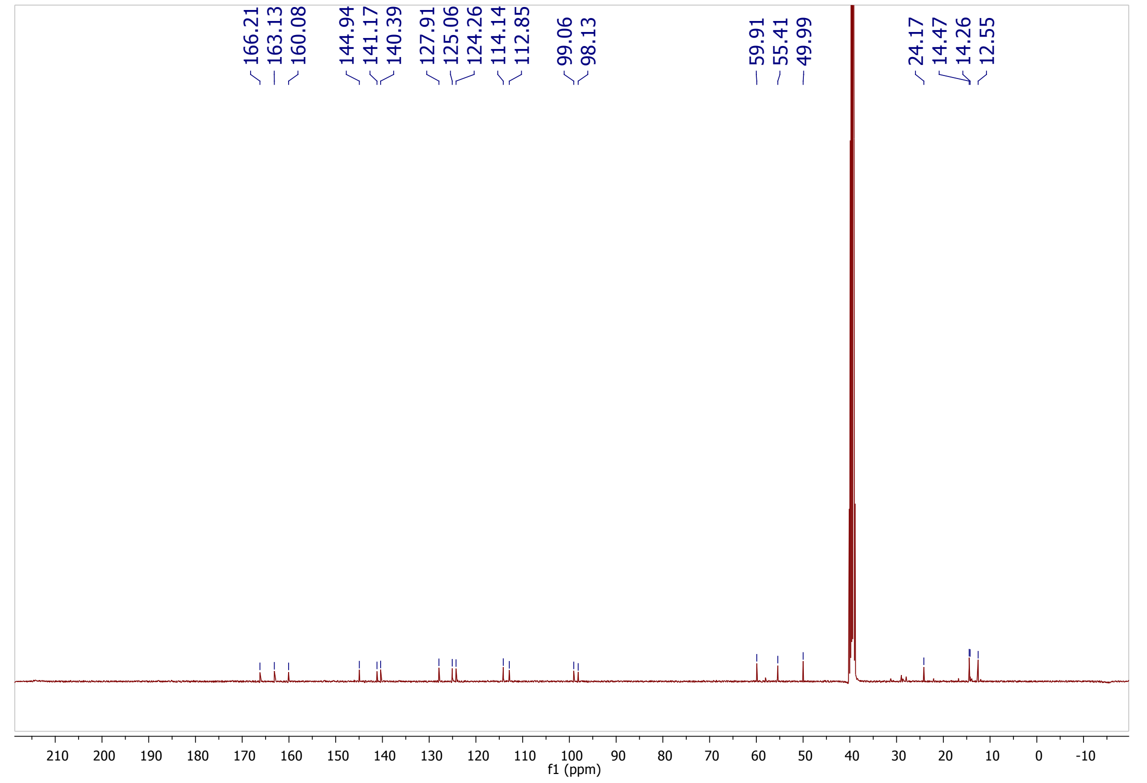

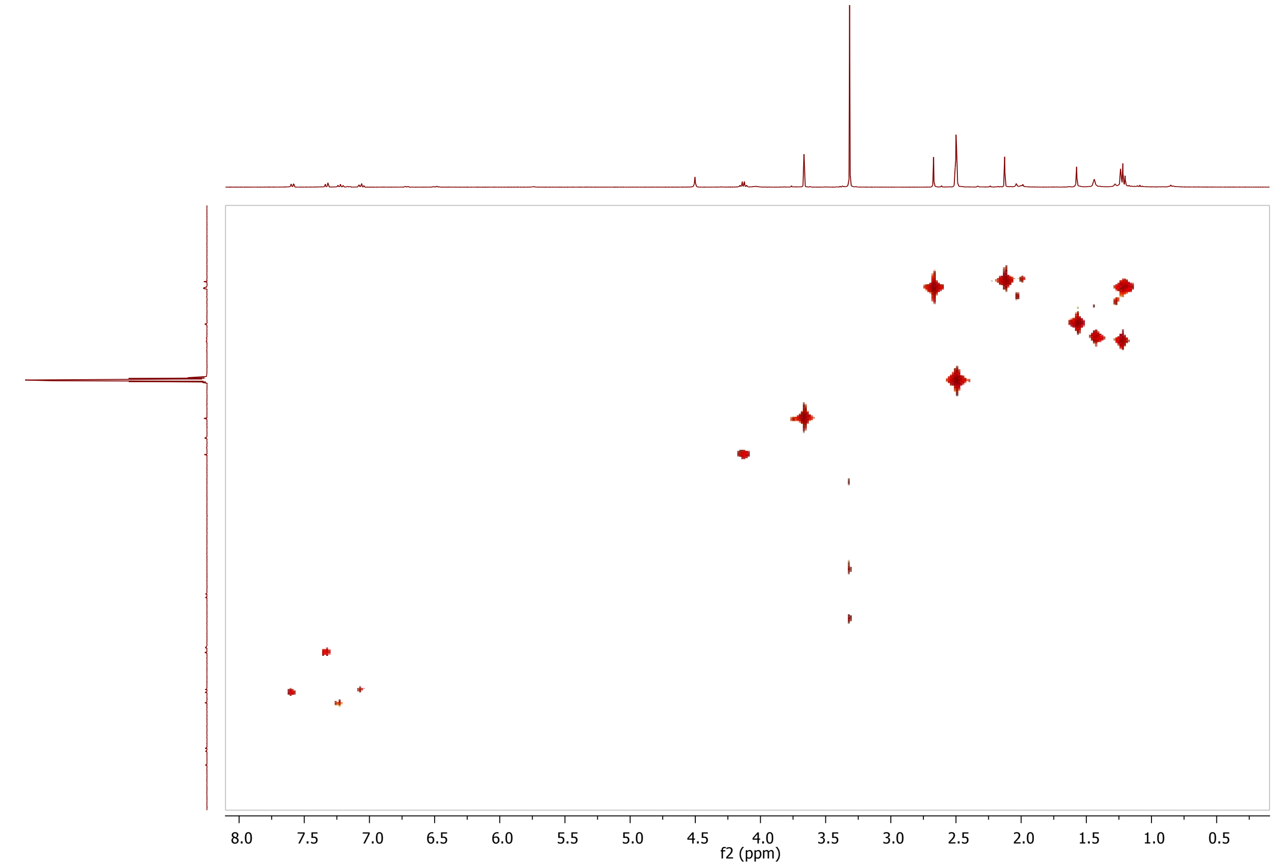
8.0 7.5 7.0 6.5 6.0 5.5 5.0 4.5 4.0 3.5 3.0 2.5 2.0 1.5 1.0 0.5
f2 (ppm)

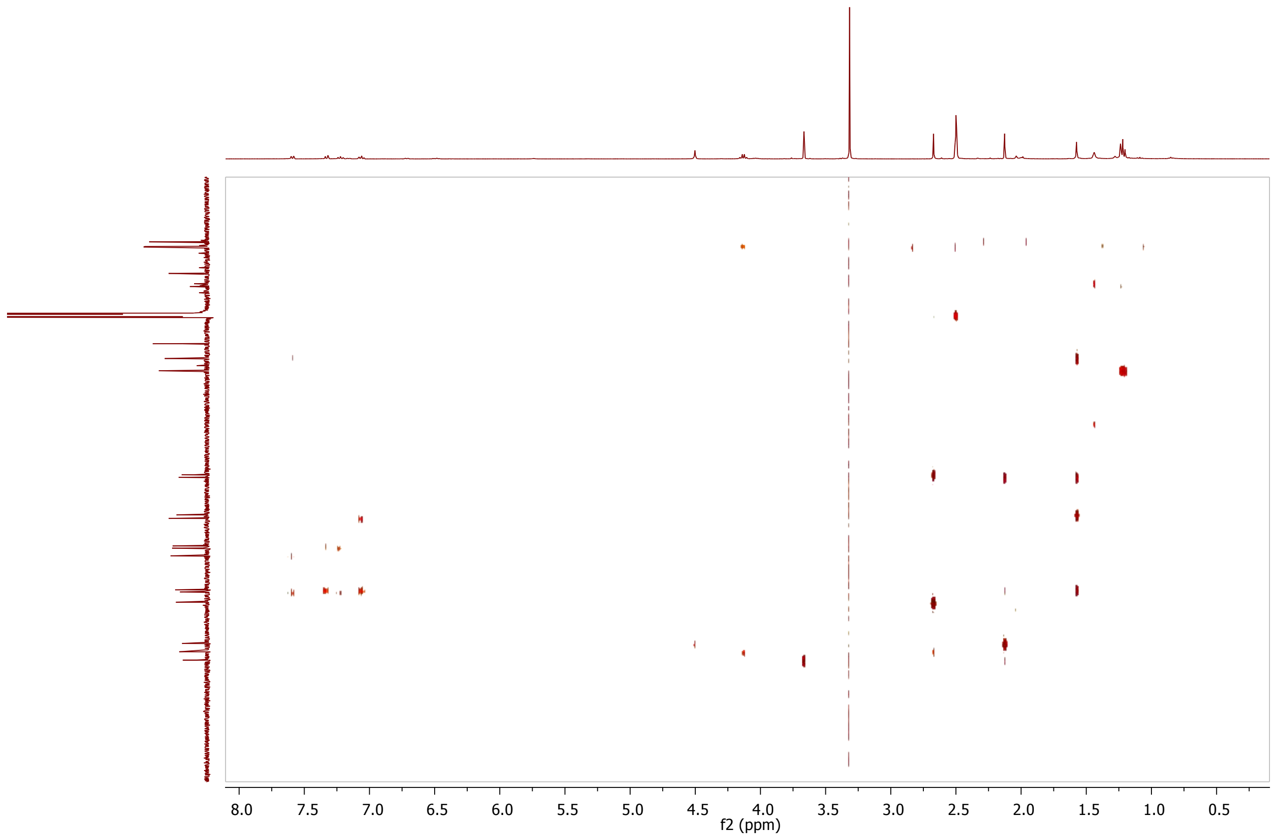
8.0 7.5 7.0 6.5 6.0 5.5 5.0 4.5 4.0 3.5 3.0 2.5 2.0 1.5 1.0 0.5
f2 (ppm)

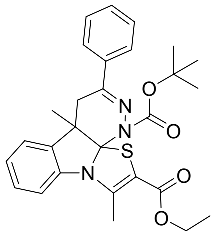

4-(*Tert*-butyl) 6-ethyl 7,12b-dimethyl-2-phenyl-1,12b-dihydro-4*H*-pyridazino[3,4-*b*]thiazolo[3,2-*a*]indole-4,6-dicarboxylate 10a.

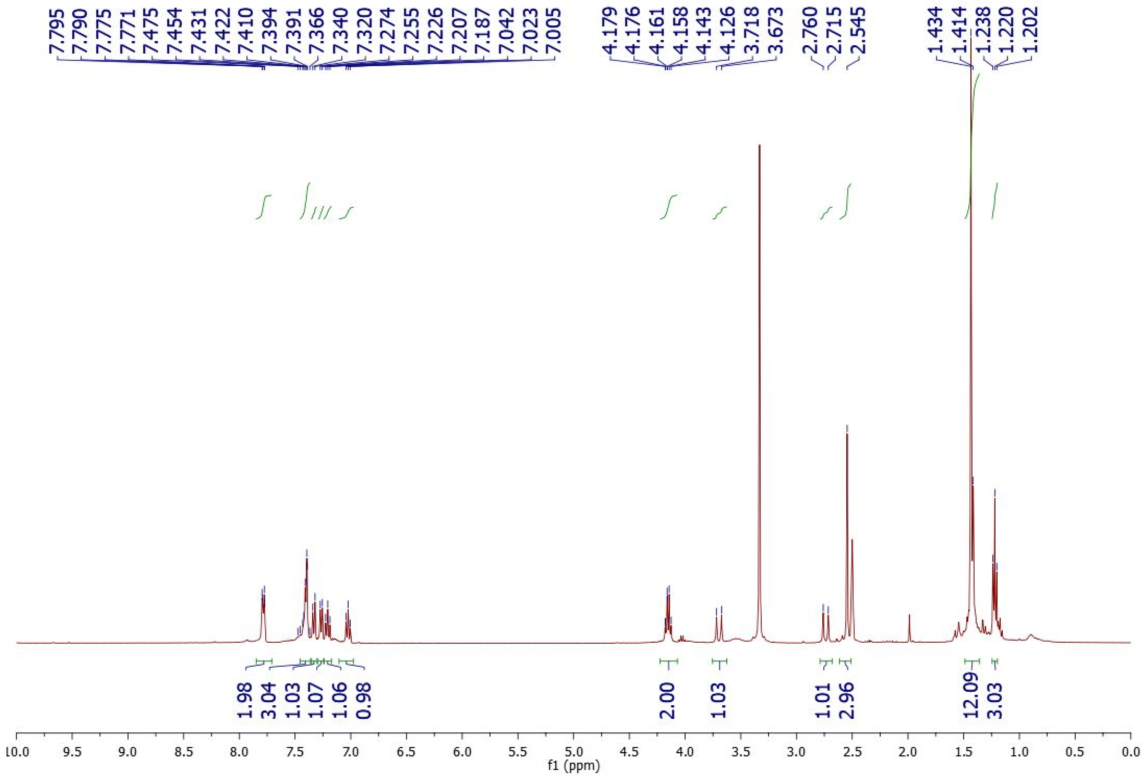

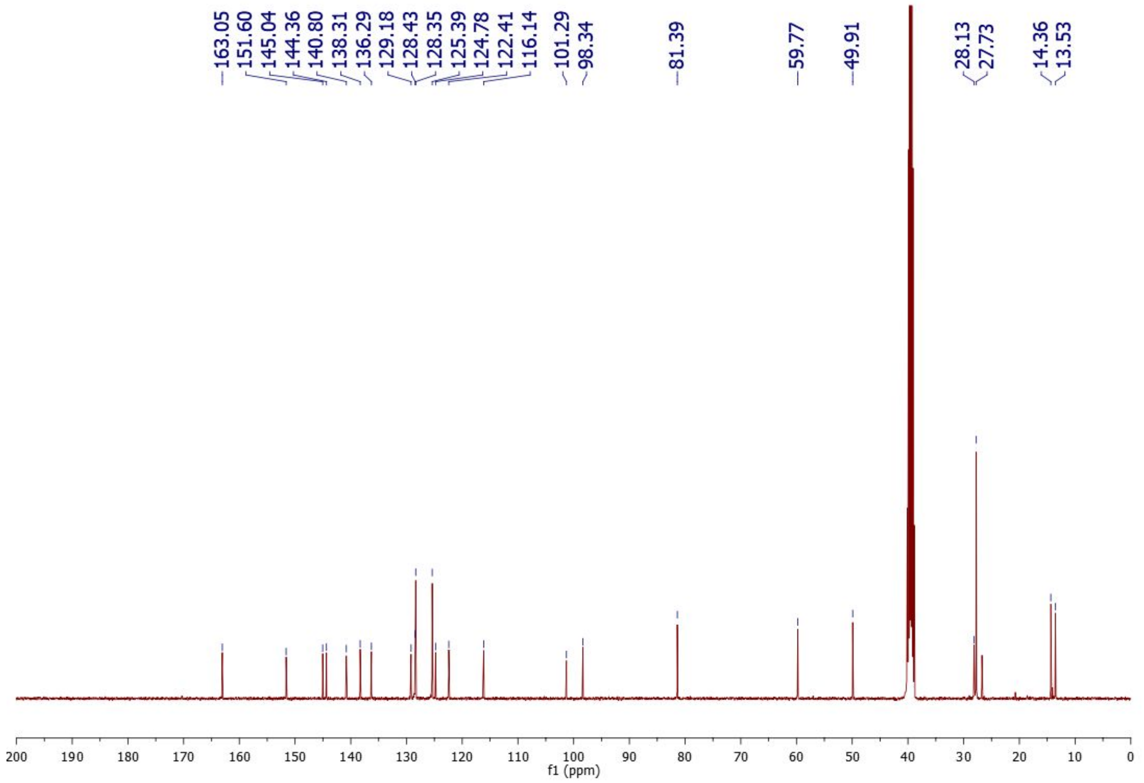

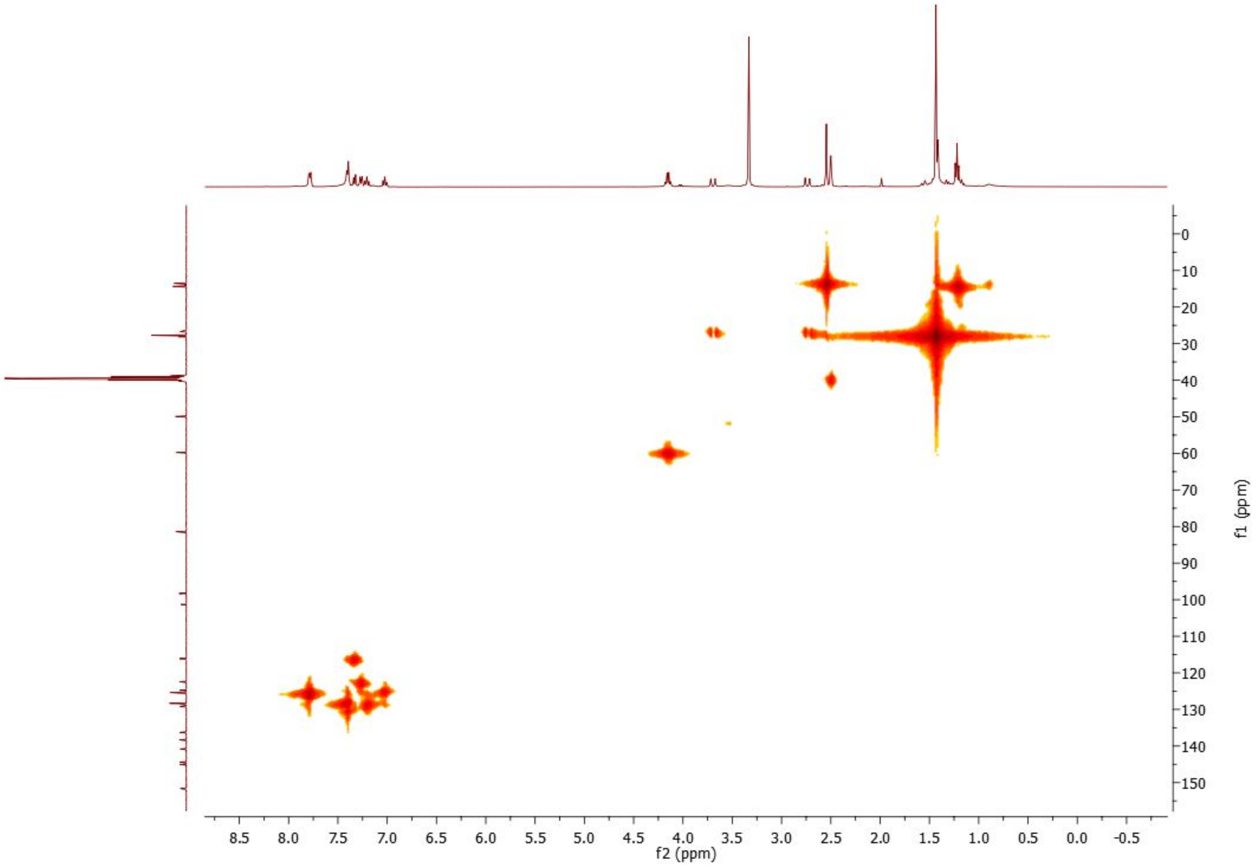
f1 (ppm)
f2 (ppm)

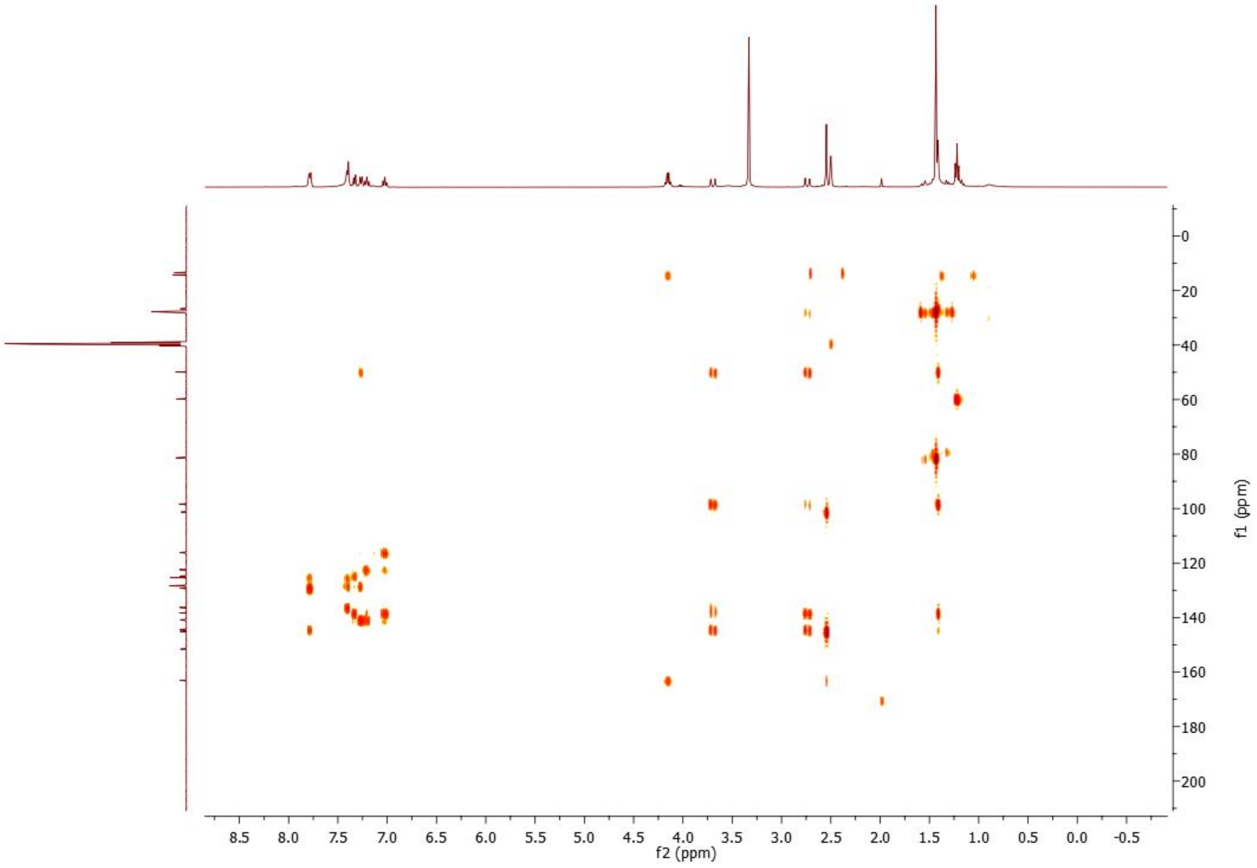
f1 (ppm)
f2 (ppm)

6 X-ray structure analysis of compounds 3a (CCDC-2295941)

Single-crystal diffraction data for compound **3a** have been collected on an Agilent SuperNova dual source diffractometer with an Atlas detector at 150 K using a mirror monochromator and MoKα radiation with λ = 0,71073 Å. The diffraction data were processed using CRYSALIS PRO software.[5] Structure was solved by direct methods, using SIR2014.[6] Full-matrix least-squares refinements on F^2 were done with anisotropic displacement parameters for all non-hydrogen atoms. H atoms from methylene and methyl groups were located using difference Fourier map. Their coordinates were refined together with their isotropic displacement parameter. Aromatic H atoms were placed at calculated positions and treated as riding model. SHELXL-2018/3 software[7] was used for structure refinement and interpretation. Drawing of the structure (Figure 4) was produced using ORTEP-III.[8] Structure is orthorhombic, with *Pbcm* space group and a = 11.4140(4), b = 17.0326(5), c = 6.8641(2) Å. In the unit cell there are 4 molecules with formula $C_{15}H_{15}NO_2S$. All non-hydrogen atoms and 7 hydrogen atoms of each molecule lie on crystallographic mirror plane. Structural and other crystallographic details on data collection and refinement have been deposited with the Cambridge Crystallographic Data Centre as supplementary publication numbers CCDC Deposition Number 2295941. These data can be obtained free of charge via www.ccdc.cam.ac.uk/conts/retrieving.html (or from the CCDC, 12 Union Road, Cambridge CB2 1EZ, UK; fax: ţ44 1223 336033; e-mail: deposit@ccdc.cam.ac.uk).

6.1 Figure S1. ORTEP diagram of compound 3a

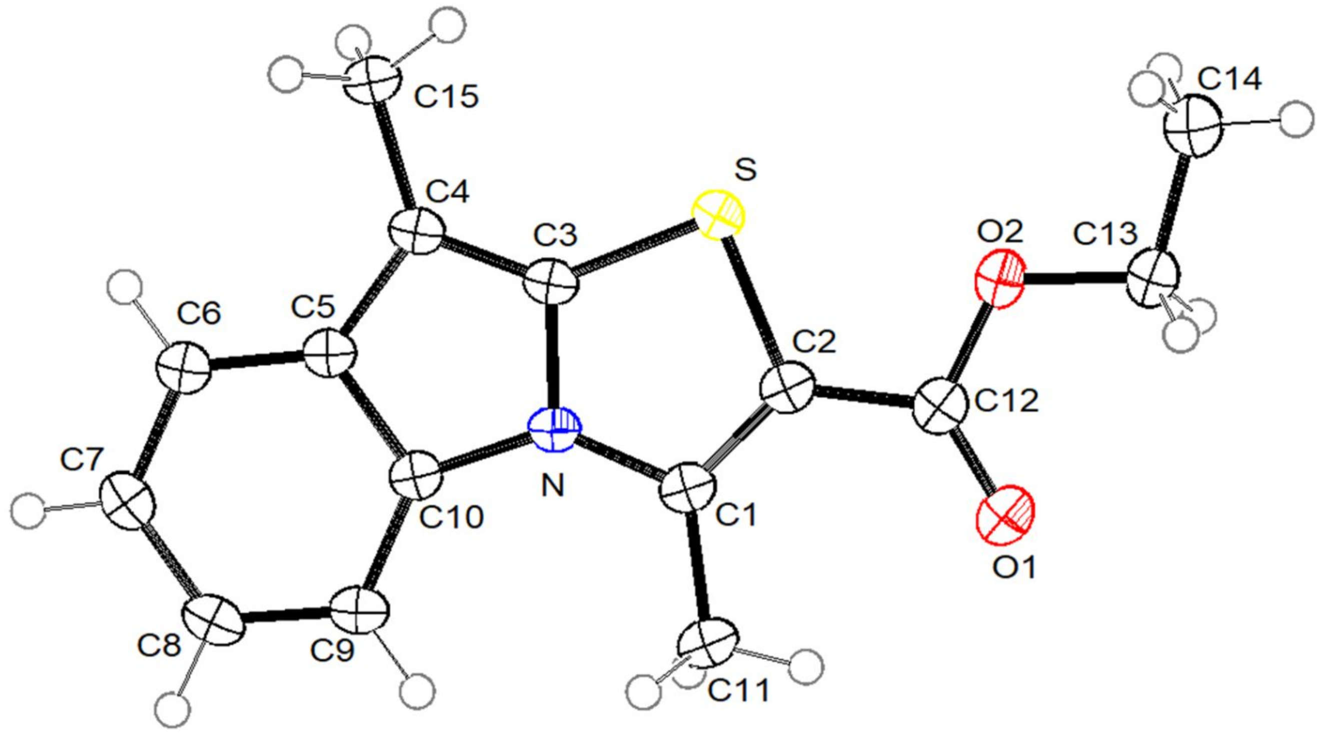

Figure S1. ORTEP diagram of compound **3a** with ellipsoids at 50 % probability level (CCDC-2295941).

6.2 Table S5. Crystal data and structure refinement for 3a.

Empirical formula	$C_{15}H_{15}NO_2S$
Formula relative weight	273.34
Temperature/K	150(1)
Crystal system	orthorhombic
Space group	*Pbcm,* no.57
a/Å	11.4140(4)
b/Å	17.0326(5)
c/Å	6.8641(2)
$\alpha = \beta = \gamma/^\circ$	90
Volume/$Å^3$	1334.45(7)
Z	4
ρ_{calc}/gcm^{-3}	1.361
μ/mm^{-1}	0.239
F(000)	576
Crystal size/mm^3	0.3 × 0.3 × 0.1
Colour, shape	yellow plate
Radiation Mo Kα, λ /Å	0.71073
Θ range - data collection/°	2.98 to 30.40
Index ranges	$-14 \leq h \leq 14$, $-23 \leq k \leq 23$, $-9 \leq l \leq 9$
Reflections collected	25732
Independent reflections	2018 [R_{int} = 0.0451]
Data/parameters	2018/140
Goodness-of-fit on F^2	1.133
Final R indexes	[I>=2σ (I)] R_1 = 0.0395, wR_2 = 0.1027
Final R indexes [all data]	R_1 = 0.0476, wR_2 = 0.1104
Largest diff. peak/hole /e $Å^{-3}$	0.48/-0.31

7 Green metrics: First pass Metric Toolkit.

Supplementary Information: Appendix 2

Summary of First Pass Metrics Toolkit

Yield, AE, RME, MI/PMI and OE

Reactant (Limiting Reactant First)	Mass (g)	MW	Mol	Catalyst	Mass (g)	Reagent	Mass (g)	Reaction solvent	Volume (cm^3)	Density (g ml^{-1})	Mass (g)	Work up chemical	Mass (g)	Workup solvent	Volume (cm3)	Density (g ml^{-1})	Mass (g)
3-alkyl-indoline-2-thiones **1a**	0,11	163,20	0,00					water	1,00	1,00	1,00	NaCl	1,80	ethyl acetate	15,00	0,92	13,80
ethyl 2-chloro acetoacetate **2a**	0,12	164,60	0,00								0,00						0,00
			#DIV/0!								0,00						0,00
			#DIV/0!								0,00						0,00
			#DIV/0!								0,00						0,00
			#DIV/0!								0,00						0,00
			#DIV/0!								0,00						0,00
Total	0,23	327,80			0,00		0,00				1,00		1,80				13,80

		Flag	
Yield	97,0	●	97,0
Conversion	100,0	●	100,0
Selectivity	97,0	●	97,0
AE	83,4		
RME	81,0	OE	97,1
PMI total	90,7		
PMI Reaction	6,6		
PMI reactants, reagents, catlyst	1,2		
PMI reaction solvents	5,4		
PMI Workup	84,1		
PMI Workup chemical	9,7		
PMI workup solvents	74,4		

	Mass	MW	Mol
Product	0,19	273,35	0,00
	mass		
Unreacted limiting reactant	0,00		

$$RME = \frac{mass\ of\ isolated\ product}{total\ mass\ of\ reactants} \times 100$$

$$AE = \frac{molecular\ weight\ of\ product}{total\ molecualr\ weight\ of\ reactants} \times 100$$

$$mass\ intensity = \frac{total\ mass\ in\ a\ process\ or\ process\ step}{mass\ of\ product}$$

$$OE = \frac{RME}{AE} \times 100$$

Experimental:
To a solution of substituted 3-methyl-indoline-2-thiones **1a** (0.7 mmol, 1.0 equiv.) in water (1.0 mL), ethyl 2-chloro acetoacetate **2a** (0.7 mmol, 1.0 equiv.) was added and the reaction mixtures was heated at 60°C until the disappearance of the reagents (monitored by TLC, elution mixture cyclohexane: ethyl acetate, 95 : 5; 5.0 -14.0 h). Then the reaction mixture was cooled at room temperature, the crude was saturated with sodium chloride, and then extracted with ethyl-acetate (3 x 5.0 mL). The solvent was then evaporated under reduced pressure furnishing directly the desired thiazol[3,2-*a*]indole **3a**.

Solvents (First Pass)

		List solvents below
**Preferred solvents**	**water,** EtOH, nBuOH, AcOipr, AcOnBu, PhOMe, MeOH, tBuOH, BnOH, ethylene glycol, acetone, MEK, MIBK, **AcOEt,** sulfolane	X
**Problematic solvents:** (acceptable only if substitution does not offer advantages)	DMSO, cyclohexanone, DMPU, AcOH, Ac2O, Acetonitrile, AcOMe, THF, heptane, Me-cyclohexane, toluene, xylene, MTBE, **cyclohexane**, chlorobenzene, formic acid, pyridine, Me-THF	
**Hazardous solvents**: These solvents have significant health and/or safety concerns.	dioxane, pentane, TEA, diisopropyl ether, DME, DCM, DMF, DMA, NMP, methoxyethanol, hexane	
**Highly hazardous solvents:** The solvents which are agreed not to be used, even in screening	Et_2O, Benzene, CCl_4, chloroform, DCE, nitromethane, CS_2, HMPA	

Instructions for use: Enter your data into the tables above to automatically calculate yield, AE, RME, MI/PMI and OE.
Use the blank boxes in the tables to enter experimental data and note the flags for each Key Parameter.
Printing tips: This spreadsheet is designed to be printed with 'landscape', 'narrow margin' and 'fit all columns on one page' settings

Catalyst/enzyme (First Pass)

		Tick
Catalyst or enzyme used, or reaction takes place without any catalyst/reagents.	Green Flag	X
Use of stoichiometric quantities of reagents	Amber Flag	
Use of reagents in excess	Red Flag	

		Tick
Facile recovery of catalyst/enzyme	Green Flag	
catalyst/enzyme not recovered	Amber Flag	

Critical elements

Supply remaining	Flag colour	Note element
5-50 years	Red Flag	
50-500 years	Amber Flag	
+500 years	Green Flag	

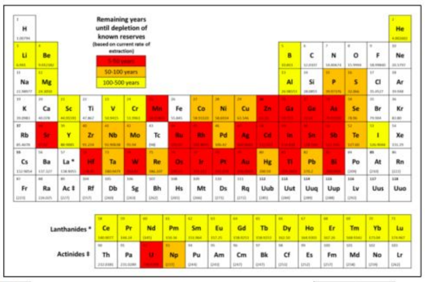

Energy (First Pass)

		Tick
Reaction run between 0 to 70°C	Green Flag	X
Reaction run between -20 to 0 or 70 to 140°C	Amber Flag	
Reaction run below -20 or above 140°C	Red Flag	

		Tick
Reaction run at reflux	Red Flag	
Reaction run 5°C or more below the solvent boiling point	Green Flag	X

Batch/flow

		Tick
Flow	Green Flag	
Batch	Amber Flag	X

Work Up

		List
quenching<br>filtration<br>centrifugation<br>crystallisation<br>Low temperature<br>distillation/evaporation/ sublimation (<	Green Flag	
solvent exchange, quenching into aqueous solvent	Amber Flag	X
chromatography/ion exchange<br>high temperature<br>multiple recrystallisation	Red Flag	

Health & safety

	Red Flag	Amber Flag	Green Flag	List substances and H-codes	List substances and H-codes	List substances and H-codes
						ethyl 2-chloro acetoacetate 2a H226, H302, H314
Highly explosive	H200, H201, H202, H203	H205, H220, H224	If no red or amber flagged H codes present then green flag			
Explosive thermal runaway	H230, H240, H250	H241				
Toxic	H300, H310, H330	H301, H311, H331,				
Long Term toxicity	H340, H350, H360, H370, H372	H341, H351, H361, H371, H373				
Environmental implications	H400, H410, H411, H420	H401, H412				

Use of chemicals of environmental concern		List substances of very high concern
Chemical identified as Substances of Very High Concern by ChemSec which are utilised	Red Flag	

8 References

1) The 3-methylindoline-2-thione **1a** was obtained by sulfurylation of the commercial 3-methyl-indoline-2-one 5a following the procedure described by Nirvard.[2] The substituted 3-methylindoline-2-thiones **1b–g** were synthesized by previous methylation on C3 of the commercial substituted 2-oxo indoles **5b–g** following the procedure reported by Kokatla,[3] and then then sulfurylated according to the method described by Nivard.[2] The 3-alkylindoline-2-thiones **1h–j** were obtained by C3-alkylation of the indol-2-one **4a** according to the method reported by Yuan,[4] and then sulfurylated according to the method described by Nivard.[2]

2) J. W. Scheeren, P. H. J. Ooms, R. J. F. Nivard, *Synthesis*, **1973**, 149.

3) S. Golla, S. Jalagam, S. Poshala, H. P. Kokatla, *Org. Biomol. Chem.*, **2022**, *20*, 4926).

4) W.-C. Yuan, J. Zuo, S.-P. Yuan, J.-Q. Zhao, Z.-H. Wang, Y. Youa, *Org. Chem. Front.*, **2021**, *8*, 784–791.

5) CrysAlisPro 1.171.42.90a (Rigaku Oxford Diffraction, Rigaku Corporation, Oxford, UK, 2023.

6) Burla, M.C., Caliandro, R., Carrozzini, B., Cascarano, G.L., Cuocci, C., Giacovazzo, C., Mallamo, M., Mazzone, A. & G. Polidori, G. (2015). *J. Appl. Cryst.* **48**, 306-309.

7) Sheldrick, G.M. Crystal structure refinement with SHELXL. Acta Cryst. 2015, C71, 3–8.

8). Farrugia, L. J. *J. Appl. Crystallogr.* **1997**, *30*, 567–567.
